# Predicting the Risk of Suicide by Analyzing the Text of Clinical Notes

**DOI:** 10.1371/journal.pone.0085733

**Published:** 2014-01-28

**Authors:** Chris Poulin, Brian Shiner, Paul Thompson, Linas Vepstas, Yinong Young-Xu, Benjamin Goertzel, Bradley Watts, Laura Flashman, Thomas McAllister

**Affiliations:** 1 The Geisel School of Medicine at Dartmouth College & The Thayer School of Engineering at Dartmouth College, Hanover, New Hampshire, United States of America; 2 The Durkheim Project, Portsmouth, New Hampshire, United States of America; 3 United States Department of Veterans Affairs, White River Junction VA Medical Center, White River Junction, Vermont, United States of America; 4 Novamente, LLC: Rockville, Maryland, United States of America; Dana-Farber Cancer Institute, United States of America

## Abstract

We developed linguistics-driven prediction models to estimate the risk of suicide. These models were generated from unstructured clinical notes taken from a national sample of U.S. Veterans Administration (VA) medical records. We created three matched cohorts: veterans who committed suicide, veterans who used mental health services and did not commit suicide, and veterans who did not use mental health services and did not commit suicide during the observation period (*n* = 70 in each group). From the clinical notes, we generated datasets of single keywords and multi-word phrases, and constructed prediction models using a machine-learning algorithm based on a genetic programming framework. The resulting inference accuracy was consistently 65% or more. Our data therefore suggests that computerized text analytics can be applied to unstructured medical records to estimate the risk of suicide. The resulting system could allow clinicians to potentially screen seemingly healthy patients at the primary care level, and to continuously evaluate the suicide risk among psychiatric patients.

## Introduction

Detecting individuals who are at increased risk of suicide is a major clinical challenge. Suicide among military personnel and veterans is a topic of international concern, and the U.S. Veterans Health Administration (VHA) has increasingly focused on suicide prevention [1], [2]. Clinicians generally ask patients whether they are “suicidal” and base their risk assessments primarily on the response. The concept of suicidality includes both thoughts about suicide and intentions to act on those thoughts [3]. While suicidality is a prominent risk factor for suicide attempts and completions, only approximately 30% of patients attempting suicide disclose their suicidal ideation [4], [5], [6], and the vast majority of individuals who express suicidal ideation never go on to attempt suicide [7], [8], [9]. Given this poor predictive value, clinicians might consider a more comprehensive approach by evaluating additional demographic risk factors for suicide.

Many of the risk factors for suicide, such as being an older white male [10], affect the majority of patients attending some VHA clinics [11]. Therefore, providing intensified monitoring for patients from specific demographic or clinical groups, such as veterans with depression, would require a major overhaul of VA services [12]. Some patterns of health services use are also risk factors for suicide. For example, Zivin et al. [13] found that veterans with recent VHA psychiatric hospitalizations were at a significantly higher risk of suicide. Close monitoring of individuals who have been hospitalized for depression could be accomplished with a modest additional expense [12]. However, new service demands could grow substantially if post-hospitalization monitoring protocols were extended to additional high-risk groups, and veterans at high risk of suicide who have never been hospitalized might be missed. Furthermore, additional monitoring visits during high-risk periods may not actually decrease the risk of suicide [14].

One potential reason for the poor effects of clinical monitoring in high-risk patients may be difficulty in identifying these patients. While currently-used assessment tools are based on recognized demographic, diagnostic, and health service use-related risk factors, recent systematic reviews have cited a lack of prospective studies evaluating the predictive accuracy of currently-available risk assessment tools [15], [16].Given this problem, completing comprehensive risk assessments may be time-consuming and detract from other important aspects of clinical visits without adding value for patients. Even if this process could be automated, recent findings indicate that the predictive value of combinations of suicide risk factors obtained from structured electronic medical records (EMR) fields become asymptotic as the risk conferred by multiple risk factors is less than the sum of each individual risk factor [17]. Therefore, the use of novel techniques to obtain additional information from unstructured aspects of the EMR may help to build more useful models of suicide risk.

## Methods

### Overview

Our goal was to develop a suicide risk classification tool using clinical notes. We sought to develop the prediction models there are obvious clinical applications of the approach. Specifically this or a similar model could be applied to a patient electronic medical record to aid clinicians in determining individual patients' suicide risk. Therefore, we conducted a case-control study to compare the clinical note text from a cohort of patients who committed suicide, with the notes from two cohorts of patients who did not commit suicide.

### Study Cohorts

To identify a suicide cohort, we used the VHA National Suicide Registry to obtain a random sample of 100 VHA enrollees who committed suicide in 2009. The VHA National Suicide Registry uses the Centers for Disease Control and Prevention (CDC) national death index (NDI) to verify that suicide is the cause of death. Because there are lags in the collection of death certificates by the CDC and in the VA records matching procedure, 2009 was the most recent cohort that we could obtain. The clinical notes from the 365 days preceding the suicide (up to the day before the suicide) were acquired from the VHA Corporate Data Warehouse (CDW). We then created two matched cohorts on the basis of sex, age, hospital where care was received, and patient disability status). Three cohorts were assessed: Cohort 1 included VA patients who did not use mental health services, Cohort 2 was the suicide cohort, and Cohort 3 included VHA patients who were hospitalized in inpatient psychiatric units at least once in 2009 but did not complete suicide. A total of 30 individuals in Cohort 2 had not used any VA health services in the year before their suicide, so no clinical notes were available from this period. Therefore, the final matched non-suicide cohorts comprised 70 patients each.

### Primary Data

Clinical notes that were written by nurses, doctors and other healthcare professionals were used as the primary data via the VA Electronic Medical Record. The notes described hospitalizations, procedures, surgeries, and other medical services. In addition to free text, the notes included semi-automatic, script-generated tables (e.g. lists of medications). Notes that discussed psychological state, depression and alcoholism were present for all three cohorts. On days when patients visited the VA facility, between 1 and 12 notes were written the subjects, with the larger note counts occurring during inpatient hospitalizations. The dataset for each group contained the following records: Cohort 1 had 1,913 notes (27 notes per patient), Cohort 2 had 4,243 notes (61 notes per patient), and Cohort 3 had 5,388 notes (77 notes per patient).

### Statistical Modeling

We performed the data analysis and built models of the datasets using supervised training with genetic programming, a specific type of supervised machine-learning system (i.e. a computerized system that can learn to recognize patterns associated with a known outcome.). The models were constructed by converting the free-text records into words or word phrases datasets, that is, numerical counts of how often a given word or phrase appeared in a patient record. The derived models then identified the combination of words that were associated with suicide. The data was analyzed using a machine-learning algorithm [18] to generate predictive models. By using the algorithm for each patient's notes, we first predicted whether the patient belonged to group 2 or group 3.

The model-building process consisted of several stages. In the initial stage, the free-text data were converted into a dataset of single words (bag-of-words) or phrases (bag-of-phrases). For simplicity, we primarily discuss the bag-of-words models, but experiments with both models are discussed in the Appendices. Bag-of-words modeling uses the frequency of words in a patient's medical report and completely disregards the linguistic structure, punctuation, and structural markup of the original text. Typically, 30,000–40,000 different words are identified in each dataset. The records are not spell-checked or stemmed (i.e. reducing derivatives of words to their stem), and can include typographical errors and abbreviations of hospitals, clinics, departments, tests, procedures, and orders.

The next stage consists of feature selection. Rather than directly training the discriminator on the full set of word counts, the set is reduced to several thousand words that are judged to be significant for the predicting outcome. This cut is accomplished by computing the mutual information (or dependence of variables) among the groups (1, 2, or 3) and the word counts. The few thousand words with the highest mutual information, or variable co-dependence, (MI) values [19] are then selected for the final model-building stage.

We then trained the machine-learning algorithm on a set of labeled examples (for Cohorts 1, 2, 3). Each example corresponded to a patient with a known category assignment and is presented to the machine-learning algorithm as a vector of selected features. As a result, a classification model was developed that was used to predict categories for new examples. Running the algorithm several times can produce many different models. The multiple “ensemble” models approach provides more reliable results than any individual model. To evaluate an ensemble of 100 models with 5-fold cross-validation, we trained a total of 500 models.

To display the risk for suicide, we used a 3 bin classification scheme. This system would allow clinicians to screen seemingly healthy patients at the primary care level, and clinicians could continuously reevaluate the risk among psychiatric patients. To accomplish a three-level classifier from the given datasets, we combined some of the datasets to form two binary classifiers. We achieved this using the following process. For cohort 1 versus cohort 2 and cohort 3 patients, groups 2 and 3 were combined, and a classifier was trained to differentiate group 1. If the classifier recognized a patient as belonging to group 1, the patient was marked group 1. For group 3 versus group 2 patients, groups 1 and 3 were combined, and a classifier was trained to differentiate group 2. If this classifier recognized a patient as belonging to group 2, the patient was marked as group 2; otherwise, the patient was marked as group 3. Eventually combining two groups increases the size of the training set, which would then significantly improve the accuracy of the scores and results in a Cohort 1 vs. Cohort 2 vs. Cohort 3 (1v2v3) classifier.

After an initial selection of the relevant single-word terms, we improved the model accuracy by using word pairs. A word pair was used only if one of the words in the pair already correlated well with the cohort. This step required an exclusion process in which we discarded word pairs with low MI values, infrequently occurring pairs and words, and word pairs that did not contain statistically significant values.

### Assessment and Validation

To determine the accuracy and performance of the classifier, we used standard *k*-fold cross-validation techniques. We divided the dataset into five parts (where *k* = 5), used four parts to train a model, and then measured the model accuracy on the fifth part. Each time we repeated the process, we excluded a different fifth of the dataset. We used the average of the five sessions as the overall accuracy.

### Informative Features

The final step of the analysis was to extract the predictive terms for each cohort. This step involved extracting the predictive terms from the models and then assigning the terms to the cohort from which they originated. Terms were yielded for each cohort. That is, that we selected only those high MI terms that occurred most in one risk group. We then sorted the terms by frequency of occurrence, and the terms were displayed as color-coded word clouds of single words.

## Results

Prior to the application of machine learning, we observed that the data from the third cohort (the psychiatric non-suicide cohort) had more extensive notes *per patient* and more terms of extensive psychiatric diagnosis than the data from the other two cohorts. That is, compared with the third cohort, cohort two (the suicide cohort) had fewer notes, and with a few exceptions, the notes described patient presentations that were similar to the presentations in cohort 1 (control) (see *Table 1*).

**Table 1:** Possible Relationships of Key Words and Known Domains of Suicide Risk Factors.

Domain	Association of Domain with Suicide (word frequency)		
	Known Link	Possible Link	Unknown
**Patient Behaviors**	Agitation (24)		
	Frightened (18)		
	Delusional (11)		
	Tense (7)		
	Aggravated (5)		
**Cardiac Conditions**		Vtach (15)	
		Tach (9)	
**Gastrointestinal Conditions**	Quadrants (11)		
	ALOH (10)		
	Subsalicylate (9)		
	MGOH (7)		
	Pylori (5)		
**Pulmonary Conditions**	Nebulizer (8)		
	Secretions (5)		
	Rhonchi (5)		
**Oncologic Conditions**	Terminal (10)		
	Unresectable (3)		
	Cancers (2)		
**Pain Conditions**	Analgesia (13)		
	Demerol (12)		
	Lumbago (5)		
**Care Descriptors**		Integrated (5)	Adequately (23)
			Standards (14)
			Clarify (7)
**Unexplained**			Format (8)
			Happens (8)
			Camera (7)
			Bottom (7)

**Figure 1:**
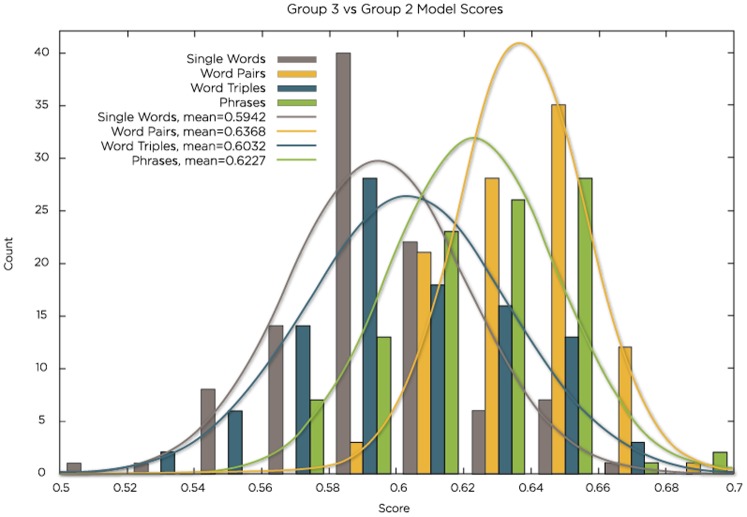
N-gram performance of the machine-learning algorithm applied to clinical notes. Where Count = Number of Models, Score = Accuracy, and the colors coordinate to model type.

For single-word models, the predictive accuracy was approximately 59% (the average for 100 models), and scores for individual candidate models ranged from 46–65%. Models that used certain word pairs had significantly better scores than single-word models, though they are far less human readable. The phrases “negative assessment for PTSD” and “positive assessment for PTSD” carry different meanings, this phrases-based approach was more accurate than a single-word approach. For pre-selected word pairs, the individual model scores ranged from 52–69%, with an average of 64% (for 100 models) (*Figure 1*).

In the final experiments, the combined Cohorts ‘1v2v3 classifier’ had a peak performance of 67%, and an average performance of 65%. For more information, see *Appendices 1 & 2*.

## Discussion

Our analyses were successful at determining useful text-based signals of suicidality. We obtained accuracies of greater than 60% for ensemble averages of 100 models, and our individual model accuracies reached 67–69%. Given the small size of the dataset and the fragmentary nature of the clinical notes, this performance level represents a significant achievement. For a classifier, these results represent a statistically significant ‘signal’. Meanwhile, we showed that methodologically word pairs are more useful than single words for model construction on EMR data.

**Figure 2:**
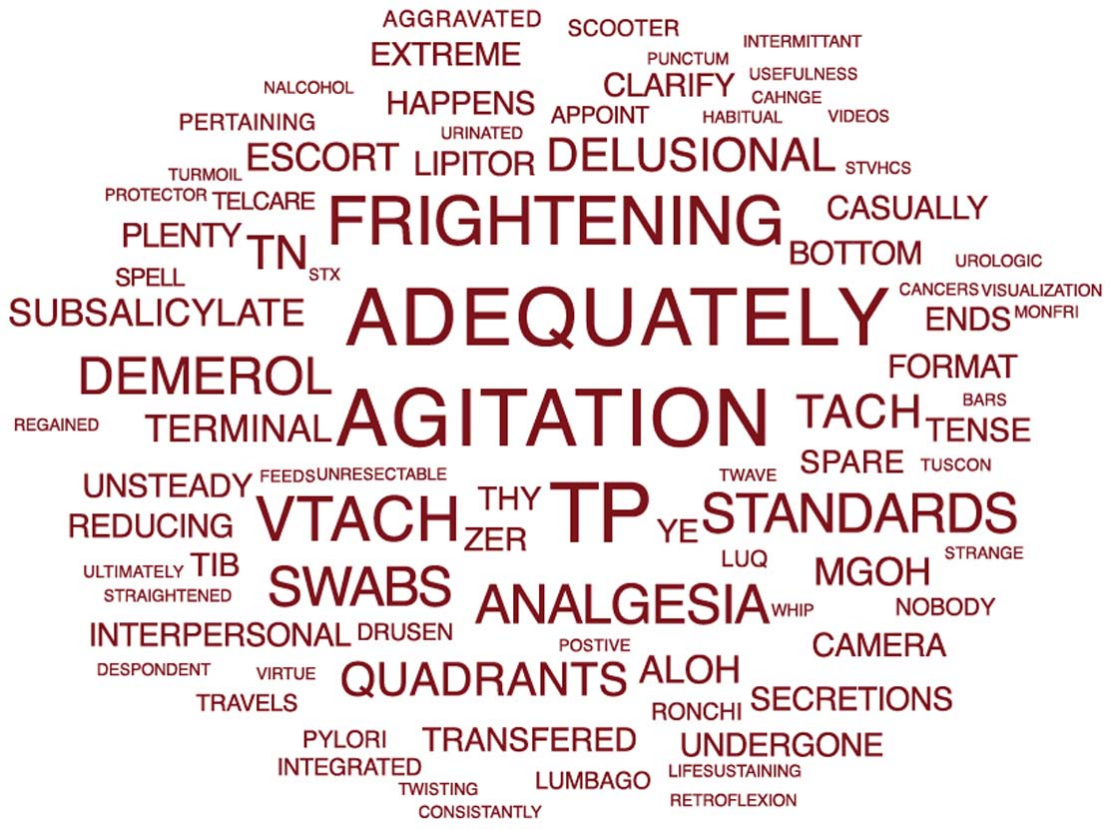
Terms displayed are those single words that were predictive for the suicide group (2).

Furthermore, the predictive feature words that distinguished each group were highly revealing, especially of the suicidal cohort (*Figure 2*), and were consistent with the existing medical literature on suicide (*Table 1*). We posit that the best explanations for the suicide group's predictive terms (*Figures. 2* *,* *3* *,* *4* *&* *Table 1*) relate to the medical literature's descriptions of patient behaviors and conditions that are frequently associated with suicide.

**Figure 3:**
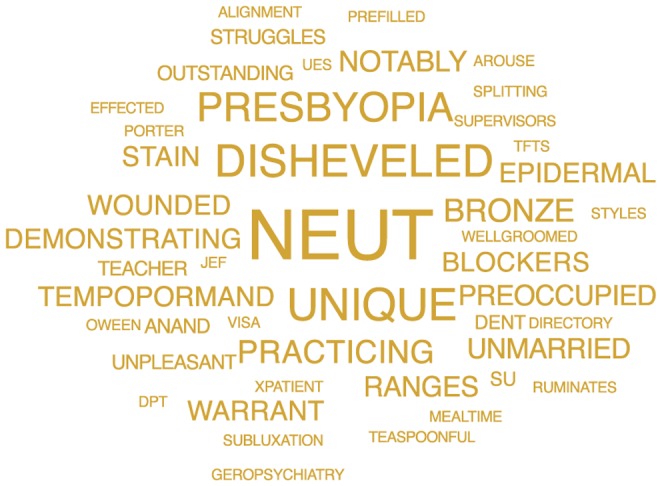
Terms displayed are those single words that were predictive for the psychiatric group (3).

**Figure 4:**
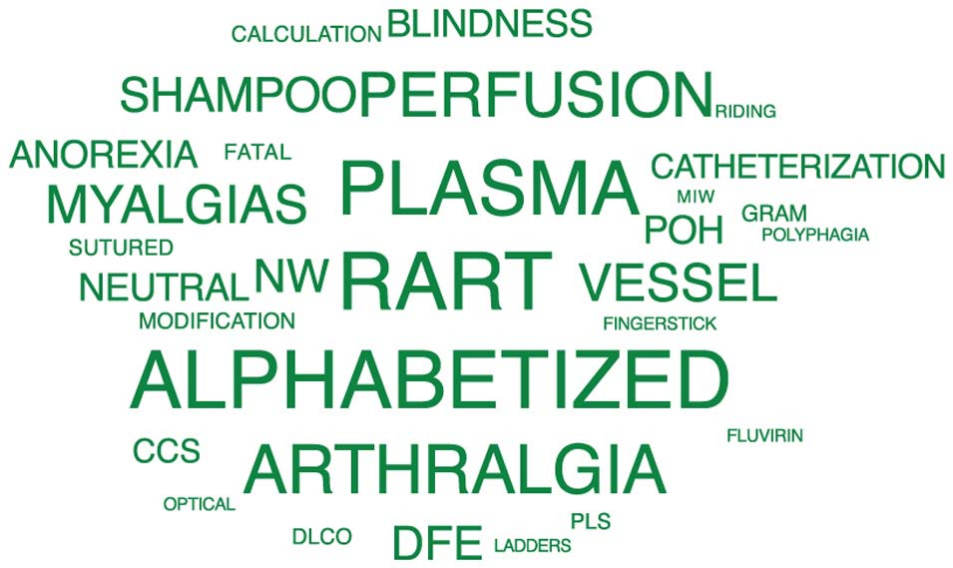
Terms displayed are those single words that were predictive for the control group (1).

The most common observation (words in a note that likely seems related to the clinician's description of the patient's behavior) was “agitation,” which has appeared frequently in the literature as a marker for suicide risk [20], [21]. Other behavioral descriptions have also been reported, including feeling frightened [22] and experiencing psychotic symptoms such as delusions [23], [24].

Many medical conditions have been associated with an increased risk for suicide, but these conditions have generally not been included in suicide risk assessment tools. These conditions include gastrointestinal conditions [25], [26], cardiopulmonary conditions [27], [28], [29], [30], oncologic conditions [31], [32], [33], and pain conditions [34], [35]. Finally, some research has emerged that links care processes to suicide risk. The word “integrated” emerged as a key term and is also reflected in the integrated care literature [36].

We note that limitations to our initial study are considerable: We based on model on only 210 subjects. And we would need further research on larger datasets in order to validate our findings before attempting clinical testing. It is unclear if the note text from these subjects is in any way representative of notes for patients in general. It is possible that the text in VA clinician's notes differs from other non-VA providers notes. Finally, our approach was retrospective by design and we have yet to demonstrate the ability of this approach to predict suicide prospectively in a clinical cohort.

In a follow up study, we will likely obtain better results by applying the same methodology to larger datasets, and by use of more complex linguistic analysis. However, this work shows that linguistic analysis of unstructured areas of the medical record, such as clinician notes, can be used for automated suicide risk assessment, and better targeting of suicide prevention resources.

### Predictive Results

The POSES prediction toolkit is a software system enabling streamlined application of the underlying MOSES algorithm to supervised classification and regression problems. MOSES is an automated program learning algorithm fusing ideas from genetic programming and probabilistic learning [18]. The resulting architecture is a high dimensionality classification paradigm that is optimal for the isolation of weak signals. A detailed mathematical account of the POSES/MOSES learning approach used to generate these models, and the appropriate interpretation of the models, may be found in the existing literature [37]. While POSES/MOSES is not the only possible way to analyze this data, it is important to understand that our accuracy levels (65–67%) on small data sizes, are as much due to the mathematical rigor of the POSES/MOSES classification scheme, as the quality of the underlying quality of the data set. Those that repeat this analysis with another system may well have poorer predictive results. As such, we have included these machine learning libraries as *Appendix S3*.

### Detailed Human Subjects Description

#### Approving institutional review board

This study was approved by the White River Junction VA Research and Development Committee, the Dartmouth Center for the Protection of Human Subjects (CPHS #23400), and the VA Office of Mental Health Operations (DUA# SHINER06212012). The Dartmouth College CPHS acts as the ethics committee for Dartmouth College, thus there was no requirement for additional ethics review.

- **Consent Type:** This was entirely retrospective research. Approving bodies granted a waiver of informed consent. As such, the requirement for informed consent was waived by the Dartmouth College CPHS. The White River Junction VAMC research and development committee and the VHA Office of Mental Health Operations concurred with this waiver as part of the study plan.
- **Methods of Categorizing:** Our case group was chosen at random from all known VA user suicides during the 2009 fiscal year. One control group was created to represent VA users who had not engaged in mental health services and had not died. One control group was created to represent VA users who had been hospitalized on inpatient psychiatry units and had not died. We matched our control groups on sex, age, hospital where care was received, and patient disability status.
- **Definitions and Categories in Detail:** Our suicide cohort was chosen at random from all known VA user suicides during the 2009 fiscal year. We identified our cases using the VA National Suicide Registry. The VA National Suicide Registry is maintained by the VA Office of Mental Health Operations. The registry identifies known deaths among VA users using the VA Vital Status File. These cases are cross-matched with the CDC National Death Index to determine cause of death. Our cases were drawn randomly from the subset of VA users whose cause of death was determined to be suicide. We identified our matched controls with service utilization records from the VA Corporate Data Warehouse during the year preceding each suicide. The VA Corporate Data Warehouse is a national repository of data collected using the Veterans Health Information Systems and Technology Architecture electronic health record system. Our non-mental health control controls contained VA users who had not had any outpatient or inpatient mental health visits. Our inpatient mental health control group contained VA uses who had been hospitalized on inpatient psychiatry units.
- **Choices of definitions and categories:** We chose our matched control cohorts for specific reasons. The non-mental health user group was chosen to represent a general population with a lower risk of suicide. The inpatient psychiatry group was chosen to represent a high-risk population.
- **Controlled for confounding variables:** To ensure that we could identify important differences between cases and controls, we created our matches based on a parsimonious list of covariates. We chose sex and age as these covariates have already been well-studied as predictors of suicide. We chose hospital where care was received in order to account for large variations in practice across the country. We chose disability status as a proxy for access to services, as disability status plays a prominent role in determining access to VA healthcare.

## Supporting Information

Appendix S1 **Data Analysis Methodology: This section expands on each step of the analysis in greater detail, and provides a detailed review of the model building and validation, feature selection, size and content of the clinical notes, results, and model accuracy estimation.**
(PDF)

Appendix S2 **Keywords: A group of files detailing the highest Mutual Information (MI) terms associated with each cohort's classification.** This is useful for training an alternative machine learning classifier, as well as for expert (clinical) analysis of risk factors. Specifically;Appendix 2.1: Features of highest correlation to suicide and low correlation to non-suicide (single + word pair combinations)Appendix 2.2: Features of lowest correlation to suicide and high correlation to psychiatric group (single + word pair combinations)Appendix 2.3: Features of lowest correlation to suicide and high correlation to non-psychiatric control (single + word pair combinations)Appendix 2.4: Features of highest correlation to suicide and high correlation to non-suicide (i.e. the Union of words from 2.1, 2.2, 2.3)Appendix 2.5: All Features, i.e. the Superset of 2.1–2.4 + those of low correlation to suicide and low correlation to non-suicide.
(ZIP)

Appendix S3 **Machine Learning Libraries and Methods: This section is provided to enclose the open source (Apache License) classifier used for the building of the statistical models, specifically for the purposes of study reproducibility.** A detailed account of these tools is intended for another publication.
(ZIP)
