# Correction: Predicting the Risk of Suicide by Analyzing the Text of Clinical Notes

**DOI:** 10.1371/journal.pone.0091602

**Published:** 2014-03-20

**Authors:** The PLOS ONE Staff

The first sentence of the Competing interests statement is incorrect. The Competing interests statement should read: "CP is President of Patterns and Predictions, whose company in conjunction with PT and LV, has a patent pending in relation to information discussed in the Appendix 1. There are no further patents, products in development or marketed products to declare. This does not alter our adherence to all of the PLOS ONE policies on sharing data and materials."
