# Supplementary material for: Predicting the Risk of Suicide by Analyzing the Text of Clinical Notes
Source: PLoS One. 2014 Jan 28;9(1):e85733. doi: 10.1371/journal.pone.0085733 (PMC3904866; doi:10.1371/journal.pone.0085733)
Supplement: Appendix S1 — Data Analysis Methodology: This section expands on each step of the analysis in greater detail, and provides a detailed review of the model building and validation, feature selection, size and content of the clinical notes, results, and model accuracy estimation. (PDF) [file pone.0085733.s001.pdf]

Appendix 1: Data Analysis Methodology

13 March 2013 (revised 23 October 2013)

Abstract

This report describes the suicidality prediction models created under the DARPA DCAPS program. The models were built primarily from unstructured text (free-format clinician notes) for several hundred patient records obtained from the Veterans Health Administration (VHA). The models were constructed using a genetic programming algorithm applied to bag-of-words and bag-of-phrases datasets. The influence of additional structured data was explored but was found to be minor. Given the small dataset size, classification between cohorts was high fidelity (98%). Cross-validation suggests these models are reasonably predictive, with an accuracy of 50% to 69% on five rotating folds, with ensemble averages of 58% to 67%. One particularly noteworthy result is that word-pairs can dramatically improve classification accuracy; but this is the case only when one of the words in the pair is already known to have a high predictive value. By contrast, the set of all possible word-pairs does not improve on a simple bag-of-words model.

Introduction

A central goal of the Durkheim project is to build a classifier for suicide ideation and prediction of suicide risk, based on free-text clinician notes contained in a set medical records obtained from the Veterans Health Administration (VHA), via the Dartmouth-Hitchcock Medical Center (DHMC). The intended use of the classifier is to aid the clinician in determining the suicide risk of prospective patients. As such, it should be able to digest patient data, and assign a risk level, green/yellow/red, suggesting the likelihood of suicidal ideation.

In order to understand how to build such a classifier, an extensive analysis of medical records of VHA patients were performed. Patient records were divided into three cohorts. These consist of a control group of 70 patients (group 1), a suicide cohort of 69 patients (group 2), and a psychiatric cohort of 70 patients (group 3). The medical records consist primarily of free-text notes entered by the clinician, as well as additional structured data (demographics, drug prescriptions, hospitalization admission records). The inclusion of the structured data in the training set makes for a very slight improvement of the overall score (fractions of a percent).

The clinician notes include both nurse and doctor notes, ranging from mundane procedures '*Patient received influenza vaccine per order*', descriptions of more serious procedures: '*ultrasound of the abdominal aorta done on...*', a number of semi-

automatic script-generated tables: '*Issue Date Status Last Fill Outpatient Medications (By Class) Refills Expiration*', as well as psychologically charged entries: '*Little interest or pleasure in doing things*'. Notes that discuss psychological state, including screenings for depression and alcoholism, appear in all three cohorts. One may presume that these are far more common, and delve deeper, in the last two cohorts. Aside from this kind of quick, cursory review to validate the general form of the records, no deeper review or examination was performed.

The data analysis was performed by using supervised training with a genetic programming system to build models of the datasets. The models were constructed by converting the free-text records into a 'bag of words': a simple numerical count of how often a given word appears in the context of a certain patient record. Any given model then identifies which words, taken in combination, serve as predictors of suicide. The nature of the genetic programming system used is that it can build many different models, depending on an initial random seed. Thus, data analysis consisted primarily of generating ensemble averages of models trained on the same dataset. Model validation was performed by using 5-fold cross-validation: that is, by setting aside 1/5 of the dataset for testing, and training on the remaining 4/5ths. Model accuracy was used as the score: that is, the total fraction of correct answers.

Most of the data analysis was focused on building a binary classifier to distinguish group 2 and 3. This was done for several reasons. One important reason was simply that these were the two largest groups, in terms of total word-count, and thus presented the greatest amount of data to work with. An equally important reason, though, is the clinical perception that these two groups are hard or even impossible to distinguish. By contrast, the control group consists of patients obtaining non-psychiatric medical care, and thus is almost completely devoid of references to psychological state. As such, it should be, in principle, easy to distinguish simply because it lacks this vocabulary. Results for binary classifiers trained to distinguish groups 1 vs. 2, as well as group 1+3 vs. group 2, are also presented.

Many of the words appearing in the models are emotionally charged or psychologically significant, such as 'PTSD', 'weapons', or 'overdose'. Taken individually, these words are meaningful, but not clinically out of the ordinary. Thus, a question arises: what phrases are these words a part of? Thus, a phrase such as 'negative assessment for PTSD' carries a different meaning than 'positive assessment', and is thus a potentially useful feature for classifying suicidal patients. This suggests that a 'bag-of-phrases' approach may be more accurate than a bag-of-words model, and this was indeed found to be the case. In particular, models built using certain word-pairs had significantly better scores than the single-word models, and had the best scores overall. Besides word pairs (bi-grams), trigrams and 4-grams were also explored, but these did not offer improvements, and it is hypothesized that the training datasets were too small to have a noticeable effect for these. The improvement for word-pairs is seen only when an initial selection or 'cut' is made: a word-pair is used only if one of the words in the pair already correlates well with the cohort. Without this cut, using word-pairs does not improve the score, and in fact lowers it: it is easier to over-train in such a case.

For single-word bag-of-word models, the accuracy, averaged over 100 models, was typically seen to be about 58% (depending on which cohorts were being distinguished), with low and high scores for individual models ranging from 46% (worse than ran-

dom chance) to over 65%. For selectively-chosen word-pairs, individual model scores ranged from 52% to 69%, with an ensemble average (for 100 models) of 67%. To the authors, this appears to be a remarkable achievement, given the small size of the dataset and the fragmentary nature of clinician notes.

The remainder of this document is structured to provide a mode detailed review of the model building and validation process, the size and content of the clinician notes, and the various results obtained.

Model Building and Validation

Model building consists of several stages. The initial stage converts the free-text data into a “bag of words”. This is simply a count of word frequency, and nothing more: a count of how often some given word was used in a particular patient’s medical report. Bag-of-words models completely ignore any sort of linguistic structure in the original text, as well as ignoring punctuation and any structural markup (paragraphs, sentence endings, *etc*.). Typically, 30 to 40 thousand different words were found, depending on which cohort is examined. These words were not spell-checked nor stemmed, and include many typographical errors as well as a large number of abbreviations for hospitals, clinics, departments, tests, procedures, and orders.

The next stage consists of ’feature selection’. Rather than training the discriminator directly on the full set of word counts, the set is reduced to the several thousand words judged to be most significant in predicting outcome. The cut may be done in several ways. One possible cut is to remove words that occur less than a few dozen times. Although the intent of this cut is to remove noise from the data, it is possible that perhaps some significant indicators are lost as well; thus data analysis includes experiments adjusting this cut. Another possible cut is to only count word stems: that is, to consolidate the counts for singular and plural forms of a noun, and to consolidate past, present and future tenses of verbs. The most important cut is to choose only those words whose counts correlate well with the patient grouping. This is done by computing the ’mutual information’ (MI) between the group id (1, 2 or 3) and the word-count frequency. The few thousand words with the highest MI are then selected to be used for the final model-building stage.

Feature selection is an important step of model building, and has a counter-intuitive effect on the final model: it is often the case that limiting the number of features used to build the model results in a better, more accurate model. This is because machine-learning algorithms can often focus in on irrelevant differences when classifying into groups: the differences are irrelevant, in that they fail to have predictive value. The greater the number of features (words) given to such a learning algorithm, the more likely it is to find such irrelevant differences; limiting the input to only the most significant features helps prevent such over-training.

Model building was performed using the poses/moses machine learning system[4, 3]. This system builds candidate representative models or ’representations’ of the data, and then uses evolutionary algorithms to discover the most effective representation. An example of such a representation, one of many, trained on the current data, is shown in Table 1.

Table 1: Example Representation

```
or(and(or(and($MODERATE_t1.3 !$PRESCRIBE_t0.02)
$CONCERN_t0.8 $EVIDENCE_t0.4 $INCREASING_t0.3
$RESTRICTED_t0.1) or($ALBUTEROL_t1.2 $AMOUNTS_t0.08
$SYSTEM_t0.08 $VIEW_t0.8) or(!$STOMACH_t0.4
!$SURROGATE_t0.7)) and(!$BRING_t0.6 !$HIGH_t1.9
!$MINUTES_t2.5 !$SAT_t0.7 $STOMACH_t0.4) $LOWEST_t0.08
$NYSTAGMUS_t0.03 $OLANZAPINE_t0.05 $OVERDOSE_t0.09
$PRESCRIBE_t0.02 $SUPERFICIAL_t0.16 $WEAPONS_t0.04
$WITHDRAWAL_t0.2)
```

The above is an example of a representation built from the VHA dataset. It may be understood as follows: `$MODERATE_t1.3` takes on a value of 'true' if the word 'moderate' occurs 1.3 or more times in the text (floating point values are used in case word-counts have been normalized to non-integer values). The exclamation mark ! indicates that the condition does not hold: so `!$PRESCRIBE_t0.02` means that the word 'prescribe' does NOT occur 0.02 or more times. The Boolean operators 'and', 'or' serve to conjoin these conditions: thus the above is saying that, "if the word 'moderate' appears at least twice, and the word 'prescribe' does not appear, or if any of the words 'concern', 'evidence', 'increasing' or 'restricted' appear at least once, and the word 'albuterol' appears at least twice ... then the patient should be classified as belonging to group 2." Note that, out of the approximately twenty-five thousand unique words appearing in the data, the above is really a rather very small subset.

The final classifier consists of not just one such representation, but many, ranging from one to over a hundred, depending on parameter settings. The predictions of each representative is used to cast a vote; the final determination follows from a tally of these votes. This process of ensemble averaging eliminates a considerable variation of accuracy from one model to the next[5].

To determine the accuracy and performance of the classifier, standard k-fold cross-validation techniques are used, with k=5. In this style of validation, the dataset is divided into 5 parts. Four of the parts are used to train a model, and then the accuracy of the model is measured on the fifth part. One then repeats this process, each time leaving out a different fifth of the dataset, to be used for evaluation. The average of the five sessions may then be given as the overall accuracy.

Almost all of the data analysis reported here was done by training the classifier to maximize accuracy: that is, to minimize the sum of the false-positive and false-negative rates. This is the appropriate approach when the datasets are balanced in size, as they are here. Alternatives to maximizing the accuracy would be maximizing the F_1-score or F_2-score, maximizing the recall rate, or the precision. None of these alternatives seem particularly suited for this dataset; they can lead to unexpected, imbalanced effects. For example, it will be seen later that it appears to be considerably easier to pick out patients with a low suicide risk out of a mixed population, than to pick out those with a

high risk; this is covered in a later section. However, for a general population wherethe suicide risk is very low, such ideas would need to be re-examined.

The remained of this document expands on each of the steps above in greater detail, describing feature selection, model building, and the estimation of the accuracy of the models.

Dataset statistics

The data consists of three sets of medical records:

- Group 1: The control cohort. These are the records of 70 patients who sought medical attention, but did not require or receive any special psychiatric treatment.
- Group 2: The suicide cohort. These are the records of 69 patients that committed suicide.
- Group 3: The psychiatric control group. These are records of 70 patients who sought help with psychiatric issues; they have not committed suicide, but may be at risk.

Associated with each patient is a set of note records, covering the span of one year. Records are generated for many reasons: upon hospital or clinic intake (by nursing staff); patient care notes (by the primary physician); examination results; lab results; consultation notes; notes from referrals, including imaging; outpatient notes; surgery and treatment notes; pharmacy notes; ongoing therapy notes; telephone follow-up notes; addenda and corrections. Thus, a single patient visit on a single day can generate from one to more than a dozen records.

The dataset is tokenized into a bag of words by converting all punctuation into white-space, and using white-space as word separators. The exceptions were word-phrases that included hyphens or underscores; this punctuation was simply removed to create a single run-on word. Differences in capitalization were ignored by converting all words to upper-case. After this normalization, the dataset was found to consist of nearly one million words; precisely, 971,189 words total. These are distributed across the three groups as follows:

- Group 1: 155,354 words, or 2,219 words per patient.
- Group 2: 350,435 words, or 5,079 words per patient.
- Group 3: 465,400 words, or 6,648 words per patient.

The number of words per record is fairly uniform across all three cohorts. Record lengths were limited to 1024 characters per record; it is clear that some of the longer records were truncated mid-sentence, mid-word. This appears to be due to technical interoperability difficulties with the VA data processing systems.

- Group 1: 1,913 records, or 27 records per patient, 81 words per record.
- Group 2: 4,243 records, or 61 records per patient, 82 words per record.

- Group 3: 5,388 records, or 77 records per patient, 86 words per record.

There were 24,860 unique words in the dataset that occurred at least once, but only 14,728 that occurred twice or more. A rough sketch of the distribution is given in table 2. Many of the words that appear only once are typos and miss-spellings of common words, abbreviations of medical terms, and a fair number of acronyms, including abbreviated names of clinics and hospital departments, lab procedures, orders and prescriptions. However, there are also many non-misspelled words that appear only once in the text, such as: ABANDONMENT ABORTIVE ABORTED ABUSER ABUSES ABYSS ACADEMY ACCUSE ACHIEVABLE ACHIEVES ACQUAINTED. Note that many of these words are emotionally meaningful words. Whether these infrequently-used can serve as indicators of psychological state is unclear. Experiments where low-frequency words are removed from the dataset before model building are reported below. At any rate, it is clear that the 'active vocabulary' of frequently used words is fairly small.

There was no attempt made to extract word stems, nor to correct or exclude 'obvious' miss-spellings. Whether doing so would enhance or diminish the ability to categorize is not clear *a priori*. No inclusion or exclusion criteria based on vocabulary were applied. Many different cuts, based on word-counts and mutual information, were explored, as detailed below. A feature selection stage applied prior to model building also effectively removes the majority of words from further consideration, but this cut is based purely on the predictive utility of a word, and not on its morphology, spelling, lexical meaning or usage.

Table 2: Word Distribution

Count	Number of occurrences
24861	once or more
14728	2 times or more
11613	3 or more
9928	4 or more
8844	5 or more
6862	8 or more
4618	16 or more
3042	32 or more
1928	64 or more

Dataset word distribution.

The most frequently occurring words are shown in table 3. Function words (the so-called 'stop' words) were not removed from the dataset, and thus appear in this table. There is a good reason for this: function words are known to be strong indicators of psychological state, and, in particular, the writing of suicides is known to make greater use of function words and pronouns than average[6, 1].

Table 3: Most Frequent Words

Word	$\log_2$ probability
TO	-5.191
AND	-5.542
THE	-5.568
OF	-5.755
FOR	-6.124
PATIENT	-6.151
HE	-6.418

The probability of a word is obtained by taking the number of times the word occurs, and dividing by the total word count. Here, $\log_2$ denotes the logarithm base-2. Thus, “to” occurs 26,588 times, or $0.027 = 2^{-5.191}$ fraction of the time.

The overall word distribution appears to obey the Zipf-Mandelbrot law (modified Zipf law), with a quadratic fall-off tail. This is more curved, and with a more quickly falling tail, than is commonly the case for natural-language texts. The distribution is shown in fig 1.

Word-pairs were also explored, as these have a predictive power as well. Word pairs were constructed by considering adjacent words, as well as pairs one word apart (ignoring the word in the middle). Thus, for example: "big red balloon" generates three word pairs: "big_red", "red_balloon" and “big_balloon”. The first of these pairs is not particularly meaningful, but both of the last two are semantic units. The last, “big_balloon”, would not have been captured if one confined oneself only to adjacent words. By eliding middle words such semantically significant pairs can be discovered.

Not all word pairs are equally interesting. Semantically meaningful word pairs are those with a high mutual information between them. Mutual information (MI) for a pair of words x,y is defined as

$$MI(x,y) = -\log_2 \frac{p(x,y)}{p(x,*)p(*,y)}$$

Here, $p(x,y)$ is the probability of seeing the word pair x,y, divided by the total number of word pairs. The two probabilities $p(x,*)$ and $p(*,y)$ are the probabilities of seeing any word pair, whose first word is x, or last word is y, respectively. In general, MI scores typically range from slightly above 20 to less than zero; the same is true of this dataset. In general, word pairs with a high MI form lexical units, conveying meaning, that is, having semantic content. They are collocations, often forming idioms and set phrases. Examples of word pairs with an MI of about 20, taken from this dataset, include ULTERIOR_MOTIVES, HLTHY_LVNG, VOCALIZES_INTELLIGIBELY, GIN_TONICS, ROAST_BEEF, MARATHON_RUNNER, GOVERNMENTAL_ENTITIES. By contrast, lower MI scores are less meaningful. Typically, the boundary between meaningful and meaningless word pairs occurs around an MI of 2 to 4. Examples of MI of 4 from this dataset include: HUNGRY_HAD, HAD_SWEAT, INTERACT_IN,

Figure 1: Word Rank Distribution

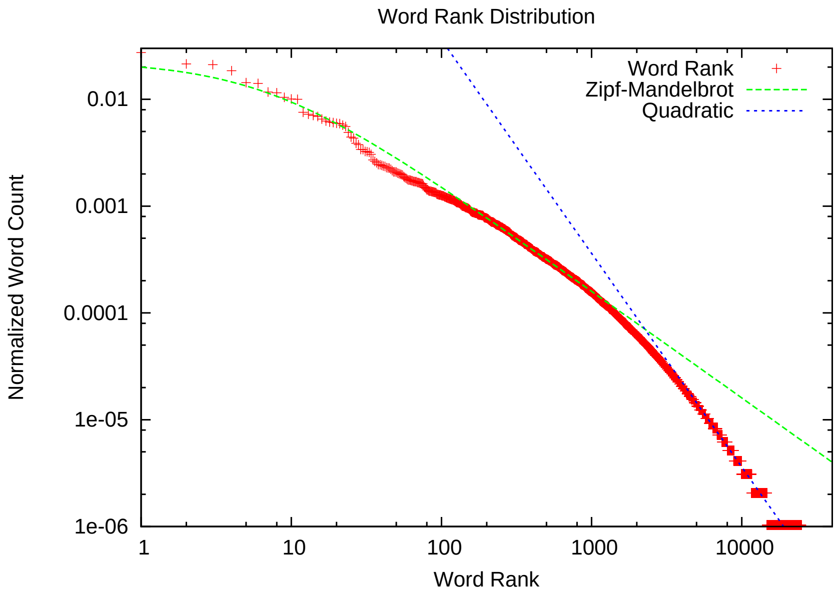

Word rank distribution for the dataset. The 'normalized word count' is the frequency with which a word appears in the dataset. The 'rank' is order of a word, when sorted by frequency. The green line indicates the Zipf-Mandelbrot law, here given as $0.16 \times (\text{rank} + 7)^{-1}$. The blue line is a quadratic fit, given by $360 \times (\text{rank})^{-2}$. The word distribution for most English-language texts (books, newspapers) is much flatter than that shown here. When word-pairs are incorporated into this ranking the curve also flattens and becomes less steep.

RX_IBUPROFEN, ANYTHING_HIMSELF. Those with an MI below zero degenerate into random nonsense: MORPHINE_YOU, RECOVERY_ARE, HIS_HOW, YES_WITH: pairs of words that appear next to one-another purely by coincidence, and not due to any linguistic construction. Thus, the mutual information can be used as a cut, to exclude low-MI word pairs from consideration during model building.

Results from models built from a variety of different MI cuts are presented below. Word pairs can be ranked along with individual words; the overall shape of the distribution does not change much; it is similar to that shown in figure 1, but considerably flatter, loosing the quadratic fall-off for low frequency words.

Model Building and Validation Details

The various stages of feature selection, model building and validation are each in themselves rather complex, and require some care to perform properly. None of the stages are 'pre-determined' or 'automatic'; instead, each has adjustable parameters and requires a deliberate choice of these parameters and overall configuration. Since the accuracy of the final classifiers depends on the various parameters settings in the data processing stages, it is important to understand what these are and how they are applied. The sections immediately below provide details describing these stages. This is followed by a presentation of the results obtained as these stages are applied.

Binning

Prior to performing training on the dataset, bin-counts are created. Binning helps to make up for relatively sparse data by lumping together similar word-counts into the same category or 'bin'. This serves to further simplify the data and boost the performance of the training step. It is performed by counting how often a word occurs for a given patient, and assigning it to a bin, such as 'occurs more than twice, but less than four times'. For a fixed set of bins, different patient records will be seen to contain different numbers of words in them.

A set of natural bin sizes can be obtained by first determining the probability distribution of a given word (over all patients); that is, by determining the average number of times it occurs (across all patients), and the standard deviation about this average (as it varies from patient to patient). These two numbers provide a natural size for a bin. For example, given an average number of times that a word occurs in a patient record, one may then say that, for a given patient, a given word occurs more than average, or less than average; in this case, there are two bins total. Another possibility is to use three bins: for a given patient, a word may occur about an average number of times (to within one standard deviation away from this average), or well-below average (more than one standard deviation below average), or well above average (more than one standard deviation above average). Bins serve to 'smooth' and consolidate word counts and make them more granular, to 'filter out high-frequency noise' from the data. In general, the less data one has, the fewer bins should be used, thus keeping the bins fairly full. Two to five bins may be considered; it will be seen, in later sections, that two bins work best for this dataset.

The result of binning are Boolean-valued features. So, for example, if the term 'PTSD' occurs an average of 2 times per patient record, a two-bin system would create one feature for this word: $(PTSD > 2)$ which is either true or false for a given patient record. If, for example, the standard deviation was 1.0 for this word, a three-bin system would include two features for this word, set at one standard deviation above and below average; that is, $(PTSD > 1)$ and $(PTSD > 3)$, each of which may be true or false for any given patient record. The values '2', '1', '3' shown here are referred to as 'thresholds': they are the boundaries between the bins. Thus, specifying N thresholds results in N+1 bins.

The number of thresholds to use is a parameter that can be specified; varying this parameter results in models of varying accuracy. The number of thresholds used is the same for all word counts: thus, setting thresholds=1 specifies that two bins are to be used for *all* words. So, for example, given 31 thousand distinct words, a two-bin system would create 31 thousand (true-false) features, while a three-bin system would result in twice as many: 62 thousand Boolean-valued features. A four-bin system would result in three times as many features, and so on. As is clear, increasing the number of thresholds vastly increases the dimensionality of the feature space.

Feature Selection

After binning, but before building a model, the dataset, now converted into a collection for true/false bin assignments, is run through a static feature-selection stage. This is done to reduce the size of the dataset, from tens of thousands of features, to a few thousand. The goal of this reduction is simply to improve the run-time and memory usage of the model-building stage.

Given that the overall dataset consists of only a few hundred records, it may seem reasonable that at most a few hundred features would suffice to provide predictive value; and indeed, the final models consist of dozens of words. However, the run-time speed of the next stage, model-building, is not strongly affected by the number of features that it is given, and so it was deemed safer to err on the side of giving it too many features to choose from (thousands), rather than too few (hundreds). Because of this, a very simple and efficient feature selection algorithm suffices. The algorithm used is to choose those features that have the highest mutual information with the desired patient classification. The mutual information is defined in the same way as before:

$$MI(x,y) = -\log_2 \frac{p(x,y)}{p(x,*)p(*,y)}$$

except that here, the variable x is taken as the classification of a patient belonging to one group or another, while the variable y is taken to denote whether a given feature is true or false. Thus, if a certain feature is true whenever the patient belongs to group A, we expect $MI(A,true)$ to be large; likewise, it may anti-correlate: $MI(A,false)$ may be large. To continue with the previous example, the mutual information content $MI(\text{``}group\,1\text{''}, (PTSD > 2))$ is computed for the word 'PTSD'. If this MI ranks in the top few thousand, then $(PTSD > 2)$ is accepted as a valid feature worth exploring during the training stage. Words that occur equally often in one group as another will have

a low MI score, and thus will not be selected. In most of the data analysis presented below, the highest-ranked 3000 features were selected. This represents anywhere from 2% to 10% of the total number of features, depending on the number of bins chosen, and the particular datasets examined.

There is no particularly strong reason for choosing MI, as opposed to some other correlation measure, such as tf-idf. MI has a strong mathematical foundation rooted in maximum entropy principles. It does not discriminate against rare words; a word which occurs infrequently but still correlates well with the patient grouping will have a reasonable MI score, and thus will be eminently suitable for use in a classifier. In any case, the particular choice of a feature selection algorithm should have little impact on model building.

Model Building

The most technically difficult and CPU intensive stage of the processing is the creation of models of the data. This step is performed by the Meta-Optimizing Semantic Evolutionary Search (MOSES) system. This system searches through a very large set of representations, such as that shown in table 1, and locates those that most accurately fit the training data.

The MOSES algorithm consists of two nested loops: representation-building and genetic-algorithm search[4, 3]. The system starts by creating a program tree (in the current usage, a tree of Boolean operators, such as that shown in table 1). The nodes and leaves of the tree are free to vary over the full range of input variables, as well as to vary over the Boolean operators (*and, or, not*). For any fixed choice of nodes and leaves, the resulting tree may be scored against the input training data (the features) to see how well it fits; clearly some choices will be better than others. The set of node and leaf settings are explored using a genetic evolutionary search algorithm combining hill-climbing and genetic cross-over. When no further improvements are found, the process is begun again, this time with a different, and usually, a more complex program tree. This step is again repeated until either a perfect score is reached, or set time-limits are exceeded.

The generation of candidate program trees involves a second, 'dynamic' feature-selection stage. A new candidate tree is created from an older high-scoring tree, by decorating it with additional candidate features. Rather than creating a candidate program tree with all of the several thousand features in it, convergence can be improved by working only with those features that add new information to those that are already in the tree: that is, by working with those features most likely to improve the current high-scoring tree. This is again a form of feature-selection, hereinafter referred to as 'dynamic feature selection', as the selected features depend on the program tree as well as the dataset, and a different set is chosen for each program tree. Training can be effective even with a very small number of dynamically selected features: best results are achieved with less than one hundred, and the technique is highly effective with as little as five! Aside from improving scores, working with a smaller number of features dramatically reduces training time.

The result of this process is a large number of representations, each of which model the training data more or less equally well. Each representation may be used to clas-

sify new patients (patients not in the training set); that is, to make predictions about the value of the dependent variable (the patient classification) based on input variables (word counts). When tested on a test set of patients held out from the training group, it can be seen that the accuracy of these representations on the test data is considerably variable. There is no *a priori* way of knowing which representation performs 'the best' on the test data. To overcome this variability, an *ensemble* is created, with each representation in the ensemble getting a vote to determine the final classification. That is, the same inputs are presented to each representation, with each representation making a prediction: a majority vote is then taken to determine the final classification. This ensemble is referred to as the *model*, as it is effectively a distilled, compressed version of the training data.

The theoretical validity of using the model for classification in this way is founded on the belief that the model captures something essential about the way that words are used in text. This is a reasonable belief, given industry experience with bag-of-words classifiers. The practical validity of the model can be tested in several ways; k-fold cross-validation will be used here.

Cross-Validation

In order to test the validity of the models, k-fold cross-validation is performed, with $k = 5$. The input dataset is split into k subsets, with each subset containing $1/k$ of the patient records, assigned by round-robin selection. Training is then performed using $k-1$ of these subsets as input, and a model is built (that is, a model is build on 4/5'ths of the data). The accuracy of the model is then evaluated on the subset that was held out (on the remaining 1/5th). This process is repeated k times, to obtain k models, and k different accuracy test results. The test results are then averaged together to obtain an estimate to the overall system accuracy. That is, if a model were trained on the full data-set (without any hold-outs), the accuracy of this resulting model, on new, blind data, is expected to be similar to the cross-validated accuracy. The effects of choosing different values of k are explored in a later section.

During cross-validation, four different statistics are gathered: the number of true-positives (TP), false-positives (FP), true-negatives (TN) and false-negatives (FN). All models were built as binary classifiers, so that 'positive' refers to membership in cohort 2: the suicide cohort. Thus, in this case, false-positives are those who were incorrectly classified as suicidal, whereas false-negatives are patients whose suicide was not foreseen. These four statistics can be presented in the form of a two-by-two table, termed the 'confusion matrix'. An example of such a matrix is shown in table 4.

In order to be a clinically useful system, it is probably best that, if the system erred, it did so by finding too many false positives, rather than by failing to detect a suicidal patient (a false negative). There are five different result variables that capture this idea in different ways: the 'recall', 'precision', 'accuracy', F_1-score and F_2-score. The 'recall' addresses the question "were all true positives identified (at the risk of some false positives)?" The 'precision' is the opposite: "were false positives minimized (at the risk of failing to identify some true positives)?" Accuracy, F_1 and F_2 are different ways of blending these together to obtain reasonable composite scores. Presuming that having a high recall is the clinically desirable way to classify patients, the F_2-score

Table 4: Example Confusion Matrix

	Predicted negatives	Predicted positives
Expected negatives	Numb. of true negatives (TN)	Numb. of false positives (FP)
Expected positives	Numb. of false negatives (FN)	Numb. of true positives (TP)

Results are reported in the form of the above confusion matrix. Scores for this matrix are given by the equations (1).

---

is then probably the best quantity to maximize. Note that maximizing F_2 can hurt accuracy (*i.e.* too many false positives), while maximizing accuracy can lead to more false-negatives than might be desirable.

Mathematically, these five quantities are defined as follows. These are the standard textbook definitions.

$$
\begin{aligned}
recall &= \frac{TP}{TP+FN} \\
precision &= \frac{TP}{TP+FP} \\
accuracy &= \frac{TP+TN}{TP+FP+FN+TN} \\
F_1 &= \frac{2\times precision\times recall}{precision+recall} \\
F_2 &= \frac{5\times precision\times recall}{4\times precision+recall}
\end{aligned}
\tag{1}
$$

Here, TP stands for 'true-positive', as above. All five quantities can vary between 0 and 1. For a system with a perfect score, all five quantities would equal 1. If all classification was done by random chance, then all recall and accuracy would equal 0.5 (for binary classification) and precision would be the fractional size of the positive group (0.5 if the positive and negative groups are identical in size). As most of the data analysis concerned groups that were equal in size, it is desired that all five quantities should be above 0.5. Note that it is possible to have a classifier that simultaneously scores above 0.5 for some of these measures, and below 0.5 for others.

Ensembles and Voting

In what follows, the concept of an *ensemble[5]* will be used in two related, but rather distinct ways. In the first sense, already discussed above, a *model* consists of an ensemble of *representations*; each representation gets a vote to determine the final classification that a model makes. In this construction, the nature of the individual representations can remain rather opaque, as their effect on the final classification is indirect. In order to gain more insight into how individual representations combine to form an ensemble, a restriction is made, in most of what follows, to limit each model so that

it holds only a single representation. Thus, in most of what follows, the ensemble is overt, and its behavior is overtly, explicitly presented. That is, the distribution of the classifications made by each representation, the average behavior, and the variance, is explicitly presented. Since each model holds only one representation, the ensemble is referred to as an ensemble of models.

However, in the end, one wants to revert to the intended purpose of the ensemble, which is to improve accuracy by combining multiple representations into one model, and performing classification by majority vote. In this case, the accuracy of a model will presumably depend on the number of representations within it. An exploration of how this accuracy depends on the size of the ensemble is given in the final sections. To summarize, there are two ensembles: the ensemble of representations comprising a model, and the ensemble of models.

Ensemble Formalities

This section sketches a formal, mathematical development of the ensemble classifier.

Let $P(g|p,m)$ be the probability that a given model m will classify a given patient p into group g. For a fixed patient and model, this probability is either zero or one (the classifier either assigns the patient to group g or it doesn't), so that $P(g|p,m)$ is just the set-membership function:

$$P(g|p,m) = \mathbf{1}_{g|p,m}$$

The classifier may be incorrect in it's assignment, of course. In what follows, it is presumed that all classifiers are binary, so that the group g ranges over the values $\{pos, neg\}$ denoting that a patient does or does not belong to the group. The law of the excluded middle is assumed:

$$P(g = pos|p,m) + P(g = neg|p,m) = 1$$

that is, a given patient is classified as either positive or negative.

If the patients are divided into a training set and a test set, and the classifier is trained on the training set, then $P(g|p,m)$ can be directly measured and evaluated on the test set. Let S_{pos} and S_{neg} be the sets of patients in the test set that are positive or negative for belonging to the group g. Then a given classier m gives the following counts for true positives (TP), *etc*:

$$TP = \sum_{p \in S_{pos}} P(g = pos|p,m)$$
$$FP = \sum_{p \in S_{neg}} P(g = pos|p,m)$$
$$FN = \sum_{p \in S_{pos}} P(g = neg|p,m)$$
$$TN = \sum_{p \in S_{neg}} P(g = neg|p,m)$$

The above formulas merely provide a more formal definition connecting two different notations for the same concepts, and nothing more.

The ensemble average is given by

$$P(g|p) = \frac{1}{|M|} \sum_{m \in M} P(g|p,m)$$

where M is the set of models making up the ensemble, and $|M|$ is the size of this set. In essence, the ensemble average is an expectation value. Note that the ensemble average is now a real-valued quantity, ranging over the interval [0,1]. The *poses* inference command uses the ensemble average to perform classification, and reports the average itself as the '*confidence*' of the inference. Specifically,

$$infer(p) = \begin{cases} pos & \text{if } P(g = pos|p) > 0.5 \\ neg & \text{if } P(g = pos|p) < 0.5 \end{cases}$$

and

$$confidence(p) = \begin{cases} 2P(g = pos|p) - 1 & \text{if } P(g = pos|p) > 0.5 \\ 2P(g = neg|p) - 1 & \text{if } P(g = neg|p) > 0.5 \end{cases}$$

Note that this is closely related to the accuracy (equation 1 above) on the test set:

$$accuracy = \frac{1}{|S|} \left[\sum_{p \in S_{pos}} P(g = pos|p) + \sum_{p \in S_{neg}} P(g = neg|p) \right]$$

where $|S| = |S_{pos}| + |S_{neg}|$ is the size of the test set.

Results Overview

A number of different data analysis experiments were performed. These include the effect of tuning adjustable parameters on the machine-learning system, the exploration of ensemble averages, the examination of the words that appeared in actual models, the effect of data cuts (*i.e.* excluding infrequent words from the models), and the predictive value of word-pairs, trigrams and 4-grams.

Example Model

Running the the classifier once, for a given set of parameters, results in a single model being created. The precise model, and its accuracy, depends on the training parameters, such as run-time, the number of features selected, the number of representations comprising the model, and other variables. In all cases (for all parameter settings), the resulting model fits the training data very well. One such case, typical of all, is shown in table 5. When this model is evaluated on the test set, the accuracy and other measures are, of course, sharply lower. In essence, the model is over-fit on the train set. The results for the best-fit model on the test set are shown in table 6.

Table 5: Training Confusion Matrix, Group 1 vs. Group 2

	Predicted Grp 1	Predicted Grp 2
Expected Grp 1	277	3
Expected Grp 2	11	265

Confusion matrix, in the form of table 4, for the training set. The model predictions are shown in the columns, the expected results in rows. There are $4 \times (70+69) = 556$ training records to be classified in a 5-fold cross validation.

Accuracy	0.9748	(542 correct out of 556 total)
Precision	0.9888	(265 correct out of 268 total)
Recall	0.9601	(265 correct out of 276 total)
FP Rate	0.0107	(3 false pos out of 280 total)
F_1 Score	0.9743	
F_2 Score	0.9657	

The results shown here indicate that the model that was created fits the training data very well, excelling in all measures. This is to be expected for the training set. The data shown are for a classifier that distinguishes groups 1 and 2, trained on the bag-of-words dataset. There were 3000 features pre-selected, 240 features dynamically selected, and two word-count thresholds used. In practice, these parameters have almost no effect on the above results: essentially all parameter settings result in very similar measures.

Table 6: Test Confusion Matrix, Group 3 vs. Group 2

	Predicted Grp 1	Predicted Grp 2
Expected Grp 1	43	27
Expected Grp 2	22	47

Confusion matrix for the test set. The model predictions are shown in the columns, the expected results in rows. There are $70+69=139$ test records to be classified in a 5-fold cross validation.

Accuracy	0.6475	(90 correct out of 139 total)
Precision	0.6351	(47 correct out of 74 total)
Recall	0.6812	(47 correct out of 69 total)
FP Rate	0.3857	(27 false pos out of 70 total)
F_1 Score	0.6573	
F_2 Score	0.6714	

The results shown here are for a model trained on a set of 3000 pre-selected features, dynamically narrowed to 500 features during the run. Input features were created by partitioning the word-counts into 2 levels, with a threshold at the word-count average. This model was selected to maximize accuracy, rather than recall or F_2 score; however, it appears to have the best F_2 score of all those explored.

Each model consists of ten representations, each resembling that shown in table 1. Given these representations, positive and negative keywords can be extracted. Positive keywords are those that appear in the target group (here, group 2), but not (as frequently) in the control group. Negative keywords are the reverse: they appear more frequently in the control group than the target group. A set of positive keywords, distinguishing groups 2 and 3, are shown in table 7 and a set of negative keywords in table 8.

Note also that a fair number of the keywords apper to be typographic errors, or are otherwise relatively rare. This can be easily explained: rare words will appear in relatively few records, and thus their presence gives an immediate mechanism with which to identify those records. Unfortunately, this also means that such keywords also have a poor predictive value: the fact that some word was mis-spelled in a particular patient record is very unlikely to be of any future use in classifying other patients. However, it can also be counter-productive to exclude keywords becuse they do not seem to be obviously relevant. For example, 'ALBUTEROL' sometimes appears among the postive keywords; superficially, it is an asthma medication, and thus non-predictive and irrelevant. However, it is also well-known to be associated with suicide risk. Telling noise from data by examining keywords is not an easy task. The role of infrequent words is explored in a later section.

Table 7: Positive Keywords, Group 3 vs. Group 2

AA AAA ABOUT ADULT ANTIPSYCHOTIC APPOINT BARS CAHNGE CANCERS CONSISTANTLY DESPONDENT DISORDER DRUSEN FRIGHTENING HC ICDCM INTERMITTANT LIPITOR LUQ MONFRI NALCOHOL NOBODY PRIVATE PUNCTUM REGAINED REORDER RESTRICTED SHAVE SPARE SPELL STANDARDS STRAIGHTENED STRANGE STREET STVHCS STX SUBSALICYLATE SWABS TACH TE TELEMETRY TEMAZEPAM THY TIB TP TRANSFUSIONS TRAVELS TURMOIL TUSCON TWAVE UC ULTIMATELY UNCOOPERATIVE UNDERGONE UNRESECTABLE URINATED VALLEY VIDEOS VISUALIZATION VTACH WATCHES WHEN WHIP WORTHLESSNESS WTIH YE YOUNGER ZER

The above is a list of positive keywords that distinguish groups 2 and 3. That is, the model requires that these words appear more frequently in group 2 than group 3. Note the appearance of a fair number of emotionally laden words. Not all models result in this particular word-list; differences between different models is discussed in a later section.

Table 8: Negative Keywords, Group 3 vs. Group 2

AFRAID AGGRAVATING ALIGNMENT ALOH ALOT ANT AROUSE BLOCKERS BRONZE CRYING DEMONSTRATING DENT DIRECTORY DISHEVELED DOCUSATE DPT EFFECTED EPIDERMAL FEAR GEROPSYCHIATRY MEALTIME NEUT NOCTURIA NOTABLY OBESITY OUTSTANDING PRACTICING PREFILLED PREOCCUPIED PRESBYOPIA PSYCHIATRICALLY QUADRANTS RANGES RESIDENTIAL RUMINATES SPECIMEN SPLITTING SSN STAIN STRUGGLES STUDENT STYLE STYLES SU SUBLUXATION SUPERVISOR SUPERVISORS SUPPLY SYMPTOMS TEACHER TEASPOONFUL TEETH TEMPOPORMAND TFTS THI TON TOP TOPICAL TRAZODONE UCINATIONS UES UNCHANGED UNHAPPY UNIQUE UNMARRIED UNPLEASANT UNSP UNT UPCOMING USEFULNESS VERIFIED VET VIRTUE VISA VISIT VIT VOIDING VOLUME WALKIN WARNING WARRANT WELLGROOMED WILLING WOUNDED XPATIENT YEAR

The above is a list of negative keywords that distinguish groups 2 and 3. That is, the model requires that these words appear less frequently in group 2 than group 3. Given that group 3 is the psych patient group, it is not surprising that many of the words seem to have a psychiatric significance.

Ensemble Averages

The space of all possible models of a dataset is astronomically large, and cannot be exhaustively searched. The moses/poses system uses a pseudo-random number generator to explore different parts of the search space, both during the genetic algorithm part of the search, and also during representation construction. The resulting final model thus depends on the initial random number seed; how well the model scores will as well. It is not clear how well the score of an individual model can be trusted, as there is no *a priori* argument that it will always extend in a good way over a larger dataset. To mitigate this uncertainty, an ensemble average may be used. In this case, the average of a large number of models, each built with a different initial random number seed, may be used.

In much of what follows, ensemble averages will be used. In all cases, 100 distinct models are built. The figure 2 illustrates how this works: it shows a bar-graph of the accuracy scores of 100 different models created with the same parameters and the same dataset, differing only in the initial random number seed. The figure shows a Bell curve fit to this data. A later section looks at model differences in greater detail.

Cross-Validation

Classifier performance depends strongly on the choice of the training set. To obtain an idea of how the training set influences accuracy scores, several different experiments were performed, summarized in table 9. In all cases, a total of ten different training/test set partitions were created, by performing a random draw (that is, patients were chosen randomly to belong to either the training or the test set). This allows the average accuracy to be obtained across the ten different test sets, as well as the standard deviation of the distribution. The data presented is for a bag-of-word-pairs model, which is presented in greater detail in later sections.

The overall suggestion from table 9is that maximizing the size of the training set, and then making up for the small size of the test set by averaging over many partitions, is the best strategy. For the remainder of the analysis, an 80/20 split, averaged over five round-robin partitions, will be used: this is the 5-fold cross validation.

Parameter Tuning

One of the most time-consuming experiments is to determine the optimal settings for the training parameters. The two most important and sensitive of these are the number of bins chosen for word-counts, and the number of dynamic features. The size of the static feature list seems to have little bearing on the ultimate score, once this is reasonably large; a static feature set of 3000 seems to be sufficient. Nor does the total training time seem to matter much, once it is sufficiently long. Increasing the training time will cause the system to build ever-more complex models, attempting to attain a perfect score on the training set. These more complex models do not appear to score better on the test set, nor do they appear to score any worse, either.

One noteworthy effect, though, is that the larger the dataset size, the less sensitive the results are on these adjustable parameters. Roughly speaking, the parameters are

Figure 2: Ensemble Average

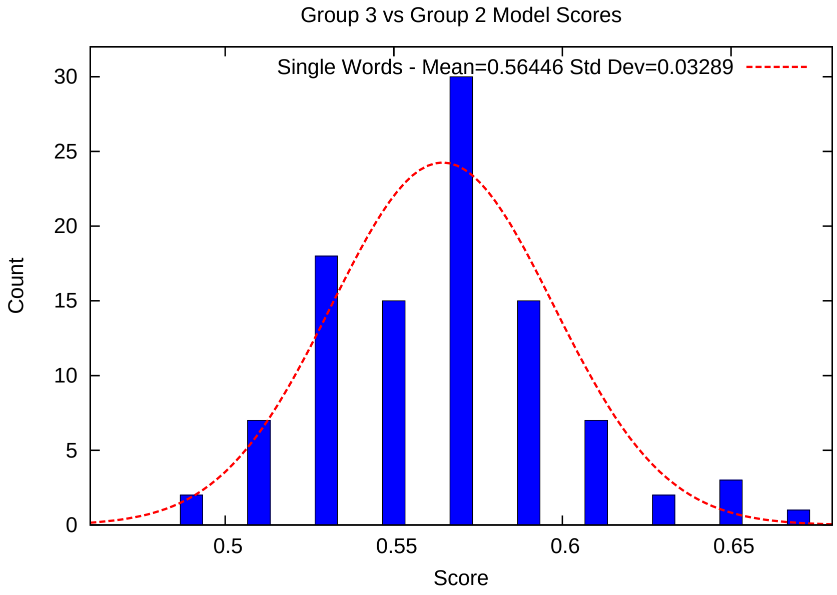

This bar chart shows the distribution of model accuracy scores for 100 models built to distinguish between the cohort 2 and cohort 3 for the bag-of-words dataset. The accuracy scores of each model was assigned to a bin that is 0.02 wide; thus there are five bars between 0.5 and 0.6 in this graph. The models were trained with with a single-word bag-of-words dataset, with word-count thresholding into 4 bins, and 240 dynamically chosen features, out of an initial feature set size of 3000. The fitted curve is a Gaussian, with a mean of 0.5645 and a standard deviation of 0.0329. This graph suggests that the 'typical' accuracy of a single model is then 56.45%, although there are a few models that score exceptionally well, including five models with an accuracy of 64% or better. It is not clear that the distribution is in fact Gaussian; it is possible that a log-normal distribution would provide a better fit. Note that a log-normal distribution would be centered at the location $\mu = 0.5728$.

Note that this same bar chart is shown again in figure 6, where it is compared to other models. Although this shows the best bag-of-words model, it is outperformed by all bag-of-phrases models.

Table 9: Cross-Validation Performance

	Mean Accuracy	Std. Dev.
80/20 set 1 r0	0.635	0.098
80/20 set 1 r1	0.573	0.075
80/20 set 2	0.612	0.081
75/25	0.560	0.114
66/33	0.573	0.103
50/50	0.559	0.0510

Average accuracy and standard deviation over ten different training/test set partitions. The 80/20 partition allocates 80% of the patients to the training set, and 20% to the test set. Three different experiments are shown: "set 1 r0" and "set 1 r1" both use exactly the same partitions, but initialize the learner with two different random seeds. The "set 2" experiment explores accuracy over a a completely different set of ten partitions. The rows labeled 75/25, 66/33 and 50/50 explore the effects of reducing the size of the training set.

---

used to 'focus' on the distinctive parts of the dataset, in much the same way that image processing is used to sharpen an image. For the larger datasets, there seems to be less of a need to 'focus'; but whether this is a real effect or an artifact is unclear. All of the datasets are small, and the largest dataset is about three times the size of the smallest one.

In order to evaluate the effect of this parameter tuning, ensemble averages, over 100 models, were used, as described above. For each set of parameters, the mean and standard deviation of the accuracy distribution was computed. These, as a function of the parameters, are shown in table 10. The figure 3 shows three typical distributions from this table, one of which was already shown in figure 2.

Infrequent Words

Infrequently occurring words appear to play an important predictive role. By 'infrequent' it is meant words that appear in less than a fourth of the patient records, and possibly in as few as just two. This is quite a remarkable result, and it manifests itself in several ways in the data. It raises questions: is this an artifact of working with sparse data, or is it possible that suicidal patients present in a variety of ways, with no common set of symptoms? This section explores how infrequent words influence model construction and model accuracy.

When using ensemble averages, the question arises: how similar or different are the individual models? Do they differ only a little, or a lot? Measuring precise differences is difficult, due to the fact that the actual representations (table 1) are Boolean program trees. However, a general indication can be had by generating keyword lists, and comparing them between models.

This was done by generating N different models, with each model consisting of 10

Table 10: Tuning the classifier, Group 3 vs. Group 2

Feat	Num Thresh=1		Num Thresh=2		Num Thresh=3	
	Mean Acc	Std Dev	Mean Acc	Std Dev	Mean Acc	Std Dev
40	0.5688	0.0234				
50	0.5803	0.0243				
60	0.5901	0.0261				
75	0.5942	0.0268			0.5699	0.0256
90	0.5879	0.0366			0.5359	0.0302
120	0.5898	0.0265			0.5698	0.0265
150	0.5717	0.0259			0.5761	0.0272
180	0.5823	0.0276	0.5531	0.0219	0.5841	0.0322
240	0.5617	0.0305	0.5368	0.0270	0.5645	0.0334
360	0.5629	0.0334	0.5178	0.0276	0.5496	0.0290
500	0.5309	0.0313			0.5116	0.0290

This table shows the effect, on the mean accuracy, of tuning the classifier parameters. All entries in the table are for models built from the same dataset, the bag-of-words dataset that distinguishes groups 2 and 3. The table shows the mean accuracy and standard deviation for the 5-fold validation of 100 models. All models were trained so that 3000 features are pre-selected. The number of features dynamically selected during run-time are indicated in the first column. The thresholds are used to bin word-counts into 2, 3 or 4 bins by 1, 2 or 3 thresholds. When one threshold is used, it is always set at the mean word count. When two thresholds are used, they are set one standard deviation above and below the mean word count. When three thresholds are uses, they are set at the mean, and one standard deviation above and below. Histograms for the 240-feature case are shown in figure 3. It seems that using only one threshold is usually, but not always the best. The dependence on the number of dynamical features is somewhat uneven. The first three columns are graphed below.

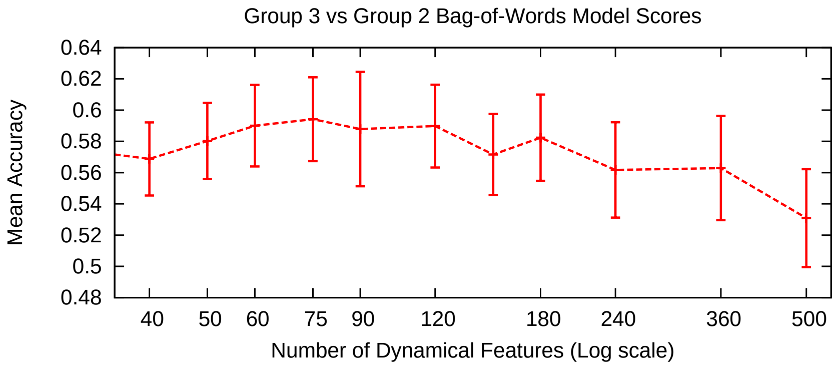

Figure 3: Parameter Variation

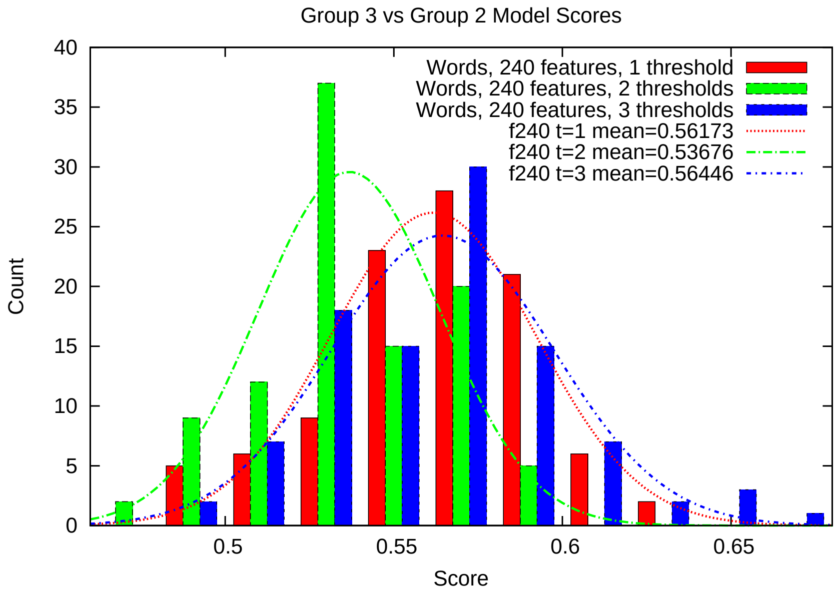

An example of accuracy score distributions for three different parameter settings. All three bar-graphs are built from the same dataset, the bag-of-words dataset that distinguishes groups 2 and 3. All three sets of models were trained so that 3000 features are pre-selected, and 240 features are dynamically selected during run-time. The only difference is whether the word-counts were binned into 2, 3 or 4 bins by 1, 2 or 3 thresholds. When one threshold is used, it is always set at the mean word count. When two thresholds are used, they are set one standard deviation above and below the mean word count. When three thresholds are uses, they are set at the mean, and one standard deviation above and below. Observe that almost all of the classification effect is derived from using just one threshold: adding two more improves classification, but only slightly. Observe that the threshold located at the mean appears to be the most important; when it is not used, classification suffers. This is not always the case; for some of the parameter settings, such as those where more or fewer dynamic-runtime features are used, the situation is reversed: an even number of thresholds work better than an odd number. Nor is it the case that adding more thresholds always improves the score; sometimes, this leads to over-training instead, as is evident in table 10.

representations. Each model is generated by using exactly the same parameters, but differing only in the initial random number seed: thus, these are true ensembles. One then asks: how many words are shared by all representations? How many are shared by most representations? How many are shared by only half of them? The answer to these questions is that two words are shared by all representations, six are shared by 90% of all representations, and 17 by more than half of all representations. Rather surprisingly, these counts do not depend much on the number N of different models: one can look at 10 or 150 models, and the number of words shared in common stays more or less the same. This is shown in figure 4, where the percentages are graphed for N=10, 40 and 150 models (100, 400 and 1500 representations).

If may be the case that many of these words are acting as if they were synonyms for one another: not in the literal sense of having the same meaning, but rather that they are being used in similar contexts. Perhaps there is a common set of words that are indicative, but some patient records only use some of them, while others use others. But perhaps, there is a different situation: when a patient record has one of these words, it also has many of the others. In such a case, it would be enough to pick just one of these words out to build a model, and if different models picked different words, its is only because they are inter-changeable, and the models are only superficially, but not deeply, different. A cluster analysis would need to be performed to determine this.

Out of the collection of all words, what is the rank of the words chosen for use in a model? This is hinted at in figure 5. As can be clearly seen, infrequently-used words are vital for distinguishing patient groups. Indeed, it would appear that distinguishing words all have fairly small counts (2 through 30 or 40), with a few exceptions. Observe that not all rare words are used for model building: there are tens of thousands of words that appear less than 5 times in the text; of these, less than a few hundred are selected for use in a model.

However, this dependence on rare words for model building indicates that the system is keying on attributes that are shared by only handfuls of patients. It is not clear if this is an artifact of the small dataset size, or whether different patients are showing distinct, non-overlapping 'symptoms'. Recall that there are only 70+69=139 patients in total that are being discriminated between by these models. Thus, if a word appears only 10 times in total in the entire text, then this word can select at most only 10 patients (unless it is a negative keyword, in which case it can be used to rule out 139-10=129 patients). Is this happening because there are 10 patients who are presenting in a very specific way? Or is this because the records are sparse, and that perhaps all patients would present in this way, but it was simply not observed and noted? In other words, do all suicidal patients present in the same way, or are there classes of distinct behavior patterns? If there is commonality to all suicidal behavior, it is not particular evident in this data.

A different measure of the importance of infrequent words can be obtained by excluding them from model building: that is, by creating models from word lists that include only those words that occur 2 or 4 or more times in the text. Superficially, this seems like a wise idea. If a word appears in only one patient record, and it is found during the training phase, then it is impossible that this word will also appear in one of the test patient records. Thus, it cannot contribute to the accuracy of the model on the test set: both the positive and negative cohorts will be missing this word; it has no

Figure 4: Common Keywords

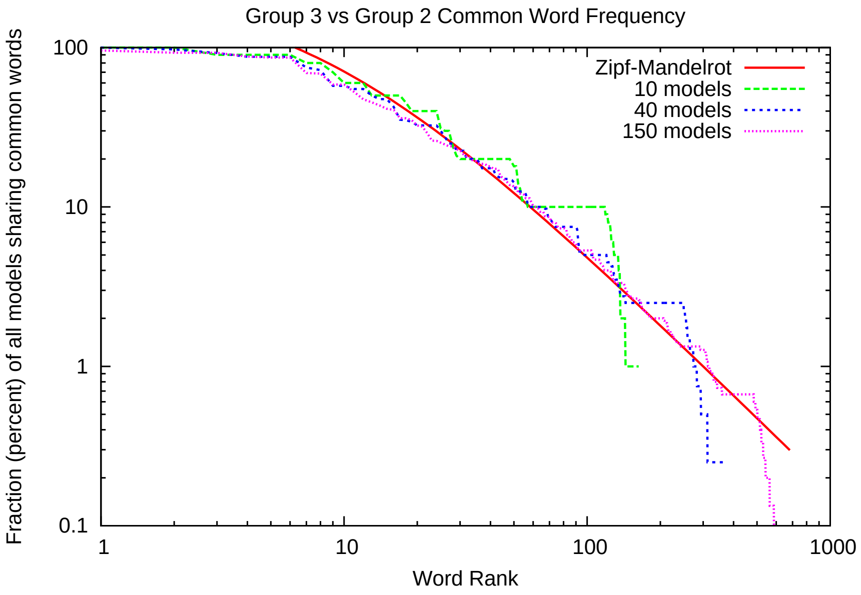

This graph shows the fraction of all representations that share words in common. Thus, the highest ranked word is used in the largest number of representations, the next highest ranked word is used in the next greatest number of representations, and so on. For the set of 10 models, there were 100 representations, which used 163 unique words among them, sharing many of them. The set of 40 models (400 reps) used 371 unique words, while the set of 150 models (1500 reps) used 682 unique words. Thus, creating more models does cause more words to be employed, but at a diminishing rate. The smooth line labeled 'Zipf Mandelbrot' is a rough fit to the data, given by the formula $\sim (\text{rank}+8)^{-1.5}$. The result here is phenomenological.

Figure 5: Model Words Rank

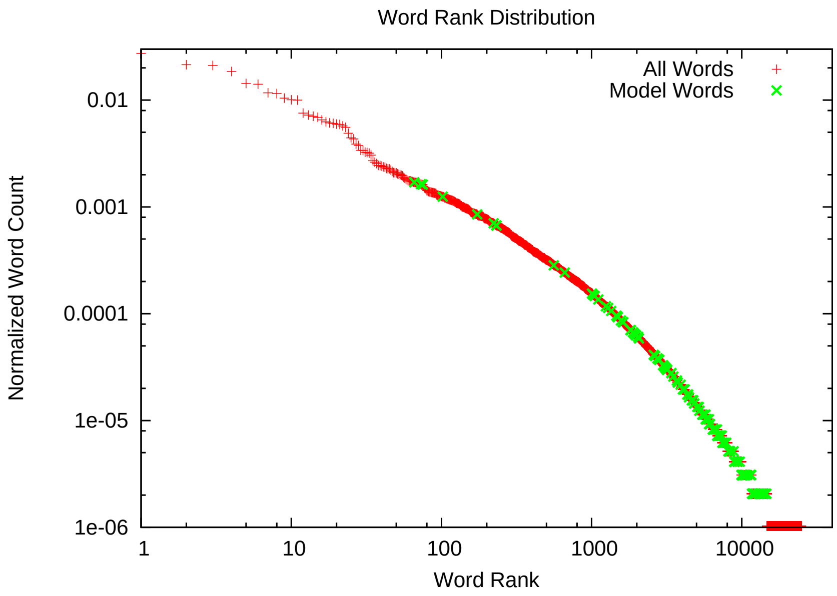

This graph reproduces that of figure 1, high-lighting the words that were used to construct models in green. In total, there are 163 words highlighted in green, taken from the N=10 model collection. It is clear that, with a few exceptions, most of the words used to distinguish patient groups are words that are infrequently used. The red bar in the lower right corresponds to words that appear only once amongst all the patient texts. The next green bar above that corresponds to words that appear twice, and so on. Thus, this graph makes clear that words that appear only a small number of times (2 through 30) are all vital for distinguishing patient groups. Note that, although the green crosses appear to dominate the lower right of the graph, this is partly an illusion: there are 163 green crosses in total, whereas there are more than ten thousands red crosses to the lower right. Thus, although words that appear only twice in the text are vital for model building, only a tiny fraction of these are actually used.

Table 11: Data Cuts

Cut	thresh=1		thresh=3	
	Mean	Std. Dev.	Mean	Std. Dev.
none	0.5571	0.0297	0.5645	0.0329
2	0.5590	0.0298	0.5578	0.0270
4	0.5406	0.0318	0.5343	0.0296
8	0.5145	0.0331	0.5153	0.0279
16	0.4996	0.0313	0.5109	0.0288

This table shows ensemble averages for the accuracy, when infrequent words are cut from the dataset. Thus, the row labeled '4' indicates results when all words appearing 4 or fewer times have been cut from the dataset. All results are, as usual, for an ensemble of 100 models. All models were trained on the same set of parameters: 3000 statically selected features, 240 dynamically selected features, and 1 or 3 thresholds, as indicated. This is the parameter choice that results in the highest score when no cuts are made, as shown in figure 2, figure 3 and table 10. Cutting words that appear only once is the same as cutting none at all.

---

predictive value. If a word appears in only two patient records, then it is unlikely that one of those locations will be in the test-set (due to the 4/5'ths - 1/5'th split). Thus, one might also believe that such words have little or no predictive value. Perhaps accuracy can be increased by cutting down the dataset, and discarding all words that appear fewer than M times in the dataset. But this is very much not the case. Results are shown in table 11.

Cutting rare words decreases model accuracy. A modest cut of even 4 words has a large impact on scores, and cutting more than that essentially wipes out the predictive accuracy of a model almost completely.

Word Pairs and N-grams

A common issue that arises when machine learning is applied to sentiment analysis is that positive and negative keywords can be negated in the text, inverting their meaning. So, for example, a keyword "unhappy" may occur in a sentence "he is not unhappy." Another issue is that semantic meaning is not confined to single words, but can be associated with word pairs, collocations (set phrases) and idioms. Looking at merely one word of a collocation may imply a less refined meaning ("intramuscularly" is not specific the way that "left deltoid intramuscularly" is) or possibly a completely different meaning altogether ("disturbances" has psychological overtones; "visual disturbances" suggest something completely different: glaucoma or macular degeneration. Similarly: "alcohol" vs. "alcohol screening test", "pain" vs. "pain relieving", *etc.*) Thus, one might expect greater predictive value arising from using neighboring word pairs or even perhaps entire phrases[7]. This is indeed the case, as demonstrated in this section.

In order for this technique to work, one must be careful to apply appropriate cuts to

the dataset. Simply including all possible word pairs does not improve model accuracy. The reason for this is well-known: by including word-pairs, the number of candidate features that might fit the data enlarges to a much larger number. Statistical chance means that some of these may correlate strongly with the training set, even though they are not actually predictive. Discarding word pairs with a low mutual information (MI) score is an obvious cut to make; one may also contemplate discarding infrequent word pairs, although experience with single words suggests that this is not a good idea.

An alternative to discarding word pairs with low MI is to consider only those word pairs that involve a word that has previously been identified as being 'predictively significant', that is, a word that already occurs in a single-word model, such as those in tables 7 and 8. Word pairs constructed from these words are 'clinically interesting', in that they provide a larger window into the notes occurring in a patient record. In this sense, the approach is inspired by the central idea of corpus linguistics: in order to better understand the meaning of a word, it is best to view it in context, to see how it is being used. Inspired by this idea, it is reasonable to contemplate using three-word phrases (trigrams) and 4-word phrases (4-grams) to construct a bag-of-phrases. In what follows, these n-grams will be referred to as 'corpus n-grams', indicating that they were constructed from 'clinically interesting' words. This is to draw a distinction between these, and the set of all n-grams cut down by MI scores. This sort of an approach is known to provide a positive benefit for classification[2].

To create the list of 'significant words', an ensemble of 40 models were trained on the group 3 vs. group 2 dataset. As noted previously, in the caption to figure 4, this ensemble results in 371 unique words. The set of corpus n-grams were then selected by considering only those n-grams that contained one of these 371 words.

In what follows, n-grams are constructed not only from n adjacent words, but also from adjacent words with 'holes' (wild-cards) in them. The reason for doing this is to properly take into account multi-word noun and verb modifiers. Thus, for example, the phrase "horrible frightening experience" is composed to two semantically interesting units: "horrible_experience" and "frightening_experience" (this example is taken from the actual dataset) . The first would not be captured if one limited oneself solely to adjacent words when creating pairs. Likewise, when constructing 3-grams, not only were three adjacent words considered, but also all possibilities for picking three words out of a string of four consecutive words. When creating 4-grams, all possibilities for picking 4 words out of 5 consecutive words were considered. When a bag-of-n-grams is constructed, it also includes those n-grams that are shorter: thus, the bag-of-pairs also includes single words, and the bag-of-trigrams also includes pairs and single words in it. Thus, during model building, a pair or a trigram is used only if it results in a better model than using some individual word.

This idea of allowing holes in n-gram construction is to partly emulate the action of a syntactic parser, which would be able to identify meaningful semantic relationships, such as adjective-noun or even subject-verb. In place of syntactic parsing, high mutual-information phrases can help identify meaningful phrases, and in some ways, can even be superior, given the fractured, badly-structured and non-grammatical content of the notes. Unfortunately, even this approach is insufficient to deal with long-range correlations between words in the text. The example given above occurs in a note as a part of a semi-automated system for PTSD screening, whose full content, as it appears in

Figure 6: N-grams

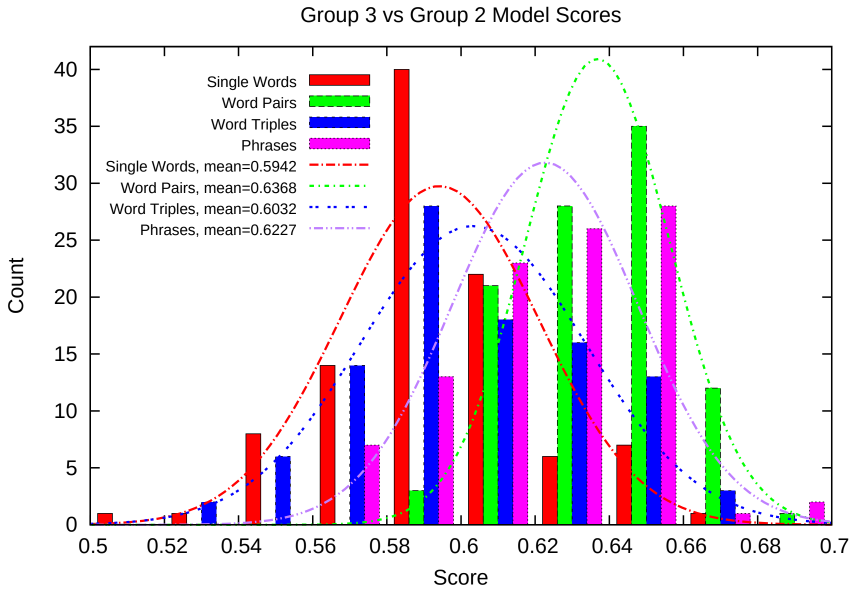

This bar chart compares three different bag-of-phrases models to the highest performing bag-of-words model. Observe that all of the bag-of-phrases models outperform the best bag-of-words model. Results of parameter tuning are shown in table 12.

---

the note, is: "*Have you ever had any experience that was so frightening, horrible, or upsetting that, IN THE PAST MONTH you: Have you had any nightmares about it or thought about it when you did not want to? NO*". This note appears for a control-group patient; the presence of such semi-automatically generated notes adds to the classification challenge. It is not clear how to extract this kind of information; the challenge is similar in some ways to that of anaphora resolution (the word '*NO*' being the resolution to the question) , and perhaps techniques from that area could be applied.

The corpus-linguistics-inspired approach, of considering only those word phrases that contain words that were previously identified as 'significant', works very well. This is illustrated in figure 6, where the results of four different experiments are shown: the best bag-of-words result, the best corpus-pairs result, the best corpus-trigram result and the best corpus 4-gram result. Particularly noteworthy is that all of the bag-of-phrases models perform better than the best bag-of-words model. Of all these, the most outstanding are the word-pairs results.

Creating the set of corpus-pairs requires having previously computed a list of 'significant words'. Creating that list is time-consuming, since it requires training an ensemble, extracting the words, and then training again, with pairs. Thus, it is natural to ask if there are simpler ways of obtaining a list of 'significant words' that are just as good. There are: in fact, simple single-word feature selection is sufficient to create a

Table 12: Bag of Phrases Tuning Results

Num. Dyn. Feat	single words	word pairs	trigrams	4-grams
2	0.5301	0.5794	0.5642	0.5537
3	0.5112	0.5983	0.5473	0.5589
5	0.5168	0.6283	0.5649	0.5635
8	0.5305	0.6069	0.5440	0.5442
12	0.5065	0.5981	0.5551	0.5947
16	0.5450	0.5823	0.5614	0.6227
24	0.5284	0.5691	0.5615	0.5532
32	0.5735	0.5917	0.5546	0.6020
40	0.5688			
50	0.5803	0.5914	0.6032	0.5981
60	0.5901	0.5868		
75	0.5942	0.5899	0.5887	0.5801
90	0.5879	0.6227	0.5721	0.6019
105		0.5769		
120	0.5898	0.5772	0.5817	0.5706
150	0.5717	0.5884		
180	0.5823	0.5906	0.5727	0.5792
240	0.5617	0.6036	0.5746	0.5773
300		0.6028		
360	0.5629		0.5891	0.5784
400		0.5911		
500	0.5309	0.5715	0.5816	0.5655

This table shows ensemble averages for the accuracy as the number of run-time dynamic features was varied. As usual, the ensemble consists of 100 models, made from 3000 pre-selected features; only one threshold is used. Single-word results are identical to those reported in table 10. The best ensembles from each column are graphed in figure 6. The datasets were subjected to cuts: All words and n-grams that occurred 4 or fewer times were cut, as were all n-grams with an MI of less than 2. The chart below graphs the table contents. Bars showing the standard deviation are plotted only for the single-word scores; those for phrases are comparable or smaller.

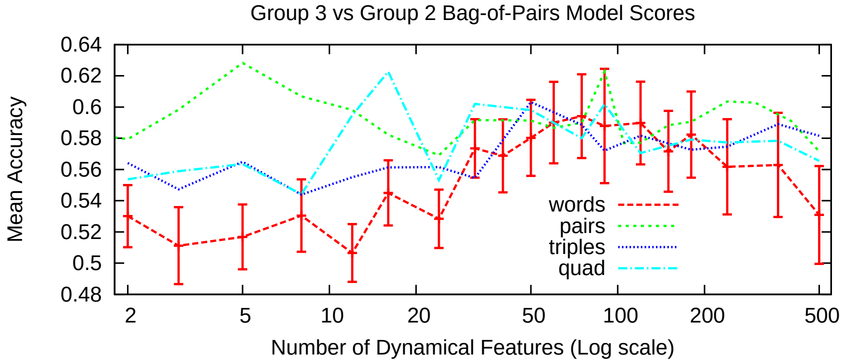

Table 13: Choosing Significant Words

Word Source	Mean Accuracy	Std. Dev.
Ensemble	0.5986	0.0324
Feature Selection	0.6036	0.0295

A comparison of word-pair ensemble accuracy results for sets of corpus word-pairs created from two different sources of 'significant words'. The ensemble 'significant words' consist of 371 words taken from an ensemble of 40 models. The feature-selection 'significant words' consist of 371 words selected by maximizing mutual information between the word counts and the cohort id. The ensembles were trained with the same parameters as reported above: 3000 static features, 240 dynamic features, 1 threshold, on the group 3 vs. group 2 dataset.

---

list of 'significant words' that is every bit as good as that obtained from the ensemble, and maybe even a little bit better, as shown in table 13. To make the two methods comparable, a simple mutual-information-maximizing feature selection step was performed to select 371 words, the same number of words as obtained from the ensemble. Feature selection runs in seconds, whereas training an ensemble of 40 models can take hours.

The distribution of corpus-pairs vs. all-pairs is dramatically different, as shown in figure 7. By selecting corpus pairs, thousands of the highest-MI pairs are discarded, as well as most of the low-MI pairs, as well. Perhaps it is possible to replicate the corpus-pairs results by applying a simple cut to the all-pairs dataset, and merely discarding the low-MI pairs? This does not seem to be the case, as shown in table 14. This table compares a bag-of-words model to several different all-pairs models, with different MI cuts applied. Including all pairs does not improve the score over the bag-of-words. By cutting out low-MI pairs, the score can be improved somewhat, but the effect is not dramatic; certainly not as strong as the decision to use corpus-pairs.

The original bag-of-phrases results shown in tables 12, 13 and figure 6 were made by employing some arbitrary, 'intuitive' cuts for the number of words, and for mutual information. Later experiments on the effect of cutting rare words shows a net negative effect, as documented in table 11. Perhaps it is a mistake, then to cut rare words and rare word-pairs, when using a bag-of-pairs model? It doesn't seem so: table 15 shows a counter-intuitive result. In this experiment, no rare words or pairs were cut; only the cut for the MI was altered. None of the results approach the best accuracy from table 13. Thus, somehow, when word-pairs come into play, failing to cut rare words and phrases makes things worse!

To improve scores, are the relevant cuts to the rare words, to the rare phrases, or both? The answer is both, as revealed in table 16. Of the two, cutting infrequent words seems to provide the greater benefit.

Figure 7: Distribution of Mutual Information

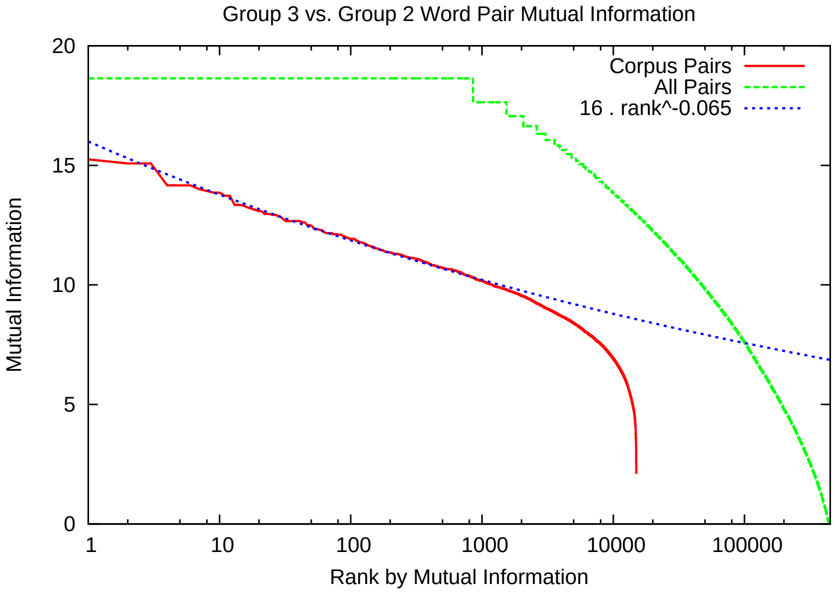

The distribution of corpus-pairs and all word pairs, ranked in decreasing order of mutual information. The distributions are rather dramatically different; the corpus-pairs distribution having a Zipfian segment which is lacking in the all-pairs distribution. The relative rank of a pair can be obtained by drawing a horizontal line across the two curves: the corpus-pairs set eliminated all of the high-mutual-information pairs, as well as most of the low-mutual information pairs.

Table 14: All-Word-Pair Cuts

	Mean Accuracy	Std. Dev.
Single Words	0.5617	0.0305
All Pairs	0.5602	0.0418
All Pairs MI>0	0.5764	0.0275
All Pairs MI>2	0.5646	0.0282
All Pairs MI>4	0.5751	0.0277
All Pairs MI>6	0.5655	0.0281

Ensemble averages for accuracy, comparing different cuts to the bag-of-single-words accuracy. Simply including all word pairs does not improve the quality of the model. Discarding those pairs with low mutual information scores does, however, have a net positive effect. The best location for the MI cut is not entirely clear, though. All ensembles were trained with 3000 pre-selected features, 240 dynamically selected features, and one count threshold. (Note that these training parameters do not result in either the best-words or the best-pairs models, and so should not be directly compared to those results).

Table 15: Mutual Information Cuts

	Mean Accuracy	Std. Dev
Best Pairs	0.6036	0.0295
Pairs MI>0	0.5518	0.0294
Pairs MI>2	0.5871	0.0289
Pairs MI>4	0.5394	0.0250
Pairs MI>6	0.5340	0.0303

A study of the effect of varying the mutual information cut for corpus pairs. All models were trained on the same parameters (3000 static features, 240 dynamic features, 1 threshold). The four entries labeled “Pairs MI>x” do not have any cuts for rare words or rare pairs, and show the results of different MI cuts. The entry labeled “Best Pairs” reproduces that from table 13: namely, having three cuts: besides MI>2, it also cuts words that appear 4 or fewer times, and cuts phrases that appear 4 or fewer times.

Table 16: Cutting Phrases

Word Count Cut	Pair Count Cut	Mean Accuracy	Std. Dev
4	4	0.6036	0.0295
0	4	0.5871	0.0311
0	2	0.5814	0.0351
2	2	0.5830	0.0300
2	0	0.5908	0.0297
4	0	0.5901	0.0309
0	0	0.5871	0.0289
6	6	0.5711	0.0309

A comparison of different word and phrase cuts on the accuracy. All models are built on the corpus-pairs dataset, and reject pairs with an MI of 2 or less, and were trained on the same parameters (3000 static features, 240 dynamic features, 1 threshold). The highest scoring ensemble also rejects words and word-pairs that occur 4 or fewer times. The other entries explore the effect of cutting fewer or more words and/or pairs; none do particularly well, not quite approaching the best result.

Static vs. Dynamic Feature Selection

The use of dynamic feature selection also has a strong effect on both training times (by reducing the size of the problem) as well as on the quality of the fit. Table 17 shows the effect of dynamic feature selection on the overall score.

Evaluation Times

MOSES is not immune to the effect of over-training: longer training times result in a better fit to the train dataset, but the resulting models perform more poorly on the test dataset. In essence, longer training times allow the system to find quirks in the training set that are not present in the test set. Once a minimum amount of training has been done, any correlation between train and test scores disappears; there is even a vague hint of anti-correlation as shown in figure 8.

Optimum training times are explored in table 18. Training times are measured in terms of the number of evaluations of the scoring function: a single comparison of the model to the training table counts as one evaluation. The highest score for each row is marked in bold (magenta); the second-highest score in italic (cyan). The very highest score, over the entire table, of 63.68%, occurs at a training time of 5000 evaluations and 90 dynamical features (indicated in bold sans-serif). Most of the high scores occur when 9000 or fewer training evaluations are performed. The exceptions occur when the number of dynamical features is extremely small: this suggests that the model builder is starved for features at this point, and must iterate over many trials before finding the appropriate features.

Table 17: Static vs. Dynamic Feature Selection

Num. Feat.	Static	Dynamic
2		0.5794
3		0.5983
5		0.6283
8		0.6069
12		0.5981
16		0.5823
24	0.5553	0.5691
32	0.5580	0.5917
50	0.5628	0.5914
60		0.5868
75	0.5960	0.5899
90	0.5958	0.6227
105		0.5769
120	0.5764	0.5772
150		0.5884
180	0.5619	0.5906
240	0.5526	0.6036
300		0.6028
360	0.5436	
400		0.5911
500	0.5501	0.5715

This table shows ensemble averages for the accuracy comparing static and dynamic feature selection. As usual, the ensemble consists of 100 models; one threshold was used. The dynamic feature-selection results are identical to those reported in table 12; they are obtained by statically selecting 3000 features, and then dynamically selecting the indicated number from those. By contrast, the static selection process just chooses the indicated number of features initially, and makes no dynamic selection at all.

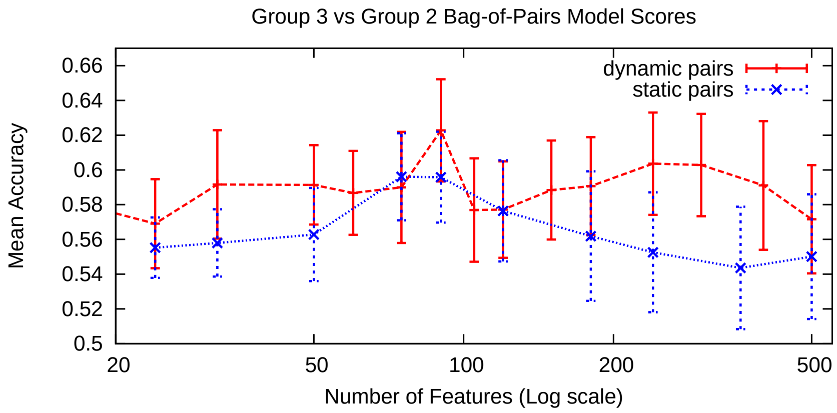

Table 18: Training Time Dependence

Dyn. Feat	3K Eval	5K Eval	9K Eval	15K Eval	27K Eval	45K Eval	70K Eval	110K Eval	160K Eval	320K Eval
3	0.5914	0.5965	0.5967	0.5955	0.5922	0.5937	0.5959	*0.5973*	**0.5983**	0.5965
5	0.5959	0.6192	0.6271	*0.6288*	0.6281	0.6268	0.6265	0.6281	0.6283	**0.6301**
8	*0.6185*	**0.6229**	0.6140	0.6122	0.6099	0.6066	0.6062	0.6068	0.6069	0.6055
12	0.5871	0.6049	*0.6088*	**0.6091**	0.6055	0.6022	0.6002	0.5995	0.5981	0.5942
16	0.5563	0.5796	**0.5957**	*0.5932*	0.5925	0.5886	0.5855	0.5847	0.5823	
24	0.5678	0.5678	**0.5765**	0.5671	0.5681	0.5682	0.5684	*0.5692*	0.5691	0.5681
32	**0.5965**	0.5720	0.5732	0.5923	*0.5945*	0.5929	0.5929	0.5927	0.5917	0.5918
40	0.6214	*0.6240*	**0.6288**	0.6221	0.6141	0.6139	0.6140	0.6136	0.6132	
50	**0.6273**	*0.6145*	0.5924	0.5971	0.5935	0.5924	0.5927	0.5919	0.5914	0.5899
60	*0.6244*	**0.6277**	0.6060	0.5876	0.5868	0.5872	0.5868	0.5871	0.5868	
75	*0.6240*	**0.6245**	0.5982	0.5921	0.5893	0.5894	0.5895	0.5902	0.5899	0.5894
90	0.6143	**0.6368**	0.6204	0.6201	0.6227	*0.6231*	0.6224	0.6222	0.6227	0.6212
105	0.5813	**0.6004**	*0.5863*	0.5750	0.5768	0.5765	0.5768	0.5765	0.5769	
120	*0.6055*	**0.6112**	0.6045	0.5799	0.5778	0.5781	0.5783	0.5776	0.5772	0.5768
150	0.5878	*0.6168*	**0.6200**	0.5951	0.5897	0.5904	0.5893	0.5886	0.5884	
180	0.5839	*0.6063*	**0.6070**	0.6006	0.5919	0.5913	0.5908	0.5901	0.5906	0.5898
240	0.5891	0.6040	**0.6145**	*0.6095*	0.6028	0.6040	0.6037	0.6035	0.6036	0.6024
300	0.5874	0.5890	**0.6170**	*0.6067*	0.6045	0.6033	0.6029	0.6029	0.6028	0.6027
400									0.5911	
500									0.5715	

Figure 8: Train-Test Score Correlation

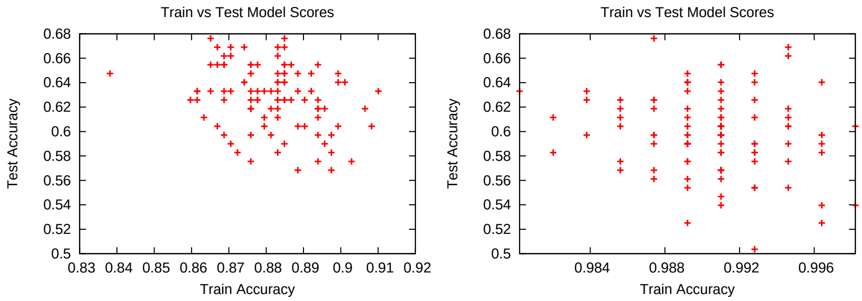

The above two scatter-plots show the test vs. train scores for 100 models. As should be clear, there is essentially no correlation: a model that scored well on the training set may or may not do poorly on the test set, and *vice versa*. The left figure is for 5 dynamical features, the right figure is for 320. As is clear from the x-axis labels, using more dynamical features helps improve the training score by a lot; however, the test-score degrades, as noted in table 12.

Voting

The concept of the ensemble replaces a significant random variation in the accuracy of a single representation with a more trustworthy average accuracy across multiple representations. This does not imply that the accuracy of the ensemble model is equal to the average accuracy of the representations in the ensemble. When multiple representations are allowed to vote for a final classification, the accuracy of the classifier usually increases[5]. This section explores how the accuracy of a model depends on the number of representations voting in the model.

The results here are reported in the same fashion as before, except that now, each model contains N representations, instead of just one representation. In essence, there is now an ensemble of ensembles: although a model may consist of N representations, we still explore the average accuracy taken over 100 models. The raw data are presented in 19 and graphed in 9. Typical cross-sections are shown in 10.

Additional insight can be gained by examining how the representations voted for individual patients. This is shown in figure 11. Any given patient can receive anywhere from 0% to 100% of the votes. A vote 'for' indicates the patient belongs to group 3, a vote 'against' indicates that the patient belongs to group 2. Thus, those receiving less than 50% of the vote are classified as group 2; those receiving more are classified as group 3. The graph then shows the fraction of votes received, versus the known a priori patient cohort membership. Ideally, a 100% accurate classifier would always give more than 50% of the votes to group 3 members, and always less than 50% of the votes to group 2 members. The fact that the classifier is sometimes wrong is readily apparent in the graph.

A notable feature of the graph is that it is not symmetrical: that is, the red bars

Table 19: Voting Ensemble Averages

Model Size	Mean	Std. Dev.
1	0.6425	0.0302
3	0.6520	0.0257
7	0.6563	0.0209
11	0.6566	0.0154
15	0.6576	0.0156
21	0.6614	0.0131
31	0.6630	0.0117
41	0.6633	0.0089
55	0.6653	0.0104
81	0.6690	0.0086
101	0.6678	0.0096

Voting ensemble results. Each model consists of N='Model Size' representations, with a majority vote determining how the model classifies. The mean and standard deviation are obtained by averaging over 100 different models, built by varying the initial random number seed of the machine learning system. Note that the standard deviation of the N=1 model is comparable to that of 2; as N increases, the score improves, and the variation shrinks sharply.

All models were word-pair models, with the usual word-pair cuts (mi>2, and all single-words and word pairs that appear less than 4 times discarded). Word pairs were considered only if they one of the two words were one of the top 750 most score-correlated single words (this differs from the other reported word-pair results, where 371 words were used to create pairs; thus scores are not immediately comparable). The run-time dynamical feature count was set to 90, and a total of 9000 training evaluations were performed. These parameters are more or less identical to those discussed for much of this paper, and, for the N=1 case, correspond to the highest score seen.

This table is graphed in figure 9.

Figure 9: Voting Ensemble

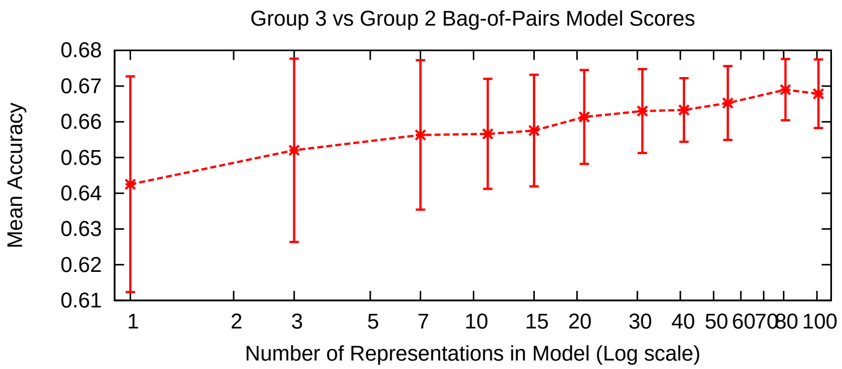

Above is a graph of the voting model accuracy for the va32 dataset (750 significant features, -m=9000 training time). Raw data taken from table 19

Far left is the best result for models containing a single representation: *i.e.* poses was trained 100 times, on the same parameters, varying only the initial random seed. The average accuracy was 64.25% The error bars show the variation among these 100 models: some scored as high as 69%, some were down in the mid-upper 50's.

The next point over shows the results for a model containing N=3 representations. That is, 3 different random seeds are used to create 3 representations. These are placed in a model, and these then 'vote' for the most likely classification (the cohort that gets 2 or more votes 'wins'). This process is repeated 100 times (same parameters, thus 300 different random seeds). The average accuracy of 100 N=3 models is 65.63% The error bars again show the variation among these 100 models: the best scoring model hit 69%, the worst-scoring had a low of 61%

The rightmost point is for a model holding 101 representations. The average accuracy (of 100, etc.) is 66.53% the best scoring is 69%. The worst-scoring is 63%. Notice that the best scores are always pegged at 69% The ensemble seems to merely trim away the outliers with the bad scores.

Figure 10: Distribution of Voting Models

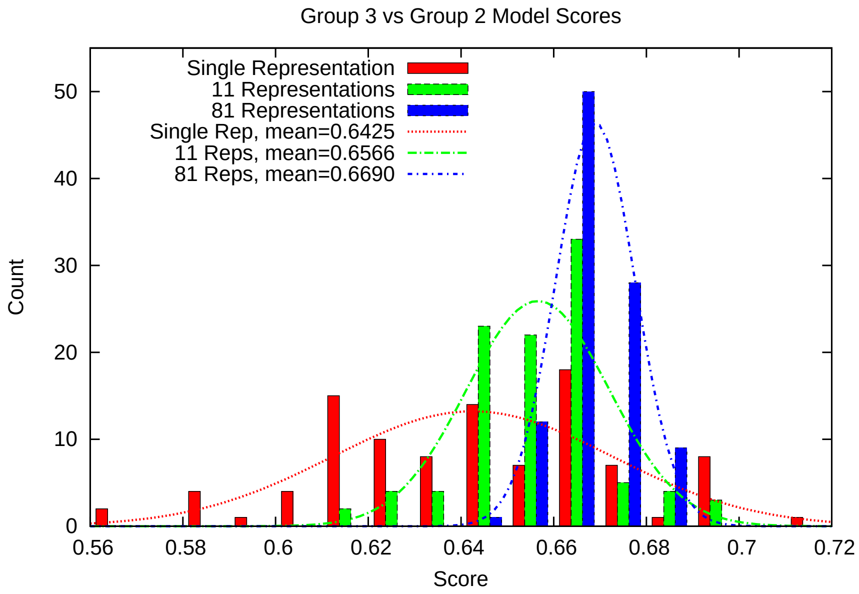

This figure demonstrates typical distributions taken from figure 9; the means and widths of the Gaussians shown here are exactly those of table 19.

Figure 11: Voting Blocks

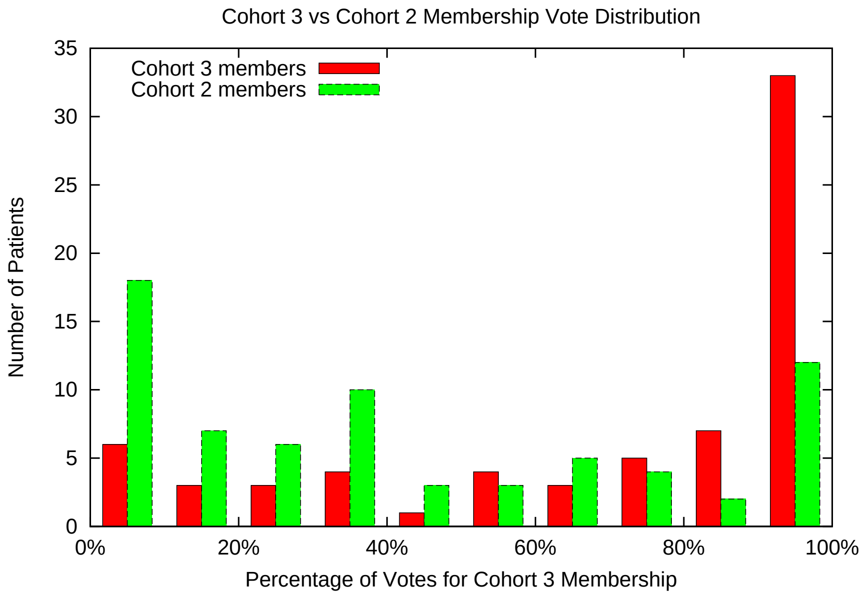

This figure shows a model comprising N=101 representations, voted to classify individual patients. Vote counts were divided into 10 bins; the number of patients receiving that number of votes is shown. Thus, the tall green bar on the far left indicates that 18 patients from group 2 received 10% or fewer votes; these patients are correctly classified by the voting. By contrast, the red bar on the far left indicates that 6 patients from group 3 received 10% or fewer votes; these patients are misclassified by voting. Indeed, all red bars to the left of the 50% mark, and all green bars to the right of the 50% mark indicate misclassified patients.

Just as in the rest of this paper, this shows the performance of the classifier on the test set, using 5-fold validation. Different models, created with different random seeds, show a very nearly identical vote distribution.

are not a mirror image of the green bars. Of particular interest is that the classifier is overall quite confident in its classification of group 3 patients (this is the psychiatric group); this can be seen in the tall bar on the right-hand side of the graph. That is, given a patient from group 3, the classifier can correctly classify the patient with good accuracy and high confidence. This is not at all the case from group 2, the suicide cohort: here, the classifier is clearly less accurate, and more tentative in its assignment. This can be seen in that the left-most green bar is not that tall, and that the rightmost green bar is not very small, as one might have hoped. In essence, the classifier is good at recognizing the psychiatric patients; but the suicidal patients, not so much.

Conclusion

Training classifiers to distinguish the three groups of patients is a straightforward task. Given the relatively small dataset size, it was also easy to train these classifiers to be "over-fit": to perform very well on the training set, sometimes achieving a perfect score, but scoring rather poorly on the test set. Accuracies up to 67% were obtained for ensemble averages of 100 models, trained on the best parameter choices, with individual model accuracies rising as high as 69%.

Finding the best models is an arduous task. To evaluate an ensemble of 100 models with 5-fold cross-validation requires a total of 500 models to be trained; this can take days of wall-clock time, as individual models require anywhere from a few minutes to a decent fraction of an hour to train. In order to obtain a good fit, several training parameters must be explored: the thresholding of word-counts into bins, and the run-time dynamical feature-selection size. These parameters must be tuned individually for different data sets; they are adjusted to best bring a view of the dataset into sharp focus.

The most interesting result is that word-pairs can be used to build more accurate models than single words alone. However, in order for this to work well, a number of data cuts must be applied: word pairs with low mutual information scores should be discarded; infrequently occurring pairs and words should be discarded, and, most important of all, word-pairs that don't contain 'significant' words should be discarded as well.

References

[1] Cindy Chung and James Pennebaker. The psychological functions of function words. In K. Fiedler, editor, *Social communication: Frontiers of social psychology*, pages 343–359. Psychology Press, 2007.

[2] Ben Goertzel and J. Venuto. Accurate svm text classification for highly skewed data using threshold tuning and query-expansion-based feature selection. In *Neural Networks, 2006. IJCNN '06. International Joint Conference on*, pages 1220–1225, 2006.

[3] Moshe Looks. *Competent Program Evolution*. PhD thesis, Washington University St. Louis, 2006.

[4] Moshe Looks. Meta-optimizing semantic evolutionary search. In Hod Lipson, editor, *Genetic and Evolutionary Computation Conference, GECCO 2007, Proceedings, London, England, UK, July 7-11, 2007*, page 626. ACM, 2007.

[5] D. Opitz and R. Maclin. Popular ensemble methods: An empirical study. *Journal of Artificial Intelligence Research*, 11:169–198, 1999.

[6] Shannon Wiltsey Stirman and James W. Pennebaker. Word use in the poetry of suicidal and nonsuicidal poets. *Psychosomatic Medicine*, 63(6):517–522, 2001.

[7] Hanna M. Wallach. Topic modeling: Beyond bag-of-words. In Andrew Moore William Cohen, editor, *Machine Learning ICML/2006, Proceedings of the 23rd International Conference on*. ACM, 2006.
