# Supplementary material for: Predicting the Risk of Suicide by Analyzing the Text of Clinical Notes
Source: PLoS One. 2014 Jan 28;9(1):e85733. doi: 10.1371/journal.pone.0085733 (PMC3904866; doi:10.1371/journal.pone.0085733)
Supplement: Appendix S3 — Machine Learning Libraries and Methods: This section is provided to enclose the open source (Apache License) classifier used for the building of the statistical models, specifically for the purposes of study reproducibility. A detailed account of these tools is intended for another publication. (ZIP) [file pone.0085733.s003.zip › Appendix3/Data_Analysis_HOWTO.pdf]

DCAPS Suicide Data Analysis: Mini-HOWTO

February 15 2013

Linas Vepstas linasvepstas@gmail.com & Chris Poulin chris@patternsandpredictions.net

This mini-HOWTO briefly reviews how to use the POSES machine learning package to perform data analysis for the DCAPS suicide data. This includes *training* of, *validation* of, and *inference* on the model.

Description of the data

The Veterans Administration data is available in a pair of files, named "va12-pairs-allowed.csv" and "va32-pairs-allowed-asimple.csv". These files were obtained from patient medical records in cooperation with the Dartmouth-Hitchcock Medical Center (DHMC). These files are in the style of a "bag-of-phrases", containing word and word-pair counts derived from free-text medical records. The original medical records are confidential, and cannot be moved/copied outside of DHMC. The bag-of-phrases data is slightly less sensitive, but still confidential.

The study contains records for three groups of patients: groups 1, 2 and 3, corresponding to the control, suicide and psych groups, respectively. There are 70, 69 and 70 patient records in each group. The data files contain one row per patient; that row contains the word-counts for the medical records for that patient. Each row is labeled with the group that the patient belongs to; that label is in the first column. The file "va12-pairs-allowed.csv" contains data for groups 1 and 2 only, and thus has a total of 70+69=139 records total. The file "va32-pairs-allowed-asimple.csv" contains data for groups 3 and 2 only, and again consists of 139 records total. In this later file, the group 3 records are labeled as belonging to group 0 (instead of 3); this re-labeling is done so that the psych group can be handled as "negatives" (non-suicides) as opposed to "positives".

Using the POSES machine learning tool

The POSES Machine learning tool and its use is documented in three different files and directories, included with the POSES binary and source distributions. These include the "README.txt" file, which provides a general overview, install instructions, and some basic examples. The "man/poses.1" file provides a standard Unix man page, documenting the command-line usage and flags. The "example-bank" directory contains a tutorial and example datasets based on a banking customer satisfaction survey. The remainder of this HOWTO presumes a thorough familiarity of the POSES system. The man page should be consulted to understand the meaning and operation of each command line flag.

Training

Training for the group-3 vs. group-2 discriminator can be performed as follows:

```
poses moses -d va-grp-32.csv -m model-32.mod -p group -e160000 -n2 -s1 -F"-C3000 -asimple
-j4" -M"-j4 --hc-max-nn-evals=5000 --enable-fs=1 --fs-target-size=90 -B0" --restarts=10
```

This instructs poses to:
*) use the va-grp-32.csv file, (the -d option)
*) output the final model to a file called "model-32.mod" (-m option)
*) use the first data column as the target (-p group option)
*) Perform no more than 160K training evaluations (-e option)
*) Generate only two outputs (negative/positive) (-n option)
*) Threshold the input data with one threshold (-s option)
*) Pre-select 3000 input features (the -F option)
*) Dynamically select 90 input features (--fs-target-size)
*) Build an ensemble of ten models (the --restarts option)
*) Assume a quad-core CPU (-j4)

Note that, with the above settings, training may take several hours or longer on a large, capable multi-core processor. The precise training time will vary, depending on how the various options and values described above are set.

The above was tested on a 4-core Intel i3-2105 and took approximately an hour to complete. Maximum memory usage was approximately 600MB.

The model files delivered to Raytheon BBN used an ensemble of 100 models, and not 10 as shown above. Warning: Building such models will take roughly ten times longer.

K-Fold Cross-Validation

Five-fold cross-validation for the group-3 vs. group-2 dataset takes a very similar form, as follows:

poses accuracy -d va-grp-32.csv -m model-32.mod -p group -e160000 -n2 -s1 -F"-C3000 -asimple -j4" -M"-j4 --hc-max-nn-evals=5000 --enable-fs=1 --fs-target-size=90 -B0" -f5

Notice that all of the flags are the same, except that the "accuracy" indicates that cross-validation should be performed, and that -f5 indicates that the cross-validation should be 5-fold.

Note that five-fold cross-validation repeats the training step five times, thus, cross-validation will typically run five times longer than training a single model. Note that in the previous example above, ten models are trained; thus, this particular example will only take half the time.

ROC curves

In order to create ROC curves, multiple commands such as the following may be used:

poses accuracy -d va-grp-32.csv -m model-32.mod -p group -e160000 -n2 -s1 -F"-C3000 -asimple -j4 -Hprerec -Q2.0 -A1.0 -q0.65" -M"-j4 --hc-max-nn-evals=5000 --enable-fs=1 --fs-target-size=90 -B0" -f5

The primary difference between this and the previous command are a set of Moses (the genetic programming library dependency) options that tell it to use a different scorer. The default scorer attempts to maximize accuracy (that is, to minimize the overall total of false-positives and false-negatives); this, however, is not suitable for generating ROC curves, where non-minimal false-negatives are not only desired, but are fundamental to the definition of an ROC curve.

The scorer used here is the "prerec" scorer (-Hprerec option). This scorer attempts to maximize the precision, while holding the recall above a minimum desired value. In the above example, the minimum requested recall is 0.65 (the -q0.65 option). The definition of precision and recall used here is industry standard; see for example Wikipedia for details. The two additional moses options, -Q2.0 and -A1.0, set the recall maximum bound to 1.0 (-A1.0 option) and set the "strictness" or "hardness" of the scorer in respecting the minimum desired recall.

In order to generate an ROC curve, one must re-run each cross-validation once, for each nominal recall value. Since each cross-validation may take 2 to 12 hours wall-clock time, generating an ROC curve with ten points may take 20 to 120 hours wall-clock time. Note that training for low recall values can be extremely quick: typically, a minute or less for recall rates of less than 0.5 or so. Thus, a cross-validation run for -q0.65 may take 5-15 minutes, one for -q0.75 may take an hour, while those for -q0.95 may take many hours or even a day.

<u>Typical output</u>

Below follows some typical output. It was obtained with exactly this command:

poses accuracy -d va-grp-32.csv -m model-32.mod -p group -e40000 -n2 -s1 -f5 -F"-C3000 -asimple -j4" -M"-j4 --hc-max-nn-evals=5000 --enable-fs=1 --fs-target-size=90 -B0" -V

This command took only a few minutes to run. It ran relatively quickly because the total number of scoring function evaluations was cut back to 40 thousand from 160 thousand. Cutting back on the number of evaluations can result in a less accurate model.

The output generated was:

```
Train matrix:
Classifier results in columns:
0.496402877698  1.4964028777
Expected results in rows.
275   5
1     275
```

```
Accuracy : 0.989208633094 (550 correct out of 556 total)
Precision: 0.982142857143 (275 correct out of 280.0 total)
Recall   : 0.996376811594 (275 correct out of 276.0 total)
FP Rate  : 0.0178571428571 (5 false pos out of 280.0 total)
F_1 Score: 0.989208633094
F_2 Score: 0.993497109827
----
Test matrix:
Classifier results in columns:
0.496402877698  1.4964028777
Expected results in rows.
43      27
22      47
Accuracy : 0.647482014388 (90 correct out of 139 total)
Precision: 0.635135135135 (47 correct out of 74.0 total)
Recall   : 0.68115942029 (47 correct out of 69.0 total)
FP Rate  : 0.385714285714 (27 false pos out of 70.0 total)
F_1 Score: 0.657342657343
F_2 Score: 0.671428571429
---------------------------------------------------------------------
```

(The first confusion matrix reports the results of the classifier on the training set. The second reports the results of the classifier on the test set.)

Inference

Given a test case document, we can then provide the ability to conduct a single inference. The reported inference is currently an output of a linear number range of 0-2.

In the case of a Group 3vs2 classifier, a score of 0.4964028776981 would indicate the psychiatric cohort, while 1.4964028777 would indicate suicidaility cohort classification. While in the case of a Group 1v2 classifier, a score of 1.24820143885 would indicate the non-psychiatric cohort, while 1.74820143885 would indicate suicidaility cohort classification. The two command examples;

poses inference -m model-32.mod -p group -c testcase.dat |tee inference.out
1.4964028777 (suicidaility)
poses inference -m model-12.mod -p group -c testcase.dat |tee inference.out
1.74820143885 (suicidaility)

Pulling the inference output from the Linux VM (to a local Windows machine)

Download SCP: http://the.earth.li/~sgtatham/putty/latest/x86/pscp.exe
Run: pscp bnn@hostname:/home/bbn/inference.out c:\temp

Value of Information (VOI)

The VOI (Variables Of Interest) command extracts the variables used in the trained model, and, given an input dataset, computes their mutual information, relative to the target variable. We felt that reporting high VOI vars was useful for analysts understanding which input variables contribute the most to the model. For readability, we truncated the reported list to 10.This is accomplished with the command;

poses voivar -d va32-pairs-allowed-asimple.csv -m model-32.mod -p group -o model-32.voi |tee voi.out

Results in;

voi(WAS_FRIGHTENING=1.41475102542)
voi(UPSETTING_IN=1.41475102542)
voi(UPSETTING_THAT=1.41475102542)
voi(WAS_HORRIBLE=1.41475102542)
voi(INFORMED_COLON=1.41229756021)
voi(CONSENT_COLON=1.41229756021)
voi(TEST_ALLOWS=1.41175766695)
voi(SATURATION_ROOM=1.41175766695)
voi(NURS=1.41175766695)
voi(PREPROCEDURE_DATE=1.41175766695)
