# Supplementary material for: Predicting the Risk of Suicide by Analyzing the Text of Clinical Notes
Source: PLoS One. 2014 Jan 28;9(1):e85733. doi: 10.1371/journal.pone.0085733 (PMC3904866; doi:10.1371/journal.pone.0085733)
Supplement: Appendix S3 — Machine Learning Libraries and Methods: This section is provided to enclose the open source (Apache License) classifier used for the building of the statistical models, specifically for the purposes of study reproducibility. A detailed account of these tools is intended for another publication. (ZIP) [file pone.0085733.s003.zip › Appendix3/ML_libs/man.pdf]

POSES: User Manual (man)

Modified Feb 19, 2013

NAME

poses - poses machine learning tool

SYNOPSIS

poses -h|--help

poses moses -d data_file -m model_file -p target_variable [-e fitness_evals] [-F feature_selection_options] [-n output_levels] [-t thresholds] [-l lower_neutral] [-u upper_neutral] [-M moses_options] [-D delta] [-s max_levels] [--runs num_runs] [-V] [--mpi num_procs] [--normalize-counts]

poses inference -m model_file -p target_variable -c testcase_file [--report-levels]

poses cross-validate -d data_file -p target_variable [-e fitness_evals] [-f number_of_folds] [-g num_fold_sessions] [-F feature_selection_options] [-n output_levels] [-t thresholds] [-l lower_neutral] [-u upper_neutral] [-M moses_options] [-D delta] [-s max_levels] [--runs num_runs] [-V] [--mpi num_procs] [-r rand_seed]

poses sentiment -m model_file

poses wordle -m model_file -d data_file

poses voivar -d data_file -m model_file -p target_variable [-b] [-o output_file]

DESCRIPTION

poses is a machine-learning tool based on the MOSES program learning library. It provides, in a single interface, both the training and the inference parts of machine learning, as well as integrating feature selection and thresholding.

EXAMPLES

poses moses -d bank.dat -m bank.model -pQ3 -e50000 -F"-asimple -C30" -M"-v12 -Z1"

Train moses on the bank.dat dataset, and generate the bank.model model file as output. Use the Q3 column in the dataset as the dependent variable for regression. The -F flag specifies the options that will be passed to the feature-selection command, and the -M flag specifies the options that will be passed to the moses command.

poses inference -m bank.model -pQ3 -c bank.hcs

Perform inference on the bank.hcs file, using the previously learned bank.model file for the regression model.

OVERVIEW

poses provides a number of convenience and integration abilities on top of the MOSES machine learning system. In particular, poses integrates the feature-selection and training stages into one, as well as providing a data thresholding capability. poses also supports various post-training functions, including using the learned model to perform inference. On compute clusters equipped with MPI (OpenMPI or MPICH2), poses can distrubte processing to multiple nodes.

moses is a program-learning system: given a table of discrete or continuous input values, and a target output value, it will attempt to learn an ensemble of combo programs that accurately reproduces the output, given the inputs. Thus, after training, each resulting program (or 'model') can be used to perform inference on hitherto-unseen input values. The combo language itself is a very simple language, intended only for expressing and evaluating expressions consisting of arithmetic and logical operators. See the moses man page for details.

To improve over-all accuracy on noisy datasets, and to reduce the effects of unintended over-fitting, an ensemble of combo programs can be trained on the input data. The inferred output is then obtained by averaging over the predicted output values of each combo model.

feature-selection is a simple utility for performing feature selection on tables of input data. It supports several different mutual-information-based algorithms to discover relevant features. See the feature-selection man page for details.

poses optionally performs thresholding to turn continuous-valued datasets into boolean-valued datasets. This may be done on both input and output features, so that the datasets passed to moses are purely boolean. By default, thresholding is enabled; the thresholding of input values can be disabled by specifying the -s0 option. The default thresholding for input features is around the mean: a boolean value indicates, true of false, whether an input value is above or below its mean. Specifying the -D option will threshold input values into multiple boolean features, in multiples of the standard deviation. The -s option is used to specify how many boolean features should be created per continuous-valued input feature. The predicted (output) values are also thresholded; the -n and -t options may be used to set and control these.

OPTIONS

Options fall into two classes: those for specifying inputs and outputs, and those controlling the algorithm.

General options

-h, --help

Print option summary, and exit.

-V, --verbose

Generate moses and feature-selection log files. These will be written to the current working directory.

Input and Output Specification Options

These options control how input data is specified and interpreted, and how output is generated.

poses can read data files in one of two different formats: DSV (delimiter-separated values) or sparse-CSV. The DSV format is a superset of the commonplace comma-separated value format: the input can consist of UTF8 data, separated by commas, tabs or whitespace. The appearance of # ; or ! in the first column denotes a comment; comment lines will be ignored. The first non-comment row in the file (if any) is taken to hold column labels.

The sparse-CSV format consists of rows in the form val1, val2, val3, key1 : kval1, key2 : kval2. The initial columns are the "fixed" columns; here there are three fixed columns. These are followed by colon-separated key-value pairs. All entries must be comma-spearated; tab and whitespace-delimited files are not supported. The key-value pairs must have whitespace on both sides of the colon, else the colon will not be recognized as the key-value separator. The first non-comment line in the file is assumed to be column labels for the fixed columns.

The target column is specified with the -p option with a column name.

-d filename, --data-file=filename

The filename specifies the input data file. The input table must be in DSV or sparse-CSV format, as described above. This option may be used multiple times, to specify multiple files. These files will be concatenated to form the overall input dataset. Sparse and non-sparse files can be mixed together. All files must contain one column that is the target variable.

-m filename, --model-file=filename

The filename specifies the model file. For the moses mode, the model file will be written when training is completed. For the other modes, the model file is used as input, and is assumed to have been previously created by poses in the training mode.

-p varname, --target-variable=varname

Specify the name of the variable to predict; this should be one of the column labels in the input dataset. That is, the first non-comment row of the input dataset should consist of column labels. One of the columns is presumed to contain the "dependent variable" or the "target variable" for regression. This flag is used to name that variable.

-c testfile, --test-case-file=filename

The test file specifies the test-case file. This file contains the data on which the inference command will perform inference; it is a required option for this command. The test-case file must consist of colon-separated key : value pairs, one per line. Lines beginning with # ; or ! will be treated as comments and will be ignored. This flag can be used multiple times to specify multiple files; a distinct inference will be made on each input testcase file.

Training Algorithm Control Options

These options provide overall control over the training algorithm execution.

-F opts, --feature-selection-opt=opts

Specify options to be passed to the feature-selection pre-processor. If no options are specified, then feature selection will not be performed. The full set of feature-selection options are documented in the feature-selection man page. Feature selection is used to winnow down the total number of features, by discarding those that seem to be uncorrelated with the target variable. Performing this filtering can greatly reduce the learning time, at the risk of possibly discarding some questionably relevant data. To pass multiple feature-selection options, they must be surrounded by double quotes. So, for example, -F "-ammi -C15".

-M opts, --moses-opt=opts

Specify options to be passed to the moses program learner. MOSES has a large number of options that control its behavior and performance. See the moses man page for details. To pass multiple moses options, they must be surrounded by double quotes. So, for example, -M "-v12 -Z1".

-e fitness_evals, --fitness-evaluations=N

Specify the number of fitness evaluations that MOSES will perform while searching for a model of the input dataset. In general, the greater the number of fitness evaluations, the better the model; see however, comments about over-fitting in the accuracy section, below. The default value is 1000; however, recommended values are in the range of ten thousand to many millions.

--runs=number_of_runs

Specify the number or MOSES runs to perform when building an ensemble of models with the training or cross-validation commands. Each run will result in a handful of combo programs being created that model the data. By specifying a reasonably large number of runs (ten to one-hundred), the resulting ensemble of models will smooth out or average over the effects of over-fitting to a noisy dataset. During the inference stage, the average of all the models will be reported as the predicted output. The confidence of a prediction is then reported as the fraction of all models that agreed on the majority-vote answer.

-r rand_seed, --rand-seed=N

Specify the initial random number seed for the pseudo-random number generator. The random number generator is used only when performing random selection for the cross-validate command.

Bag of Word Phrases Support

poses has several options that can be used when the input dataset is a bag of words or a bag of word-phrases. In this case, the dataset is assumed to consist of words (or phrases) and a count; that is, columns correspond to distinct words, and column entries are the word-count for that record.

--normalize-counts

Normalize the input dataset to make it independent of the record size. This is done by dividing all counts by a fixed constant, so that the most frequent word (in the entire dataset) occurs an average of just once per record. The goal of normalization is to overcome differences of size between the training records, used to create the model, and the inference set size. Without normalization, the size of the inference set (the number of words in the inference set) must be more or less the same as that of each of the original training records. With normalization, inference can be performed on test records whose overall size differ significantly from those used in the training set; in this case, the size of the test set is also normalized to the largest count appearing in the test set, before the inference is made.

Target Variable Thresholding

poses has several different modes of training, depending on the type of the target variable, and whether binary classification (thresholding) is requrested.

By default, floating-point target variables are thresholded into three ranges: low, medium and high, centered upon the median value of the target variable, with the low and high thresholds located at 0.8 and 1.2 times the median value. The target variable is "booleanized" at each threshold: that is, training will create a binary classifier, discerning whether the target is above or below the given threshold. For N thresholds, this requires N training sessions; and although poses will manage these automatically, the overall training time will increase in proportion to the number of thresholds. The number and location of thresholds can be specified with the -n, -l, -u and -t options, described below.

Thresholding on floating-point target variables can be disabled by specifying the -n1 option, to indicate that only one level (range) should be used. In this case, only one moses training session will be performed; note, however, that training moses on floating-point variables can run slower than training on boolean values.

Enumerated target variables can similarly be handled "natively" by moses, or by converting them into a set of boolean classification problems. Enumerated variables are those that take on one of a discrete set of string values (such as "red", "yellow", "green"). If the -n1 option is specified, then only one moses training session is performed, with moses attempting to find a classifier that is accurate for each of the target values. Alternately, by using the -n and -t options, poses will split up the problem into multiple binary classification tasks, with one classification task for each enum value. If -n is used to specify fewer "levels" than there are enum values, then only the most populous enums will be classified (that is, binary classifiers will be created only for those enums that appear the most frequently in the dataset). Alternately, the -t option can be used to explicitly name the target values to be used during binary classification.

-n num_levels, --number-of-levels=num_levels

Specify the number of levels for the target variable. Specifying num_levels to be 1 disables thresholding; the default value is 3. For floating-point-valued targets, if the locations of the thresholds are not explicitly specified (with the -t, -l or -u options), then they are computed automatically. For -n2, the single threshold is placed at the median value of the target variable; or, if the -t option was used, the threshold is placed there. For -n3, the two thresholds are placed at 0.8 and 1.2

times the median value; or, if a single -t option was used, the two thresholds are located at 0.8 and 1.2 of that value. For larger n's, thresholds are created at evenly-spaced values between the smallest and the largest observed values of the target. For enum-valued targets, and num_levels greater than one, then binary classifiers will be created for the num_levels most populated enums in the dataset.

-l low, --lower-neutral=low

For floating-point targets, specify the location of the lower threshold. This will be located at 't_md * (1 - low)'. Here, 't_md' is either the median value of the target, or, if the -t option was used, it is the value specified by -t. The default value for low is 0.2. It is ignored if the number of output levels is not 3, or the target is not floating point.

-u hi, --upper-neutral=hi

For floating-point targets, specify the location of the upper threshold. It will be located at 't_md * (1 + hi)'. Here, 't_md' is either the median value of the target, or, if the -t option was used, it is the value specified by -t. The default value for hi is 0.2. It is ignored if the number of output levels is not 3, or the target is not floating point.

-t threshold, --threshold=threshold

For floating-point targets, this defines an output threshold for a binary classifier. That is, a classifier will be created that returns true whenever the targetVar >= threshold. If n levels were specified with the -n option, and n is greater than 3, then the -t option must be used n-1 times. For enum-valued targets, this option can be used to explicitly name which target values should get a binary classifier. Use multiple instances of the -t option, one for each value.

--report-levels

If specified, then the level number, instead of the predicted value will be reported by the inference command. Level numbers range from 0 to N-1 where N is the number of output levels (specified by the -n option).

Input Variable Thresholding

poses optionally applies thresholding to each continuous-value input feature 'f', to convert it into a single boolean-valued feature 'f_0'. This binary feature takes on values of T or F, indicating whether or not 'f' is above or below it's mean value 'm_0'. That is, the binary feature 'f_0' indicates whether 'f' lies in the interval (mean(f), +inf) or not.

Thresholding is performed by default, with exactly one binary feature created for each input feature. The thresholding of input values can be disabled by specifying the -s0 option on the command line. The speed of learning and the accuracy of the results can sometimes be dramatically affected by whether or not thresholding is enabled, and the number of thresholds used. Classification will typically be more accurate, if additional thresholding is done. The number of thresholds to use may be specified with the -s option, and their locations with the -D flag.

Each new boolean feature 'f_n' will be a binary indicator of whether 'f' lies above or below (to the right or left) of the threshold t_n = mean(f) + delta * n * stddev(f). Here, 'stddev' is the standard deviation of 'f', while 'mean' is the mean value.

-D delta, --delta=delta
: Specify a value delta > 0 indicating the distance between thresholds. The value is interpreted as a fraction of the standard deviation of the input variable. If not specified, the delta value is assumed to be 1.0.

-s num, --number-of-thresholds=num
: Specify the number of thresholds to create. If num is zero, thresholding of input values is disabled. If not specified, the default is to create one threshold, at the mean value of the input feature. If num is even, then the threshold 't_0' is not created (so, for example, for -s 2, only 't_1' and 't_{-1}' are created). If -s is not specified, but the -D option gives a positive value, then enough thresholds are created to cover the entire range of observed values for the particular input feature. If neither the -s or the -D options are given, then only a single threshold 't_0' is created at the mean value of 'f'.

: If the values of an input feature are strangely distributed, and num is large, it can happen that fewer than num thresholds will be created; this will occur when there are no values beyond the threshold.

Cross-validation

The cross-validate command may be used to perform cross-validation of the of the learned models against hold-outs from the dataset. This is useful for evaluating the expected accuracy of the learning process, and to test for signs of over-fitting.

Cross-validation is performed by splitting the input data-set into two parts: the train subset, and the hold-out or test subset. By default, these are 4/5'ths and 1/5'th of the dataset size. A model is then trained on the train subset. The resulting model is evaluated for accuracy (and precision, recall, when appropriate) against both subsets (separately). The accuracy of the model on the train subset simply indicates how well the model was able to fit the training data. The accuracy of the model on the test subset is more interesting: it indicates how well the model is able to predict and classify previously-unseen data.

If the data is relatively noise-free, and the length of training was sufficiently long, the accuracy on the train subset should be very high. A model with good predictive ability will score well on the test subset, and ideally, close to that of the train subset. A sharply worse accuracy indicates over-fitting: the model is encoding some pattern in the train subset that simply does not exist in the test subset.

The -f num_folds option is used to specify the number of folds to be performed during cross-validation. By default,
num_folds=5. If N=num_folds, the validation will be performed N times, with each evaluation done on a train set of size (N-1)/N and test set the size of 1/N, rotating over each of the N equal-sized test sets. That is, the input dataset is split into N pieces. For each fold, N-1 of these are grouped into the train set, with the last used for the test set. For the next fold, the pieces are regrouped in cyclic, round-robin fashion, for a total of N folds and N validations. Note that, as a result, the cross-validate command will typically run about N times longer than the moses command. The final printed accuracy results will be for the sum (average) of the folds.

Run-time can be shortened by explicitly specifying fewer validation sessions, using the -g num_sessions option. By default, the number of validation sessions is equal to the number of folds, as explained above. Specifying a smaller number cuts short the round-robin validation tournament.

A number of sessions that is larger than the number of folds can be specified. In this case, random selection is performed instead of round-robin selection. The fractional sizes of the train and test sets are still (N-1)/N and 1/N as before; however, different records are allocated to the one or other based on a (pseudo-) random number draw. The pseudo-random number seed can be varied with the -r option.

The printed accuracy matrix presents a bin-count of expected answers, in rows, versus the results obtained by the classifier, in columns. A perfectly accurate classifier would have entries only along the diagonal. That is, a perfect classifier would always produce the expected

result. Off-diagonal entries are those where the classifier produced one result, but the correct answer was otherwise. The printed matrix is followed by a single floating-point number summarizing the fraction of the entries on the diagonal (the sum of diagonal entries divided by the sum of all entries).

When the classifier is trained on only two output values, then additional information is printed: the recall of the model, and the precision. Recall is defined as the fraction of positive results that were correctly identified as being positive. Precision is defined as the fraction of positive results, excluding incorrectly identified negative results. The definitions used here are the standard text-book definitions for these two quantities.

Inference

The central idea of training a model on a dataset is to be able to later use that model to perform inference on new, previously unseen, test data. The inference command provides this ability. In order to do inference, both a model and a test-case file must be specified. The inference command applies the model to the test case, and infers the most likely classification for the case. This is printed on stdout, followed by a confidence ranking in this result.

Inference is actually performed on an ensemble of models, and the reported confidence value is the fraction of the ensemble that agrees on the output. During training, several or even many models of the training data are built, depending on the setting of the --runs option. Each of these models can be used to make an inference on the test case. The models usually, but not always, agree. The reported result is determined by voting: it is the most popular one of all the different results. The confidence is then the fraction of the vote that went to this result. For binary classifiers, the confidence is never less than 1/2; for multi-valued classifiers, the confidence is greater than 1/N of the number N of different possible outputs.

Sentiment and Wordles

poses supports two different commands that help provide some insight into the content of a model. The sentiment command prints a ranked list of positive and negative sentiment keywords appearing in a model. These are the words that appear in one or more of the ensemble models, ranked by how often they appear in the ensemble. A keyword is considered to be positive if it appears as a greater-than threshold, and negative if it appears as a less-than threshold. Thus, if a model requires that the word "happy" appear more than three times, then it is considered to be a postive keyword. Any given word might appear as both a positive or a negative keyword: some clause may require it to appear more than a certain number of times, while a different clause might require it to appear less than that. If the input values are not

thresholded (the -s option specified zero) then there is no distinction between positive and negative, and only one list of keywords is printed, ranked by how often they appear in the ensemble models.

The wordle command is similar, but it shows wordcounts obtained from a dataset. It extracts a list of positive keywords from the model, and the corresponding word counts from the dataset. These keywords and counts are printed in ranked order, for each cohort. Here, a cohort is defined as that subset of the dataset that shares a common target (output) value; thus, the word counts are shown, subtotalled for each cohort.

MPI Support

poses is able to make use of MPI interfaces (OpenMPI or MPICH2) to improve processing time. MPI, an abbreviation for the "Message Passing Interface" standard, is used to distribute processing across multiple CPU nodes in a compute cluster. It is commonly used in scientific computing to distribute algorithms on supercomputers. Because the primary algorithm used in poses belongs to the class of "embarassingly parallel" algorithms, the operation of poses is easily speeded on compute clusters.

In order to make use of this, the standard MPI system management tools must be installed on the target hardware. In addition, a version of the moses binary, compiled with MPI support enabled, must be installed. The rest of this section assumes familiarity with basic MPI job submission.

To enable MPI processing, simply include the --mpi=num_procs option for the moses or accuracy commands.for the moses or cross-validate commands. CPU usage is maximized by setting the value of num_procs to 2N+1, where N is the number of physical nodes in the cluster. The double-counting helps make sure that all available CPU processing power will be used; a single instance, although multi-threaded, may not always be able to make full use of all cores on a single node. Processing is structured so that there is one master control or 'root' process, which does very little work other than to coordinate the other 'worker' processes, and to collect and collate results. Thus, the setting --mpi=2 is the minimum setting that makes sense: one process will be mostly idle, acting as a control, and one process will perform all of the work.

Processes are started by issuing the mpirun command. This may be over-ridden by setting the MPIRUN environment variable. The hosts file is assumed to be located at ~/mpd.hosts. The location of the hosts file may be over-ridden by setting the MPI_HOSTS environment variable.
