# Supplementary material for: Predicting the Risk of Suicide by Analyzing the Text of Clinical Notes
Source: PLoS One. 2014 Jan 28;9(1):e85733. doi: 10.1371/journal.pone.0085733 (PMC3904866; doi:10.1371/journal.pone.0085733)
Supplement: Appendix S3 — Machine Learning Libraries and Methods: This section is provided to enclose the open source (Apache License) classifier used for the building of the statistical models, specifically for the purposes of study reproducibility. A detailed account of these tools is intended for another publication. (ZIP) [file pone.0085733.s003.zip › Appendix3/ML_libs/moses-3.4.0.tar.bz2!/moses-3.4.0/moses3/learning/moses/documentation/Select_Exemplar.html]

Tuning MOSES | OpenCog Brainwave


# Tuning MOSES

Posted on February 7, 2012 by Linas Vepstas

The following is a verbatim copy of a blog entry about MOSES. In fact, it is mostly about
tuning the operation of the select\_exemplar() code so that MOSES finds solutions
more quickly.

I’ve been studying MOSES recently, with an eye towards performance tuning it. Turns out optimization algorithms don’t always behave the way you think they do, and certainly not the way you want them to.

Given a table of values, MOSES will automatically learn a program that reproduces those values. That is, MOSES performs table regression: given N columns of “input” values, and one column of “output”, MOSES will create a program that outputs the output, given the inputs.  MOSES can deal with both floating point and boolean inputs, and thus can learn, for example, expressions such as ((x<2) AND b) OR (x\*(y+1) >3).  MOSES programs are real “programs”: it can even learn branches and loops, although I won’t explore that here. For performance tuning, I studied the 4-parity problem: given 4 input bits, compute the parity bit.  Written out in terms of just AND, OR and NOT, this is a fairly complex expression, and is rather non-trivial to learn.

MOSES performs learning by keeping a “metapopulation” of example programs, or “exemplars”.  These are graded on how well the match the output, given the inputs. For the 4-parity problem, there are 2^4^=16 different possible inputs; a given program may get any number of these correct.  For example, there are 16 ways to get one answer wrong; 16×15 ways to get two wrong, 16×15×14 ways to get three wrong, *etc.* This is the binomial distribution: (16 choose *k*) ways to get *k* answers wrong, in general. But this doesn’t mean that there are only 16 different programs that get one answer wrong: there are zillions: some simple, some very very complex.

As MOSES iterates, it accumulates a metapopulation of programs that best fit the data. As soon as it finds a program that gets more correct answers than the others, the old metapopulation is wiped out; but then, it starts growing again, as new programs with equal score are found.  This is shown in the following graph:

Metapopulation size as function of generation number.

The red line shows the metapopulation size (divided by 50), as a function of the generation number (that is, the iteration count).  It can be seen to collapse every time the score improves; here, the “minus score”, in green is the number of wrong answers: a perfect score has zero wrong answers; the program stops when a perfect score is reached.

In blue, the complexity of the program — actually, the complexity of the least complex program that produces the given score. Computing the parity requires a fairly complex combination of AND’s OR’s and NOT’s; there is a minimum amount of complexity such a program can have.  Here, for example, are two different programs that compute the parity perfectly, a short one:

```
﻿﻿and(or(and(or(and(!$1 !$2) and(!$3 $4)) or(!$2 !$3))
   and($1 $2) and($3 !$4))
   or(and(!$1 $2) and($1 !$2) and(!$3 !$4) and($3 $4)))
```

and a longer one:

```
or(and(or(and(or(!$1 !$3) $4) and(or($1 !$2) !$3 !$4)
   and(or($3 $4) $2)) or(and(or(!$1 !$4) $2 !$3)
   and(or(!$2 $3) $1 $4) and(!$1 !$4) and(!$2 $3)))
   and($1 !$2 $3 !$4))
```

More on complexity later.

But first: how long does it take for MOSES to find a solution to 4-parity? It turns out that this depends strongly on the random-number sequence.  MOSES makes heavy use of a random number generator to explore the problem space.  Each run can be started with a different seed value, to seed the random number generator.  Some runs find the correct solution, some take a surprisingly long amount of time. Amazingly so: the distribution appears to follow a logarithmic distribution, as in the following graph:

Runtime, showing temperature dependence

One the vertical axis, the amount of time, in seconds, to find a solution. One the horizontal axis, the order in which a solution was found, out of 20 random attempts. The way to read this graph is as follows: there is a probability Pr=1/20 chance of finding a solution in about 10 seconds. There is a Pr=2/20 chance of finding a solution in about 20 seconds, *etc.* Continuing: about a Pr=6/20 chance of finding a solution in less than about 100 seconds, and a Pr=17/20 chance of finding a solution in less than about 1000 seconds.

The shape of this graph indicates that there is a serious problem with the current algorithm. To see this, consider running two instances of the  algorithm for 300 seconds each. Per the above graph, there is a 50-50 chance that each one will finish, or a 75% chance that at least one of them will finish.  That is, we have a 75% chance of having an answer after 600 CPU-seconds.  This is better than running a single instance, which requires about 900 seconds before it has a 75% chance of finding an answer!  This is bad.  It appears that, in many cases, the algorithm is getting stuck in a region far away from the best solution.

Can we do better? Yes. Write *p* = Pr(*t<T*) for the probability that a single instance will find a solution in less time than T.  Then, from the complexity point of view, it would be nice if we had an algorithm if two instances did NOT run faster than a single instance taking twice as long; that is, if

Pr(*t<2T*) ≤ *p^2^+2p(1-p)*

The first term, *p^2^*, is the probability that both instances finished.  The second term is the probability that one instance finished, and the other one did not (times two, as there are two ways this could happen).   More generally, for *n* instances,  we sum the probability that all *n* finished, with the probability that *n-1* finished, and one did not (*n* different ways), *etc.*:

Pr(*t<nT*) ≤ *p^n^ + np^n-1^(1-p) + n(n-1)p^n-2^(1-p)^2^ + … + np(1-p)^n-1^*

This inequality, this desired bound on performance, has a simple solution, given by the exponential decay of probability:

Pr(*t<T*) = 1-exp(*-T/m*)

As before,  Pr(*t<T*) is the probability of finding a solution in less than time *T*, and *m* is the mean time to finding a solution (the expectation value). To better compare the measured performance to this desired bound, we need to graph the data differently:

This graph shows the same data as before, but graphed differently: the probability of not yet having found a solution is shown on the horizontal axis. Note that this axis is logarithmic, so that the exponential decay bound becomes a straight line.  Here, the straight purple line shows the bound for a 500 second decay constant; ideally, we’d like an algorithm that generates points below this line.

Before continuing, a short aside on the label “*temp*“, which we haven’t explained yet. During the search, MOSES typically picks one of the simplest possible programs out of the current metapopulation, and explores variations of it, it explores its local neighborhood.  If it cannot find a better program, it picks another, simple, exemplar out of the metapopulation, and tries with that, and so on.   It occurred to me that perhaps MOSES was being too conservative in always picking from among the least complex exemplars.  Perhaps it should be more adventurous, and occasionally pick a complex exemplar, and explore variations on that.   The results are shown in the green and blue lines in the graph above.  The select\_exemplar() function uses a Boltzmann distribution to pick the next exemplar to explore. That is, the probability of picking an exemplar of complexity *C* as a starting point is

exp(-*C/temp*)

where *temp* is the “temperature” of the distribution. The original MOSES algorithm used *temp*=1, which appears to be a bit too cold; a temperature of 2 seems about right.  With luck, this new, improved code will be checked into BZR by the time you read this.

There is another issue: the unbounded size of the metapopulation. When MOSES stalls, grinding away and having trouble finding a better solution, the size of the metapopulation tends to grow without bounds, linearly over time. It can get truly huge: sometimes up to a million, after a few thousand generations.  Maintaining such a large metapopulation is costly: it takes up storage, and eats up CPU time to keep it sorted in order of complexity.  Realistically, with a metapopulation that large, there is only a tiny chance (exponentially small!) that one of the high-complexity programs will be selected for the next round. The obvious fix is to clamp down on the population size, getting rid of the unlikely, high-complexity members.   I like the results so far:

Runtime, using a limited population size.

Clamping the population size clearly improves performance — by a factor of two or more, as compared to before.  However, the troublesome behavior, with some solutions being hard to discover, remains.

Now, to attack the main issue: Lets hypothesize what might be happening, that causes the exceptionally long runtimes.  Perhaps the algorithm is getting stuck at a local maximum?  Due to the knob-insertion/tweaking nature of the algorithm, there are no “true” local maxima, but some may just have very narrow exits.  The standard solution is to apply a simulated-annealing-type trick, to bounce the solver out of the local maximum.  But we are already using a Boltzmann factor, as described above, so what’s wrong?

The answer seems to be that the algorithm was discarding the “dominated”  exemplars, and was keeping only those with the best score, and varying levels of complexity. It only applied the Boltzmann factor to the complexity.  What if, instead, we applied the Boltzmann factor to mixture of score and complexity?  Specifically, lets try this:

exp(-(*C – S W) / temp*)

Here, *C* is the complexity, as before, while *S* is the score, and *W* a weight.  That is, some of the time, the algorithm will select exemplars with a poor score, thus bouncing out of the local maximum.  Setting *W* to zero regains the old behavior, where only the highest-scoring exemplars are explored.  So .. does this work? Yes! Bingo! Look at this graph:

Score-weighted Annealing

Two sets of data points, those for *W*=1/4 and 1/3, look very good.  Its somewhat strange and confusing that other *W* values do so poorly.   I’m somewhat worried that the *W*=1/4 value is “magical”: take a look again at the very first graph in this post.  Notice that every time a better solution is found, the complexity jumps by about 4.  Is this ﻿the *W*=1/4 value special to the 4-parity problem? Will other problems behave similarly, or not?

I’m continuing to experiment. Collecting data takes a long time. More later… The above was obtained with the code in bzr revision 6573, with constant values for “temp” and “weight” hand-edited as per graphs. Later revisions have refinements that fundamentally alter some loops, including that in select\_exemplar(), thus altering the range of reasonable values, and the meaning/effect of some of these parameters. Sorry

I do hope that this post does offer some insight into how MOSES actually works.  A general overview of MOSES can be found on the MOSES wiki, as well as a detailed description of the MOSES algorithm. But even so, the actual behavior, above, wasn’t obvious, at least to me, until I did the experiments.

## Appendix: Parallelizability

A short footnote about the generic and fundamental nature of the exponential decay of time-to-solution in search problems. Earlier in this post, there is a derivation of exponential decay as the result of running *N* instances in parallel.   How should this be understood, intuitively?

Search algorithms are, by nature, highly parallelizable: there are many paths (*aka exemplars*) to explore; some lead to a solution, some do not.  (An exemplar is like a point on a path: from it, there are many other paths leading away).  A serial search algorithm must implement a chooser: which exemplar to explore next? If this chooser  is unlucky/unwise, it will waste effort exploring exemplars that don’t lead to a solution, before it finally gets around to the ones that do.  By contrast, if one runs *N* instances in parallel (*N* large), then the chooser doesn’t matter, as the *N-1* ‘bad’ exemplars don’t matter: the one good one that leads to a solution will end the show.

Thus, we conclude: if a serial search algorithm follows the exponential decay curve, then it has an optimal chooser for the next exemplar to explore.  If it is “worse” than exponential, then the chooser is poorly designed or incapable.  If it is “better” than exponential, then that means that there is a fixed startup cost associated with each parallel instance: cycles that each instance must  pay, to solve the problem, but do not directly advance towards a solution.  Ideal algorithms avoid/minimize such startup costs.  Thus, the perfect, optimal algorithm, when run in serial mode, will exhibit exponential solution-time decay.

The current MOSES algorithm very nearly achieves this for 4-parity, as shown in this last figure, which compares the original chooser to the current one (bzr revno 6579)
