# Supplementary material for: Predicting the Risk of Suicide by Analyzing the Text of Clinical Notes
Source: PLoS One. 2014 Jan 28;9(1):e85733. doi: 10.1371/journal.pone.0085733 (PMC3904866; doi:10.1371/journal.pone.0085733)
Supplement: Appendix S3 — Machine Learning Libraries and Methods: This section is provided to enclose the open source (Apache License) classifier used for the building of the statistical models, specifically for the purposes of study reproducibility. A detailed account of these tools is intended for another publication. (ZIP) [file pone.0085733.s003.zip › Appendix3/ML_libs/moses-3.4.0.tar.bz2!/moses-3.4.0/moses3/learning/moses/documentation/Genetic_Crossover.html]

Genetic Crossover in MOSES | OpenCog Brainwave


# Genetic Crossover in MOSES

Posted on March 20, 2012 by Linas Vepstas

MOSES is a system for learning programs from input data.  Given a table of input values, and a column of outputs, MOSES tries to learn a program, the simplest program that can reproduce the output given the input values. The programs that it learns are in the form of a “program tree” —  a nested concatenation of operators, such as addition or multiplication, boolean AND’s or OR’s, if-statements, and the like, taking the inputs as arguments.  To learn a program, it starts by guessing a new random program.  More precisely, it generates a new, random program tree, with as-yet unspecified operators at the nodes of the tree. So, for example, an arithmetic node maybe be addition, or subtraction, or multiplication, division, or it may be entirely absent.  It hasn’t yet been decided which.   In MOSES, each such undecided node is termed a “knob”, and program learning is done by “turning the knobs” until a reasonable program is found.  But things don’t stop there: once a “reasonable” program is found, a new, random program tree is created by decorating this “most reasonable” program with a new set of knobs.  The process then repeats: knobs are turned until an even better program is found.

Thus, MOSES is a “metalearning” system: it consists of an outer loop, that creates trees and knobs, and an inner loop, that finds optimal knob settings.  Both loops “learn” or “optimize”; it is the nesting of these that garners the name “metalearning”. Each loop can use completely different optimization algorithms in its search for optimal results.

The rest of this post concerns this inner loop, and making sure that it finds optimal knob settings as quickly and efficiently as possible. The space of all possible knob settings is large: if, for example, each knob has 5 possible settings, and there are 100 knobs, then there is a total of 5^100^ possible different settings: a combinatorial explosion. Such spaces are hard to search. There are a variety of different algorithms for exploring such a space. One very simple, very traditional algorithm is “hillclimbing”. This algo starts somewhere in this space, at a single point, say, the one with all the knobs set to zero. It then searches the entire local neighborhood of this point: each knob is varied, one at a time, and a score is computed. Of these scores, one will be best. The corresponding knob setting is then picked a the new center, and the process then repeats; it repeats until there is no improvement: until one can’t “climb up this hill” any further. At this point, the inner loop is done; the “best possible” program has been found, and control is returned to the outer loop.

Hill-climbing is a rather stupid algorithm: most knob settings will result in terrible scores, and are pointless to explore, but the hill-climber does so anyway, as it has no clue as to where the “good knobs” lie. It does an exhaustive search of the local neighborhood of single-knob twists.  One can do much better by using estimation-of-distribution algorithms, such as the Bayesian Optimization Algorithm.  The basic premise is that knob settings are correlated: good settings are near other good settings.  By collecting statistics and computing probabilities, one can make informed, competent guesses at which knob settings might actually be good.  The downside to such algorithms is that they are complex:  the code is hard to write, hard to debug, and slow to run: there is a performance penalty for computing those “educated guesses”.

This post explores a middle ground: a genetic cross-over algorithm that improves on simple hill-climbing simply by blindly assuming that good knob settings really are “near each other”, without bothering to compute any probabilities to support this rash assumption.  The algorithm works; headway can be made by exploring only the small set of knob settings that correlate with previous good knob settings.

To explain this, it is time to take a look at some typical “real-life” data. In what follows, a dataset was collected from a customer-satisfaction survey; the goal is to predict satisfaction from a set of customer responses.  The dataset is a table; the outer loop has generated a program decorated with a set of knobs.  Starting with some initial knob setting, we vary each knob in turn, and compute the score. The first graph below shows what a  typical “nearest neighborhood” looks like.  The term “nearest neighborhood” simply means that, starting with the initial knob setting, the nearest neighbors are those that differ from it by exactly one knob setting, and no more.  There is also a *distance*=2 neighborhood: those instances that differ by exactly two knob settings from the “center” instance.  Likewise, there is a *distance*=3 neighborhood, differing by 3 knob settings, *etc.*  The size of each neighborhood gets combinatorially larger.  So, if there are 100 knobs, and each knob has five settings, then there are 5 × 100=500 nearest neighbors. There are 500 × 499 / 2 = 125K next-nearest neighbors, and 500 × 499 × 498 / (2 × 3) = 21M instances at *distance*=3. In general, this is the binomial coefficient: (500 choose *k*) for distance *k*. Different knobs, however, may have more or fewer than just 5 settings, so the above is just a rough example.

Nearest Neighbor Scores

The above graph shows the distribution of nearest neighbor scores, for a “typical” neighborhood. The score of the center instance (the center of the neighborhood) is indicated by the solid green line running across the graph, labelled “previous high score”.  All of the other instances differ by exactly one knob setting from this center.  They’ve been scored and ranked, so that the highest-scoring neighbors are to the left.  As can be seen, there are maybe 15 instances with higher scores than the center, another 5 that seem to tie.  A slow decline is followed by a precipitous drop; there are another 80 instances with scores so bad that they are not shown in this figure.  The hill-climbing algo merely picks the highest scorer, declares it to be the new center, and repeats the process.

All of the other neighborhoods look substantially similar. The graph below shows an average over many generations (here, each iteration of the inner loop is one generation). The jaggedness above is smoothed out by averaging.

Nearest Neighbor Score Change

Rather than searching the entire neighborhood, one would like to test only those knob settings likely to yield good scores. But which might these be?  For nearest neighbors, there is no way to tell, without going through the bother of collecting statistics, and running them through some or another Bayesian estimation algorithm.

However, for more distant neighbors, there is a way of guessing and getting lucky: perform genetic cross-overs.  That is, take the highest and next-highest scoring instances, and create a new instance that differs from the center by two knob-settings, the two knobs associated with the two high scorers.  In fact, this new instance will very often be quite good, beating both of its parents.   The graph below shows what happens when we cross the highest scorer with each one of the next 70 highest. The label “1-simplex” simply reminds us that these instances differ by exactly two knob settings from the center.  More on simplexes later.  The green zero line is located at the highest-scoring single-knob change.  The graph shows that by starting here, and twiddling the next-most-promising knob, can often be a win. Not always: in the graph below, only 4 different knobs showed improvement. However, we explored relatively few instances to find these four; for this dataset, most exemplars have thousands of knobs.

Average Score Change, 1-simplex

The take-away lesson here is that we can avoid exhaustive searches by simply crossing the 10 or 20 or 30 best instances, and hoping for the best. In fact, we get lucky with these guesses quite often. What happens if, instead of just crossing two, we cross three of the top scorers?  This is the “2-simplex”, below:

Average Score Change, 2-simplex

Notice that there are now even more excellent candidates!  How far can we go?  The 3-simplex graph below shows the average score change from crossing over four high-scoring instances:

Average Score Change. 3-simplex

The term “crossover” suggests some sort of “sexual genetic reproduction”. While this is correct, it is somewhat misleading.   The starting population is genetically very uniform, with little “genetic variation”.  The algorithm starts with one single “grandparent”, and produces a population of “parents”, each of which differ from the grandparent by exactly one knob setting. In the “nearest neighborhood” terminology, the “grandparent” is the “center”, and each “parent” is exactly one step away from this center. Any two “parents”, arbitrarily chosen, will always differ from one-another by exactly two knob settings. Thus, crossing over two parents will produce a child that differs by exactly one knob setting from each parent, and by two from the grandparent. In the “neighborhood” model, this child is a distance=2 from the grandparent.   For the case of  three parents, the child is at distance=3 from the grandparent, and so on: four parents produce a child that is distance=4 from the grandparent.  Thus, while “sexual reproduction” is a sexy term, it looses its punch with the rather stark uniformity of the parent population; thinking in terms of “neighbors” and “distance” provides a more accurate mental model of what is happening here.

The term “simplex” used above refers to the shape of the iteration over the ranked instances: a 1-simplex is a straight line segment, a 2-simplex is a right triangle, a 3-simplex is a right tetrahedron. The iteration is performed with 1, 2 or 3 nested loops that cross over 1, 2 or 3 instances against the highest. It is important to notice that the loops do not run over the entire range of nearest neighbors, but only over the top scoring ones. So, for example, crossing over the 7 highest-scoring instances for the 3-simplex generates 6!/(6-3)! = 6 × 5 × 4 = 120 candidates. Scoring a mere 120 candidates can be very quick, as compared to an exhaustive search of many thousands of nearest neighbors. Add to this the fact that most of the 120 are likely to score quite well, whereas only a tiny handful of the thousands of nearest neighbors will show any improvement, and the advantage of this guessing game is quite clear.

So what is it like, after we put it all together? The graph below shows the score as a function of runtime.

Score as function of time

In the above graph, each tick mark represents one generation. The long horizontal stretches between tick marks shows the time taken to perform an exhaustive nearest-neighborhood search. For the first 100 seconds or so, the exemplar has very few knobs in it (a few hundred), and so an exhaustive search is quick and easy. After this point, the exemplars get dramatically more complex, and consist of thousands of knobs. At this point, an exhaustive neighborhood search becomes expensive: about 100 seconds or so, judging from the graph. While the exhaustive search is always finding an improvement for this dataset, it is clear that performing some optimistic guessing can improve the score a good bit faster. As can be seen from this graph, the algorithm falls back to an exhaustive search when the optimistic simplex-based guessing fails to show improvement; it then resumes with guessing.

To conclude: for many kinds of datasets, a very simple genetic-crossover algorithm combined with hillclimbing can prove a simple but effective search algorithm.

*Note Bene*: the above only works for some problem types; thus it is not (currently) enabled by default. To turn it on, specify the -Z1 flag when invoking moses.

## Appendix

Just to keep things honest, and to show some of the difficulty of algorithm tuning, below is a graph of some intermediate results taken during the work.  I won’t explain what they all are, but do note one curious feature:  the algos which advance the fastest initially seem to have trouble advancing later on.  This suggests a somewhat “deceptive” scoring landscape: the strong early advancers get trapped in local maxima that they can’t escape.   The weak early advancers somehow avoid these traps.  Note also that results have some fair dependence on the random number generator seed; different algos effectively work with different random sequences, and so confuse direct comparison by some fair bit.

Many Different Algorithms
