# Supplementary material for: Predicting the Risk of Suicide by Analyzing the Text of Clinical Notes
Source: PLoS One. 2014 Jan 28;9(1):e85733. doi: 10.1371/journal.pone.0085733 (PMC3904866; doi:10.1371/journal.pone.0085733)
Supplement: Appendix S3 — Machine Learning Libraries and Methods: This section is provided to enclose the open source (Apache License) classifier used for the building of the statistical models, specifically for the purposes of study reproducibility. A detailed account of these tools is intended for another publication. (ZIP) [file pone.0085733.s003.zip › Appendix3/ML_libs/moses-3.4.0.tar.bz2!/moses-3.4.0/doc/moses/QuickGuide.pdf]

MOSES - Quick Start

Predrag Janicic, Revised by Linas Vepstas

3 November 2008, revised 30 January 2012

Abstract

Meta-optimizing semantic evolutionary search (MOSES) is a new approach to program evolution, based on representation-building and probabilistic modeling. MOSES has been successfully applied to solve hard problems in domains such as computational biology, sentiment evaluation, and agent control. Results tend to be more accurate, and require fewer objective function evaluations, than other program evolution systems, such as genetic programming or evolutionary programming. Best of all, the result of running MOSES is not a large nested structure or numerical vector, but a compact and comprehensible program written in a simple Lisp-like mini-language.

Contents

1 Introduction 3

2 Copyright Notice 3

3 Overview 3

4 Terminology 4

5 MOSES Algorithm 7

6 Installation 9

7 Source Files and Folders 9

8 Types, Structures, and Classes 10

8.1 Structured expression trees 10
8.2 Representation building 10
8.3 Knobs 11
8.4 Packed Knobs, Instances 12
8.5 Representation, revisited 13
8.6 Scoring 13
8.7 Demes and Optimization 14

8.8 The Optimize Template 14
8.9 Metapopulation 16
8.10 Metaoptimization 17
8.11 TODO: 17

9 MOSES: Putting It All Together 17

10 Final Remarks 18

1 Introduction

Meta-optimizing semantic evolutionary search (MOSES) is a new approach to program evolution, based on representation-building and probabilistic modeling. MOSES has been successfully applied to solve hard problems in domains such as computational biology, sentiment evaluation, and agent control. Results tend to be more accurate, and require fewer objective function evaluations, as compared to other program evolution systems. Best of all, the result of running MOSES is not a large nested structure or numerical vector, but a compact and comprehensible program written in a simple Lisp-like mini-language.

This document provides an overview of the core concepts, terminology, algorithm and capabilities of MOSES. The first few sections provide a general review, suitable for users and programmers alike, and should provide sufficient grounding to allow users to feel confident in this tool. The remiander of the document provides a quick overview of the internal structures of the code base, and is intended for programmers interested in exploring modified algorithms and extensions.

The main MOSES website/wiki is located at http://wiki.opencog.org/w/Meta-Optimizing_Semantic_Evolutionary_Search. Additional references can be found there, as well as in the References section at the end of this paper. Moshe Look's PhD thesis [6] is strongly recommended as primary material for anyone interested in additional details.

2 Copyright Notice

MOSES is Copyright 2005-2008, Moshe Looks and Novamente LLC.

It is licensed under the Apache License, Version 2.0 (the "License"); you may not use this file except in compliance with the License. You may obtain a copy of the License at

http://www.apache.org/licenses/LICENSE-2.0

Unless required by applicable law or agreed to in writing, software distributed under the License is distributed on an "AS IS" BASIS, WITHOUT WARRANTIES OR CONDITIONS OF ANY KIND, either express or implied. See the License for the specific language governing permissions and limitations under the License.

3 Overview

MOSES performs supervised learning, and thus requires either a scoring function or training data to be specified as input. As output, it generates a Combo program that, when executed, approximates the scoring function. MOSES uses general concepts from evolutionary search, in that it maintains a population of programs, and then explores the neighborhood of modified, "mutated" programs, evaluating their fitness. After some number of iterations, the fittest program found is output.

More precisely, MOSES maintains a population of demes. Each deme is a program with many adjustable, tuneable parameters. These adjustable parameters are colloquially referred to as knobs. Thus, finding the fittest program requires not only selecting a deme, but also determining the best settings for the knobs.

The MOSES algorithm proceeds by selecting a deme and performing random mutations on it, by inserting new knobs in various random places. The best-possible knob settings for the mutated deme are found by using using existing, well-known optimization algorithms, such as hill-climbing, simulated annealing or estimation of distribution algorithms (EDA) such as Bayesian optimization (BOA/hBOA). The fitness of the resulting program(s) can be compared to the fittest exemplar of other demes. If the new program is found to be more fit, it is used to start a new deme. Old dominated demes are discarded, and the process then repeats.

All program evolution algorithms tend to produce bloated, convoluted, redundant programs ("spaghetti code"). To avoid this, MOSES performs reduction at each stage, to bring the program into normal form. The specific normalization used is based on Holman's "elegant normal form", which mixes alternate layers of linear and non-linear operators. The resulting form is far more compact than, say, for example, boolean disjunctive or conjunctive normal form. Normalization eliminates redundant terms, and tends to make the resulting code both more human-readable, and faster to execute.

The above two techniques, optimization and normalization, allow MOSES to out-perform standard genetic programming systems. The EDA algorithms, by finding the dependencies in a Bayesian network, in fact are able to find how different parts of a program are related. This quickly rules out pointless mutations that change one part of a program without making corresponding changes in other, related parts of the program. The other important ingredient, reduction to normal form, allows programs to become smaller, more compact, faster to execute, and more human readable. Besides avoiding spaghetti code, normalization removes redundancies in programs, thus allowing smaller populations of less complex programs, speeding convergence.

The programs that MOSES generates are "generic", in the sense that MOSES works with structured trees, represented in Combo. Such trees can represent propositional formula, procedural or functional programs, etc. The core MOSES solver is written in C++, and takes the form of a library. There are many example programs illustrating how to use this library.

4 Terminology

MOSES uses a vocabulary specific to itself. Some of the most important terms are defined below.

Program. A *program*, in MOSES, is a *combo program.* A combo program is represented as a tree of operators, variables and values. Nodes in the tree may be constants (bits, integers, real numbers, *etc.*), boolean operators

(*and, or, etc.*), arithmetical operators (+, -, *, *etc.*), functions (*sin, cos, etc.*) or logical expressions (*if...then...else, etc.*), and so on. Arguments to an n-ary function are denoted with hash marks, so that $\#1, \#2, \ldots, \#n$ would be the arguments. Thus, for example, $(0 < (0.5 * \#1)) \vee \#2$ is a program that takes argument #1 (a float pt. number), multiplies it by 0.5, and checks to see if it is greater than zero. The result of this compare is or-ed ($\vee$-ed) with argument #2 (a boolean). Although programs may be explicit, as in this example, a program can also be understood to be a *representation*, together with a particular set of *knob settings*, as explained below.

Exemplar. An *exemplar* is a specific program; typically, the fittest one found.

Representation. A *representation* is a parameterized tree structure, representing a particular region of program space, centered around a single program (the *exemplar*). A representation is derived from the exemplar by inserting additional nodes in various (random) locations. The inserted nodes, however, are not specific values or functions or operators, but are rather place-holders for values/functions to be determined later. Each place-holder may be thought of as a *parameter*, and is colloquially referred to as a *knob*. A representation, together with a particular setting of the knobs, is equivalent to a program. During the optimization step in MOSES, the space of all possible parameter or knob settings will be explored, to locate the best possible settings, *i.e.* to find the fittest program.

Knobs. A *knob* is a single dimension of variation relative to a representation tree. It may be discrete or continuous. For example, given the program tree fragment $(0 < (0.5 * \#1)) \vee \#2$, a continuous knob might be used to vary the numerical constant 0.5 to other values. So, setting this knob to 0.7 would transform this tree fragment to $(0 < (0.7 * \#1)) \vee \#2$. Discrete knobs have a '*multiplicity*': the number of different possible settings they may have. Continuous knobs have an effectively infinite multiplicity; in practice, however, they are varied in steps of fractional powers of two.

A discrete knob with a multiplicity of 4 might be used to transform the boolean input #2, with 0 meaning 'always true', 1 meaning 'invert', 2 meaning 'don't invert' and 3 meaning 'always false'. So, setting this knob to 1 would transform the above example tree to $(0 < (0.5 * \#1)) \vee (\neg\#2)$. A discrete knob of multiplicity 6 might be used to replace the less-than comparison with $\leq, >, \geq, =$or $\neq$(thus making six possible comparison operators, for a multiplicity of 6 for this knob). Another discrete knob of multiplicity 3 might replace the '*or*' symbol $\vee$ with the '*and*' symbol $\wedge$ or the '*exclusive-or*' symbol $\otimes$. Knobs do not have to be defined as running over all possible values; it is usually convenient to keep multiplicity fairly low. This will usually help avoid excess redundancy in program space, although resulting programs may be more verbose.

Representation-building. The step in the MOSES algorithm where an exemplar is chosen, and a representation is constructed from it.

Instance. An *instance* is an array of particular knob settings. For compactness, instances are maintained as strings of bits; the description of which bit-fields correspond to which knob settings are kept in separate structures, the field set and the knob mapper. During optimization, an evolutionary algorithm will pick and choose among many different instances; a single field set and knob mapping suffices to describe them all.

Field set. The bits in the instance bit-string are organized into an array of *fields*. Each field corresponds to a single knob. The *field set* describes each field in the array: whether it is discrete or continuous, how many settings it may have, *etc.* The field set is divorced from the representation or any combo program: it is merely a listing of possible knobs, but does not indicate where those knobs are located in the representation.

Knob mapping. The *knob mapping* associates each field in a field set with the corresponding knob in the representation.

Neighborhood. The nearest neighbors of an instance are those other instances that differ by exactly one knob setting. This is called the *neighborhood at distance one*. With distance understood as the Hamming distance, one can then consider progressively larger neighborhoods: those that differ by just two knob settings, or three, *etc.*

Deme. A *deme* is a population of programs derived from one single representation. Thus, a deme can be thought of as a population of knob settings. During the optimization phase, an optimizer algorithm, such as hill-climbing, simulated annealing, or the Bayesian optimization algorithm is used to work with the population, locating the best possible knob settings for the given representation. In practice, in the actual implementation, a deme is just a set of scored instances. This is because all instances in a deme share the same representation, field set and knob mapping.

Metapopulation. MOSES maintains a collection of demes, playing each off the others. This set of demes is referred to as the *metapopulation*. Pairs of demes are in competition; fitter demes are used to replace less fit demes.

Scoring function. During the optimization phase, candidate programs being explored are scored by a *scoring function*. The function is specific to the given problem; it returns a value indicating how closely the candidate program matched the desired output. For supervised training (*aka* regression) problems, the scoring function just returns how closely the candidate program matched the training set. For demonstration problems, the scoring function is typically some well-studied toy problem, such as parity, one-max, santa-fe-trail, *etc.* Usually, the perfect score is 0, while worse scores are negative. Fitter programs have higher scores.

Domination. One program instance is considered to *dominate* another if it is better in every way. The concept of domination requires a scoring function that issues not just one grand-total score, but an array of scores. For example, for regression problems, a program instance may be judged on how accurately it provides an output given an input. To test this, one typically provides a table of N input rows, with each row indicating a desired output. The program can then be tested on each row, with the result compared to the desired output value for that row. One program is said to *dominate* another only if it has a better score on each of the N tests. Typically, two different programs do not dominate one-another: one is better for some input rows, while the other is better at others. In MOSES, both are kept around and further evolved, with the goal of eventually finding a program that dominates all. Programs that are completely dominated are (usually) discarded.

Normalization, reduction. The *normalization* step of the MOSES algorithm takes a program, and simplifies it, using *re-writing rules*. The resulting program is said to be in *normal form*. Thus, for example, $\#3 \vee F$ can be reduced to just $\#3$ since or-ing with false changes nothing. Similarly, $0 < 0.5 * \#6$ can be normalized to $0 < \#6$ since multiplying by one-half never changes the sign of a number. Likewise, the expression "if $(x = x)$ then y" can be reduced to y, since a value is always equal to itself, and so the if-branch is always taken. Normalization can sometimes eliminate large parts of a program, if they are vacuous or tautological. There are many different types of normalization that are possible; MOSES always normalizes to the so-called '*elegant normal form*'. The word '*reduction*' is often used as a synonym for normalization.

5 MOSES Algorithm

The MOSES algorithm consists of two nested optimization loops. The outer loop maintains a population of scored program trees, the so-called '*metapopulation*'. The inner loop explores a local neighborhood of a given program tree, using a representation centered on an exemplar. When the inner loop finds a reasonable set of candidate programs, these are returned to the outer loop, and merged back into the metapopulation. More precisely, the steps are as follows:

1. Selection step. Choose one exemplar from the metapopulation. Initially, this will be the empty program, unless the user specified an initial exemplar. The choice is made by considering the entire metapopulation, and picking the program tree with the highest score, that has not been previously explored (There is little point in re-exploring the neighborhood of a previously explored program tree, as all improvements are likely to have already been found). This selection is done by `metapopulation::select_exemplar()`.

2. Representation-building step. Given an exemplar, construct a representation; that is, take the exemplar and decorate it with knobs. Build a

field set and a knob mapper that will act as a mapping between a linear bit-string, and specific knob settings in the representation. The field set describes the layout of the bit-string; the knob mapper associates fields with knobs. Create an initial instance; that is, a bit-string that can be interpreted as a collection of knob settings, via the field set mapping.

3. Optimization step. Given a representation, a field set, and an initial instance, invoke the inner optimization loop. One of several different inner optimization loops are possible; they all have the steps below in common. Typically, a collection of scored instances is maintained; this collection is called a '*deme*', and thus the first step is to '*open a new deme*'.

   (a) Score the initial instance: that is, evaluate the combo program that results from these specific knob settings, and see how well this program reflects the desired regression output.

   (b) Generate new instances via some algorithm (*e.g.* hill-climbing, simulated annealing, *etc.*) and score these instances in turn. Maintain a collection of scored instances; these are referred to as the *deme*. New instances are typically neighbors of other instances: they differ from existing instances by just a few knob settings.

   (c) Terminate the search via some exit criteria: lack of improvement, number of allowed evaluations exceeded, maximal neighborhood explored, *etc.*

4. Close the deme. This step accept the list of best-possible instances found in the previous step, and merges them back into the metapopulation. First, convert each instance in the deme back into ordinary program trees (*i.e.* by fixing knob settings at a set position, thus 'removing' the knobs). Normalize, or reduce each of these to 'elegant normal form'. Merge the resulting programs into the metapopulation, ranking them by score. Merging is performed by considering domination; dominated programs are discarded; non-dominated ones are added to the metapopulation.

5. Go to step 1, repeating until termination criteria are met, such as achieving perfect score, or exceeding the maximal number of evaluations, *etc.*

Note that representation-building, the optimization algorithm, and normalization are vital steps of the algorithm, and they crucially influence its performance. Representation building is specific for each domain (e.g., learning propositional formulae), while the optimization algorithm is general (it operates only on instances). MOSES currently supports representation building for several problem domains, including propositional formulae, actions[1], arithmetic formulas, and predicate logic (arithmetic relations embedded in propositional formulae.

[1]By "actions" we mean mini programming languages describing actions of a agents such as artificial and [6]. Available actions typically cover atomic instructions like "step forward", "rotate left", "rotate right", "step forward", branching instruction such as „if-then-else“, and loop instruction such as „while“.

MOSES also supports several different optimization algorithms, including hill-climbing, simulated annealing and Bayesian optimization. Work on support-vector machine (SVM) optimization is underway. Only one form of program reduction, to elegant normal form, is supported. Other types of reduction, e.g. SAT-based or satisfiability-modulo-theory (SMT) may be possible but remains unexplored.

6 Installation

To compile MOSES, you need

- a recent gcc (4.x or later);
- the boost libraries (http://www.boost.org/);
- the CMake package (http://www.cmake.org/HTML/Index.html);

For compiling MOSES, create a directory `build` (from the root folder of the MOSES distribution), go under it and run "`cmake ..`". This will create the needed build files. Then, make the project using "`make`" (again from the directory `build`). Generated executables will be in the folder `build/moses/learning/moses/main`.

7 Source Files and Folders

MOSES is implemented in C++ and makes heavy use of templates. Modifying MOSES requires familiarity with C++ and, at least to some extent, with C++ templates.

The following folders can be found in the MOSES distribution, under `moses/learning/moses`:

eda This folder contains support for estimation of distribution algorithms, and the lower level support for optimization algorithms.

example-ant Example implementation of using MOSES to solve for robotic perception-action algorithms. This example demonstrates the "ant on the Santa Fe Trail" problem; it includes definitions for the movement of the ant, the perceptions of the ant, and the space in which the ant can move. Conceptually, the problem is that of finding the best possible algorithm for a robot to use to accomplish some task, given that the robot has certain limited senses, and certain limited movements.

example-data Contains example data sets, illustrating regression (supervised training).

example-progs This folder contains examples demonstrating different features of the MOSES system: reducing expressions, and, of course, applications of MOSES itself. It includes examples for the, for "parity formulae", *etc.*

main Contains the main moses executable.

moses This folder contains the core support for MOSES – including base type definitions, and distributed computation support.

optimization Contains the main optimization code.

representation Contains code for representation-building, that is, for taking an exemplar, attaching knobs to it, and converting the knob settings to and from bit-strings that the optimizer expects to work on.

8 Types, Structures, and Classes

This section briefly reviews some of the key datatypes and classes found in the code. MOSES makes heavy use of C++ templating. This is done so as to avoid the need for defining base classes, and so avoid the need for pervasive use of derived classes and virtual methods.

8.1 Structured expression trees

For representing structured expression trees (programs, propositional formulae, *etc.*) MOSES relies on the library ComboReduct. In this library, structured expressions are represented by trees of the type `combo tree` as follows:

```
typedef Util::tree<vertex> combo_tree;
```

The file `comboreduct/combo/vertex.h` defines `vertex` as shown blow. It is done this way so that it can capture different sorts of nodes, for different, but still fixed, problem domains.

```
typedef boost::variant<builtin,
       wildcard,
       argument,
       contint,
       action,
       builtinaction,
       perception,
       definiteobject,
       indefiniteobject,
       message,
       procedurecall,
       anntype,
       actionsymbol> vertex;
```

For more information, review the docs provided in the distribution of the ComboReduct library.

8.2 Representation building

Conceptually, representation building proceeds by selecting a single program tree or exemplar, and then building a neighborhood of nearby programs. The

result is a deme: a population of similar programs, centered upon the exemplar. After representation building, the optimization step is performed, to find the fittest programs in the deme.

Representation building proceeds in several steps: the exemplar is festooned with a set of 'knobs': these are adjustable parameters added to the program tree, to alter its operation. An exemplar festooned with knobs is called a `'representation'`. Each of these steps is reviewed in greater detail, below.

The representation itself is specified by the `class representation`, given in `representation/representation.h`. The structure itself maintains a copy of the exemplar, as well as the collection of knobs used to create the representation. Knobs are added to the exemplar by the class constructor. The knob mapping relates locations in the representation to fields in the field set. The field set indicates which bits in the bit-string instance correspond to a field.

The `representation` class does not maintain the set of possible knob settings (the instances); these kept in the deme, and are held elsewhere.

8.3 Knobs

Knobs represent tunable parameters in a representation. In the MOSES implementation, every knob is defined with respect to a particular program tree; that is, ever knob has a specific, explicit location in a specific, explicit `combo_tree`. Knobs are defined in `representation/knobs.h` and inherit from the base class `knob_base`:

```
struct knob_base {
  protected:
     combo_tree& _tr;
     combo_tree::iterator _loc; // location of knob in tree
};
```

Knobs may be continuous (`contin_knob`) or discrete. Discrete knobs have a fixed number of settings, determined at compile time:

```
template<int Multiplicity>
struct discrete_knob : public knob_base {...};
```

although some of the discrete knob settings may be disallowed at runtime, effectively decreasing the total multiplicity.

In general, knobs may be "present" or "absent". XXX The semantics of this is unclear. What does this mean? Knobs also have "default" settings, but the meaning of this setting is also unclear, as the code does wonky things with this... XXX TODO fix this.

The `logical_subtree_knob` is suitable for propositional formulae; it has three possible settings: *present*, *absent*, or *negated*. In `simple_action_subtree_knob` knobs, a subexpression can be just *present* or *absent*. In action knobs, a node can have different settings, corresponding to atomic or compound actions, sampled as "perms" in the method `build_knobs::sample_action_perms`. XXX more explanation please ...

Knobs are added to an exemplar using `class build_knobs` defined in `representation/build_knobs.h`.

8.4 Packed Knobs, Instances

In order for knob settings to be efficiently managed by the optimization step, they are packed into a bit-string; the bit-string is of type `instance`, declared in `eda/eda.h` as a vector of ints:

```
typedef unsigned int packed_t;
typedef vector<packed_t> instance;
```

The class `field_set` describes the manner in which knobs are packed into the bit string. Defined in `representation/field_set.h`, it contains a list of fields with describe the width of a field, and it's offset within the bit-string:

```
struct field {
   width_t width;
   size_t major_offset, minor_offset;
};
```

Fields come in four basic types: continuous, discrete, "terms", and single-bit booleans. Discrete fields represent variables that can take on multiple distinct values. These typically take several bits to represent; single-bit boolean variables are treated distinctly. Continuous variables can take on values in the real-number line. However, they are not represented by floats or doubles, but rather, are represented with a certain binary tree of intervals of the real-number line. This encoding is used in order to avoid various difficulties that optimization algorithms encounter with floats and doubles. The rationale and design is further discussed here: http://code.google.com/p/moses/wiki/ModelingAtomSpaces. "Term" fields refer to the tree structure of term algebras: they can be thought of as trees whose nodes are labeled with strings. Term algebras, also known as "absolutely free algebras", are commonly used in logic, category theory, universal algebra and programming to represent arbitrary data structures. From the standpoint of MOSES, they generalize the binary tree structure of the '`contin`' variables.

The `field_set` provides a set of iterators for walking over these four types; the iterators can be used to extract and change specific values in the bit-string, in the usual fashion. That is, dereferencing an iterator gives the value. So for example, the `disc_iterator` can be used to iterate over discrete fields in an instance:

```
const_disc_iterator begin_disc(const instance& inst) const;
const_disc_iterator end_disc(const instance& inst) const;

instance_t my_inst = ...;
const_disc_iterator it = begin_disc(my_inst);
for ( ; it<end_disc(my_inst); it++) {
   cout " This is the value: " << *it << endl;
}
```

Typically, many different instances can be described by the same `field_set`. Thus, when an `instance_set` is defined (see below), one copy of the `field_set` is kept, to describe all of the instances in an `instance_set`.

These unpacked fields are associated with actual knobs in a specific `combo_tree` by means of the `knob_mapper`. So, for example

```
typedef std::multimap<field_set::disc_spec, disc_knob> disc_map;
```

is a map between `disc_knob`'s (discrete knobs, which are aware of the `combo_tree` in which they are located), and `disc_spec`'s (knob specifications, which are used by `field_set` to describe fields in the packed bit-string). This map can be used to find a knob value in a packed bit-string, or, conversely, given a field, to find the corresponding knob in a `combo_tree`.

8.5 Representation, revisited

XXX some details below incorrect.

The constructor of this structure, builds knobs with respect to the given exemplar (by the method `build_knobs`).

This structure stores the exemplar like a tree (more precisely `combo_tree`). This structure has a method for using a given instance to transform the exemplar (`transform`) providing a new expression tree.

The structure also has methods for clearing the current version of the exemplar (setting all knobs to default values — zeros) — `clear_exemplar`, for getting the exemplar — `get_clean_exemplar`, and for getting the reduced, simplified version of the exemplar `get_clean_exemplar`.

8.6 Scoring

The fitness of program trees are ranked with scores. There are several types of scores, these are all defined in `moses/types.h.` The most basic is

```
typedef float score_t;
```

Different programs may be code at different things, and so judging their fitness in multiple ways requires a vector:

```
typedef std::vector<score_t> behavioral_score;
```

Programs are also scored according to their complexity, so in `moses/complexity.h` we find:

```
typedef int complexity_t;
```

Composite scores pair up the complexity and the fitness score:

```
typedef std::pair<score_t, complexity_t> composite_score;
```

while behavioral composite scores combine the complexity measure with the vector:

```
typedef tagged_item<behavioral_score, composite_score>
    composite_behavioral_score;
```

XXX Disconnect .. the optimize() template in eda/optimize.h doesn't take any of these, but a generic scoring policy which can return anything (and maintain anything internally, by being a class that inherits from unary_function<instance,retval> and implementing operator>(). So. ahh explain.... also, a number of example scoring functions in example-progs/scoring_functions.h

8.7 Demes and Optimization

At the conceptual level, the optimization algorithms operate on a population in a deme, by twiddling knobs until the optimizer finds a program that performs well. That is, a deme is a collection of knob settings: for evolutionary optimization algorithms, it may be thought of as a population: different instances of knob settings compete with one-another until a population of the best-possible knob settings is found.

At the practical level, optimization operates on a population of bit-strings, and not on abstract 'knob settings'. Thus, a necessary step of the MOSES algorithm is to convert such abstract knob settings into packed bit strings, and *vice versa*. This is done during the representation step of the algorithm: knobs were inserted into an exemplar, the knobs were mapped to knob specifications, and the knob specifications were in-turn mapped to bit-strings. The optimization algorithm then tries to find the fittest bit-strings or 'instances'.

So, in `eda/scoring.h`, we find scored instances. The higher the score, the fitter the bit-string:

```
template<typename ScoreT>
struct scored_instance :
    public tagged_item<instance, ScoreT> {...};
```

In `representation/instance_set.h` we find a collection of scored instances:

```
template<typename ScoreT>
struct instance_set :
    public vector<scored_instance<ScoreT> > {
    ...
    protected:
        const field_set &_fields;
};
```

Note that, in the above, there is a `field_set` member used to describe how the packed bits in the instance are to be unpacked.

8.8 The Optimize Template

These file `eda/optimize.h` defines a function template that implements a generic evolutionary selection algorithm. Currently, this template is used to implement only one optimization algorithm, the Bayesian univariate algorithm. Although

MOSES also implements hill-climbing and simulated annealing, neither of the latter two make use of this template (although they probably should, as otherwise there is a large amount of cut-n-paste code duplication, leading to code bloat and making maintenance harder ...)

```
template <typename ScoreT,
       typename ScoringPolicy,
       typename TerminationPolicy,
       typename SelectionPolicy,
       typename StructureLearningPolicy,
       typename ProbsLearningPolicy,
       typename ReplacementPolicy,
       typename LoggingPolicy>
int optimize(instance_set<ScoreT>&, ...);
```

The algorithm itself is meant to be generic: it is a loop of steps performed on a population. The different policies determine how the different steps of the algorithm are actually carried out.

Before entering the loop, each individual in the population is scored for fitness (using XXX??). Inside the loop, the following steps are taken:

1. A number of individuals are selected, using `SelectionPolicy`. The selected individuals will be modeled and then entered into the tournament. (XXX what pre-defined selection policies to we have??)

2. Initialize a model, using the specified `StructureLearningPolicy`. Currently, only one policy is pre-defined: `univariate()`, which creates the trivial structure, *viz.* no structure at all. The univariate policy assumes no interdependency at all between different genes (variables) in the population: its a no-op. The BOA policy, (XXX which needs to be ported over from older code XXX), will build a full Bayesian network of dependencies.

3. Learn the structure. The idea here is that the different variables in the problem are not independent, but are related to one-another: this is the "structure" of the problem. This step gives the solver the opportunity to discern that structure. So, for example, the Bayesian Optimization Algorithm (BOA) assumes that the structure is a network of probabilities, a Bayesian network. As this algorithm is generic, any structure learning system may be used; that is, this step invokes the `StructureLearningPolicy.` Only the selected individuals, from step 1, are used for this.

4. Learn the probability distribution for the selected individuals. The learning here is done within the context of the previously learned structure. Again, the presumption is that, once the dependencies between variables are known, then understanding the actual distribution in a population will hint at were optimal solutions lie. This function is of type `ProbsLearningPolicy.`

5. Create a fixed number of new individuals or instances. These are created based on the distribution learned in the previous step. The goal here is to create more individuals that are likely to have a high score.

6. Score the new individuals, using the `ScoringPolicy`. This step determines the fitness of the newly created individuals. The scoring policy is, by definition, highly problem-specific. A number of different example scoring policies can be found in `example-progs/scoring_functions.h`.

7. Replace segments of the existing population with the newly created individuals. This is done with the `ReplacementPolicy`. Several different replacement policies are defined in `eda/replacement.h`: the `replace_the_worst()` policy unconditionally replaces the lowest-scoring members of the population. The `rtr_replacement()` replaces the most similar members, based on the hamming distance between the fields.

8. Repeat. Go to step 1, unless the maximum generation count has been exceeded, or if the `TerminationPolicy` has been met. There is no point in iterating if a good-enough solution has already been found; the `TerminationPolicy` determines what is considered to be "good enough". Currently, two types of termination are pre-defined, in `eda/termination.h`: one is `terminate_if_gte()`, which ends when scores exceed a bound, and `terminate_if_gte_or_no_improv`(), which ends when scores exceed a bound, or fail to show improvement.

Note that `ScoringPolicy` needs to be thread-safe, as it's `operator()` will be invoked from multiple threads.

8.9 Metapopulation

The metapopulation is a set of scored combo trees. More precisely, they are scored with composite behavioural scores (or b-scores). A 'bscored combo tree' is then just a pair that associates a b-score with a tree, defined in the file `moses/types.h`.

```
typedef tagged_item<combo::combo_tree,
      composite_behavioral_score> bscored_combo_tree;
```

The metapopulation is then a set of scored combo trees, defined in `moses/metapopulation.h`. More precisely, it is a template, inheriting from the set:

```
typedef std::set<bscored_combo_tree> bscored_combo_tree_set;

template<typename Scoring,
         typename BScoring,
         typename Optimization>
struct metapopulation :
   public bscored_combo_tree_set {...};
```

The template plays only a small role in this class; it's only purpose is to allow generic scoring and optimization algorithms to be used with the metapopulation.

The metapopulation will store expressions (as scored trees) that were encountered during the learning process (not all of them; the weak ones, which are dominated by existing ones, are usually skipped as non-promising).

As an example, one can iterate through the metapopulation and print all its elements with their scores and complexities in the following way:

```
for (const iterator it=begin(); it!=end(); ++it)
   cout << gettree(*it) << " "
      << getscore(*it) << " "
      << getcomplexity(*it) << endl;
```

The metapopulation is updated in iterations. In each iteration, one of its elements is selected as an exemplar. The exemplar is then used for building a new deme (that will further extend the metapopulation).

8.10 Metaoptimization

The main metaoptimization step is carried out by the `metapopulation::expand()` method. This method implements three steps: `create_deme()`, followed by `optimize_deme()`, followed by `close_deme()`. The `optimize_deme()` step invokes the low-level optimizer for the deme (i.e. invokes either `univariate_optimization()`, `simulated_annealing()` or `iterative_hillclimbing()`.

XXX discuss domination.

8.11 TODO:

Discuss role of tree_type in knobs.

9 MOSES: Putting It All Together

With all components briefly described above, this section discusses how are they combined in the MOSES system. XXX The contents below are stale, and need to be re-written.

The main moses method is trivial: it expands the metapopulation in iterations until the given number of evaluations or a perfect solution is reached. This method is implemented in `moses/moses.h`, in several variations (some with additional arguments corresponding to available actions and perceptions, just for the action problem domain).

Typical usage of MOSES starts by providing scoring functions. For instance, for learning disjunction propositional formula one can use the following declaration (defined in `moses/scoring_functions.h`):

```
disjunction scorer;
```

and for solving the ant problem, one can use the following declaration (defined in `moses/scoring_functions.h`):

```
antscore scorer;
```

Also, the type of expression to be learnt has to be provided [2]. For instance, for the disjunctive formula, one should use:

```
typetree tt(id::lambdatype); tt.appendchildren(tt.begin(),id::booleantype,arity+1);
```

where `arity` carries the information of the number of propositional variables to be considered. For the ant problem, one would write:

```
typetree tt(id::lambdatype); tt.appendchildren(tt.begin(),id::actionresulttype,1);
```

Then the metapopulation has to be declared. It is instantiated via templates, saying which scoring function, which behavioral scoring function, and which optimization algorithm to use. As, arguments one has to provide the random generator, the initial exemplar, the type tree, simplification procedure, then the scorers and the optimization algorithm. This is an example for learning the disjunctive formula:

```
 metapopulation<logicalscore,logicalbscore,univariateoptimization>
metapop(rng, vtree(id::logicaland),tt,logicalreduction(), logicalscore(scorer,arity,rng),
logicalbscore(scorer,arity,rng), univariateoptimization(rng));
```

and this is an example for the ant problem:

```
 metapopulation<antscore,antbscore,univariateoptimization>
metapop(rng,vtree(id::sequentialand),tt,actionreduction(), scorer,
bscorer, univariateoptimization(rng));
```

10 Final Remarks

While MOSES is not that big a system, it cannot be documented in detail in just a few pages. However, the descriptions given above should be helpful when one first encounters MOSES and tries to use it and modify it.

Currently, MOSES together with ComboReduct consists of 17 KLOC of .cc files and 24 KLOC of header files, as counted by the wc command. This includes all comments, copyright notices, example programs and utilities. Of this, combo consists of about 18 KLOC while MOSES consists of 22 KLOC.

[2]for a detail explanation of the type system used in ComboReduct see the doc provided with the distribution of ComboReduct

References

[1] Moshe Looks, "Scalable Estimation-of-Distribution Program Evolution", Genetic and Evolutionary Computation Conference (GECCO), 2007.

[2] Moshe Looks, "On the Behavioral Diversity of Random Programs", Genetic and Evolutionary Computation Conference (GECCO), 2007.

[3] Moshe Looks, "Meta-Optimizing Semantic Evolutionary Search", Genetic and Evolutionary Computation Conference (GECCO), 2007.

[4] Moshe Looks, Ben Goertzel, Lucio de Souza Coelho, Mauricio Mudado, and Cassio Pennachin,"Clustering Gene Expression Data via Mining Ensembles of Classification Rules Evolved Using MOSES", Genetic and Evolutionary Computation Conference (GECCO), 2007.

[5] Moshe Looks, Ben Goertzel, Lucio de Souza Coelho, Mauricio Mudado, and Cassio Pennachin, "Understanding Microarray Data through Applying Competent Program Evolution", Genetic and Evolutionary Computation Conference (GECCO), 2007.

[6] Moshe Looks, "Competent Program Evolution" Doctoral Dissertation, Washington University in St. Louis, 2006.

[7] Moshe Looks, "Program Evolution for General Intelligence", Artificial General Intelligence Research Institute Workshop (AGIRI), 2006.
