# Supplementary material for: Predicting the Risk of Suicide by Analyzing the Text of Clinical Notes
Source: PLoS One. 2014 Jan 28;9(1):e85733. doi: 10.1371/journal.pone.0085733 (PMC3904866; doi:10.1371/journal.pone.0085733)
Supplement: Appendix S3 — Machine Learning Libraries and Methods: This section is provided to enclose the open source (Apache License) classifier used for the building of the statistical models, specifically for the purposes of study reproducibility. A detailed account of these tools is intended for another publication. (ZIP) [file pone.0085733.s003.zip › Appendix3/ML_libs/moses-3.4.0.tar.bz2!/moses-3.4.0/doc/comboreduct/ComboReduct.pdf]

Developer and User Manual of ComboReduct (draft)

Nil Geisweiller

November 3, 2008

Contents

1 Introduction **2**

2 Installation **2**

3 Folder overview **2**

4 Combo language **3**

5 Type checker engine **5**
5.1 Type representation 5
5.2 Implicit vs explicit inputs 6
5.3 Arity 6
5.4 Type inference 6
5.5 Type checking 7

6 Reduct engine **7**

1 Introduction

ComboReduct is a library providing C++ data structures and algorithms to manipulate a programatic language used in program evolution like MOSES. That code was initialy integrated in MOSES but has been split to be reused in other program evolution project.

2 Installation

For compiling MOSES you need

- a recent gcc (4.x or late 3.x)
- the boost libaries (`http://www.boost.org`)
- the CMake package (`http://www.cmake.org/HTML/Index.html`)
- the LADSUtil library (TODO : place the urls)

For compiling Comboreduct, create a directory `bin` (from the root folder of the ComboReduct distribution), go under it and run `"cmake .."`. This will create the needed build files. Then, make the project using "`make`" (again from the directory `bin`). Then `make install` (you may need the root provilege) to install it.

More detailed information about the intallation can be found in INSTALL text file in the root directory.

3 Folder overview

In this section we give a bief explanation of the project directory tree. At the root we have:

- `doc` containing that manual
- `sample` containing a few files with thousands of combo expressions used to test the reduct engin
- `scripts` containing a script `run_utest.sh` to run test units
- `src` containing the source code of the library
- `test` containing the test unit code

Under `src/ComboReduct` the code is organized in the following folders:

- `ant_combo_vocabulary` containing an example of the use of ComboReduct in the context of the ant problem
- `combo` containing the code defining the data structure of combo programs and the type checker engine.

- `crutil` containing some common definitions
- `main` containing a bunch of executable to test ComboReduct functionalities
- `reduct` containing the code of the Reduct engine

Now we will go through each component of ComboReduct, that is Combo a programatic language, its type checker and the Reduct engine.

4 Combo language

Combo is a programatic language dedicated to program evolution. It contains primitives to handle boolean operators, arithmetics and action/perception of an iteractive agent.

A Combo program is represented by a tree where each node is either an operator, a constant or a procedure call and where operands of operators are its children.

The C++ structure for it (the C++ code in under src/ComboReduct/combo/vertex.h) is:

```
typedef tree<vertex> vtree;
```

where `vertex` is defined as a boost union type (variant):

```
typedef boost::variant<builtin,
                       wild_card,
                       argument,
                       contin_t,
                       action,
                       builtin_action,
                       perception,
                       definite_object,
                       indefinite_object,
                       message,
                       procedure_call,
                       action_symbol> vertex;
```

- `builtin` is a enumaration of standard boolean-arithmetic operators and constants, like `and`, `or`, `+`, `log`, etc.
- `wild_card` represented by `_*_` is used for unification when the program output type is boolean.
- `argument` is a structure representing input arguments into a Combo program, noted `#1`, `#2`, etc. For instance the following program (prefix representation) `+(#1 #2)`, is the addition of two input arguments.

- `contin_t` is a float type.
- `action` is a pointer to an abstract class `action_base` (see `src/ComboReduct/combo/action.h`) to be implemented representing the set of actions controled by the interactive agent, like for instance `eat_food_ahead`. The implementation of `action_base` can also provide methods defining properties like, for instance, if an actions always succeeds or is idempotent, etc. That set of properties can be later used by the reduction engine to normalize Combo programs containing user-defined actions.
- `builtin_action` is an enumeration of basic action operators like `and_seq` (executes a sequence of actions until one fails or the sequence is completed), `boolean_while` (execute an action repeatedly while a condition is met). For instance

  ```
  boolean_while(is_hungry and_seq(look_for_food eat_food))
  ```

  is a program that executes in sequence searching for food and eating food until hungriness has gone.
- `perception` is a pointer to an abstract class `perception_base` (see `src/ComboReduct/combo/percept`[illegible] to be implemented representing the set of perceptions of an interactive agent, like `is_hungry`. The implementation of `perception_base` can also provide methods defining properties like, for instance, if the perception arguments are symetric or reflexif. That set of properties can be later used by the reduction engine to normalize Combo programs containing user-defined perceptions.
- `definite_object` is a pointer to an abstract class to be implemented representing the set of definite objects existing in the world of the interacting agent, like `red_cube`, `green_ball`. It is essentially a C++ string representing an identifier, that is not containing space or seperator symbols.
- `indefinite_object` is a pointer to an abstract class to be implemented representing the set of indefinite objects pointing to definite object existing in the world of the interactive agent, like `random_ball`, `nearest_cube`.
- `message` is class containing a message, it is a C++ string that can contain any symbol in it including space and other separators.
- `procedure_call` is a pointer to a Combo program, Combo handles recursive and mutually recursive procedure calls. ComboReduct can load a set of procedures. The syntax used is

  ```
  procedure_name(arity) := procedure_body
  ```

- `action_symbol` is a pointer to an abstract class to be implemented representing symbols used to caracterize actions, for instance in the context of a virtual pet with the action `scratch` the action symbol `neck` can be used to form `scratch(neck)`

5 Type checker engine

5.1 Type representation

ComboReduct contains a type checker engine, to check and infer types. A type is also a tree just like a program but the nodes referes to type operators and type constants instead of actual operators and constants. So type_tree is defined as follow (the code can be found at `src/ComboReduct/combo/type_tree_def.h`):

```
typedef tree<type_node> type_tree
```

Where type_node is a C++ enumeration:

```
enum type_node {
  lambda_type,
  application_type,
  union_type,
  arg_list_type,
  boolean_type,
  contin_type,
  action_result_type,
  definite_object_type,
  action_definite_object_type,
  indefinite_object_type,
  message_type,
  action_symbol_type,
  wild_card_type,
  unknown_type,
  ill_formed_type,
  argument_type
};
```

There are 4 type operators, `lambda_type` to represent the abstraction of a function (like in λ-calculus), `application_type` to represent the application of a function (like the application operator of λ-calculus), `union_type` to represent the union of types and `arg_list` type to arbitrary large list of a given type. The other types are rather self-explanatory but more explanation can be found in the comments of the code.

So example a boolean function or arity 3 will be represented with the following type (`_type` suffix are omited for clarity):

```
lambda(boolean boolean boolean boolean)
```

The first 3 `boolean` design the input types and the last `boolean` designs the ouput type. For instance the combo program `and(#1 #2 #3)` has the type above.

5.2 Implicit vs explicit inputs

A combo program does not necessarily need to use explicitely arguments `#1`, `#2`, etc, to define its inputs. A combo program is treated just like an operator, for instance, `not(boolean_if)` will be treated as the function that takes 3 boolean inputs and returns the negation of the result of a boolean_if applied on them. The type tree of such combo program is therefore

```
lambda(boolean boolean boolean boolean)
```

We say that just combo program has implicit inputs. As such any operator alone is a combo program with implicit inputs. Some operators uses an indefinite number of the inputs of the same type (like `+`), there input types are described by using the type operator `arg_list`, for instance the type tree of `+` is

```
lambda(arg_list(contin) contin)
```

A combo program can be made from both explicit (`#1`, `#2`, etc) and implicit inputs the convention is that the explicit inputs are always enumerated at first in the input list, no matter the order they appear in the combo program. For instance the type of `contin_if(and #1 #2)` has type tree

```
lambda(contin contin arg_list(boolean) contin)
```

5.3 Arity

There is a subtlety in the calculation of the notion of arity in the type system of ComboReduct which is worth mentioning here. When the number of input arguments are fixed, that is the combo program takes exactly n input arguments then the arity is n. On the other hand if the number inputs are $n-1$ or more (that is the type operator `arg_list` is involved in the list of the input types) then the arity is $-n$.

5.4 Type inference

Type inference is performed by converting a Combo tree into a correspondant type tree representing the abstractions and applications of the operators with there operands, then that tree is reduced into a normal form to look more like what we can call a procedure type signature.

For instance, the type tree of `+(3 5)` is

```
application(lambda(arg_list(contin contin) contin contin)
```

then we apply a normalizer that reduce the application of function with inputs (a la λ-calculus when a redex is eliminated by β-reduction) to obtain the type `contin`.

There are of course several subtleties in that process which could be long to describe in detail but many comments have been placed in the code explaining that reduction process in detail.

5.5 Type checking

Type checking is operated by evaluating the inheritance between two types. So for instance if one wants to check that the combo program

```
contin_if(and(#1 #2) 3 5)
```

it will first infer its type using the type inference engine, which is

```
lambda(boolean boolean contin)
```

then check if the infered type inherits the type we want the expression to have. For instance if the type to check the expression against is

```
lambda(boolean boolean union(contin boolean))
```

then the inheritance checker will be able to assess that (the answer positive in that example).

Once again the details and the assumptions made in the that process are numerous and not described here but the code contains the comments that explain them all in detail.

6 Reduct engine

One of the powerful aspects of ComboReduct is the Reduct engine, in charge of reducing a combo program into a normal form, that is into a unique semantically equivalent program. This is of great interest in program evolution as it, in some way, factorizes the syntactic space into its semantics' therefore avoiding reevaluating semantically equivalent programs. That is said it is important to note that such normalization process is not feasable in general, indeed answering whether two recursive functions are semantically equivalent is undecidable (and even so does answering the equivalence of two primitive recursive functions).

But should even normalizing be possible in theory, in practice it has to be fast, in the sense that the total cost of normalizing should be lower than the cost of re-evaluating semantically equivalent programs for a given search. Fortunately the reduct engine has been coded to be quite flexible and it is easy to craft a reduction process for a given problem -by combining a set of hard coded reduction rules- to get a good compromise between completeness and speed.

The source files at `src/ComboReduct/reduct` with suffix `_rules` contain the code and the detailed explanation of the different rules, and the sources files with suffix `_reduction` examples of how to combined thoses different hard-coded rules.
